# Spatial–temporal trends in global childhood overweight and obesity from 1975 to 2030: a weight mean center and projection analysis of 191 countries

**DOI:** 10.1186/s12992-023-00954-5

**Published:** 2023-08-04

**Authors:** Liwang Gao, Wen Peng, Hong Xue, Yang Wu, Haixia Zhou, Peng Jia, Youfa Wang

**Affiliations:** 1 School of Public Health, Capital Medical University, Beijing, China; 2 Global Health Institute, School of Public Health, Xi’an Jiaotong University Health Science Center, Xi’an, China; 3 Nutrition and Health Promotion Center, Department of Public Health, Medical College, Qinghai University, Xining, China; 4 International Obesity and Metabolic Disease Research Center, Xi’an Jiaotong University, Xi’an, China; 5 Department of Health Administration and Policy, College of Public Health, George Mason University, Fairfax, VA USA; 6 Department of Sociology, Jiangxi University of Finance and Economics, Nanchang, China; 7 School of Resource and Environmental Sciences, Wuhan University, Wuhan, China; 8 International Institute of Spatial Lifecourse Health (ISLE), Wuhan University, Wuhan, China

**Keywords:** Overweight and obesity, Trends, Projection, Children and adolescents, Global

## Abstract

**Background:**

The geographic information science-based interactive map provided good prospects for the public health to study disease prevalence. The purpose of this study is to understand global spatial–temporal trends of childhood overweight and obesity and underlying causes help formulating intervention strategies.

**Methods:**

This multiple cross-sectional study included data on childhood overweight and obesity prevalence, gross national income per capita, and urbanization rate for 191 countries from 1975–2016. Autoregressive integrated moving average model, standard deviational ellipse model and mixed-effects models were used to explore spatial–temporal trends of childhood overweight and obesity and associations with gross national income per capita and urbanization rate.

**Results:**

Globally, childhood overweight and obesity rate would reach 30.0% in 2030 (boys: 34.2%, girls: 27.4%). By 2030, it would reach 58.3% in middle- and high-income countries and 68.1% in Western Pacific region. Spatial–temporal trendline for childhood overweight and obesity in 1975–2030 exhibited a “C” shape, migrating from 1975 (15.6^。^E, 24.6^。^N) to 2005 (10.6^。^E, 21.7^。^N), then to 2030 (14.8^。^E, 17.4^。^N). The trendline for urbanization rate was also an irregular "C", and the turning point appeared five years earlier than childhood overweight and obesity.

**Conclusions:**

Globally, childhood overweight and obesity prevalence will continue to increase. Its weight mean center migrated from western countries to Asia and Africa following economic development.

**Supplementary Information:**

The online version contains supplementary material available at 10.1186/s12992-023-00954-5.

## Introduction

The prevalence of childhood overweight and obesity (ow/ob) hasbeen increasing worldwide over the past four decades [1]. This indicates increased risks for many other chronic diseases in adulthood, such as coronary heart disease [2, 3]. It also lead to economic consequences, the economic costs of childhood ow/ob were estimated as 14.1 billion dollars in 2010 in Germany, Spain and Italy [4]. Therefore, the prevention and control of childhood ow/ob have important public health significance and economic benefits.

To tackle the ow/ob, the first thing we need to do is to fully understand the prevalence and future trends of childhood ow/ob. In 2010, Mercedes et al. published global estimates prevalence and trends of ow/ob among preschool children, and it also emphasizes the importance of monitoring the epidemic trend of childhood ow/ob [5]. Our previous research estimated that there were 43 million ow/ob children worldwide (35 million in developing countries) in 2012 [6]. The latest studies showed that the time trends in children's and adolescents' body mass index (BMI) have plateaued in many high-income countries, but have accelerated in parts of Asia [7]. However, there is no research that comprehensively analyzes the temporal and spatial trends of the global childhood ow/ob, and the projection for school-aged children is also lacking.

The geographic distribution and its time trends of childhood ow/ob are important factors for global resource allocation. Childhood ow/ob used to be considered as a problem only in high-income countries. Nevertheless, recent studies showed the prevalence had been rising also in low- and middle-income countries such as India, China, and countries in sub-Saharan Africa [8]. The modernization theory of obesity showed that globalization and modernization were inevitably lead to changes in socio-economic conditions, including increased disposable income and urbanization rate, and changed the lifestyles, thus affecting the occurrence and development of children obesity, and this development included changes in the prevalence of childhood obesity over time and geography [9]. Despite of the efforts in collecting global data by the NCD Risk Factor Collaboration and some other groups in recent years [1, 10], we did not find any study which examined the spatial trend of global childhood ow/ob over time. The geographic information science(GIS)-based interactive map was used to visualize economic indicators in different geographic regions and their changes over time, which provided good prospects for the public health to study disease prevalence. But the application of GIS data/methods in childhood ow/ob research remains limited [11].

To fill the research gaps, this study aimed to 1) project the global ow/ob prevalence among school-aged children till 2030; 2) assess the global spatial–temporal patterns of childhood ow/ob from 1975 to 2030; and 3) examine possible reasons affecting the trend of childhood ow/ob, from the perspective of economic globalization.

## Methods

### Sample

This study used childhood ow/ob data from the Global Health Observatory (GHO) database, which is the interface between World Health Organization (WHO) and its member countries' health-related statistics. Specific information about the database can be found elsewhere [12]. Childhood ow/ob of 6 regions, 4 income levels, and 195 countries in 1975–2016 were extracted from the GHO database to project the prevalence of ow/ob among children aged 5–19 years in 2030. Among them, 4 countries were excluded due to missing data (Supplemental Table S1), and 191 countries had data on prevalence of ov/ob in all 42 waves of children from 1975–2016.

In addition, data on national economic indicators (gross national income per capita (GNI per capita) and urbanization rate) and social environment (higher education enrollment, CO_2_emissions and forest area) were extracted from the World Bank’s World Development Indicators database, which provided the latest and most accurate global development data, including data by countries and world regions [13]. There were 149 and 190 countries with two or more rounds of data on GNI and urbanization rate (90% countries had more than ten waves), and 186 countries had two or more rounds of data on CO_2_ emissions and forest area, but only 61 countries had two or more rounds of data on higher education enrollment (two rounds (34.4%), Three rounds (36.1%), > three rounds (29.5%)).

Food consumption indices (meat, vegetable and fruit) were extracted from the Food and Agriculture Organization of the United Nations, it included the supply sources and utilization of each type of food from 194 member countries, and 167 countries had two or more rounds of food consumption data (nearly 90% countries had more than ten waves) [14].

This analysis of data on the GHO and World Development Indicators was exempt from Institutional Review Board review.

### Definitions

Ov/ob were defined based on the WHO growth reference for school-aged children and adolescents [15]. It is worth noting that individual country’s prevalence in the GHO database was not always the same as official country estimates, as the Bayesian hierarchical model used in GHO database to ensure the comparability of data across time and countries. Regions were categorized according to the United Nations [16]. Income categories were divided by the World Bank [17].

### Statistical analysis

We used autoregressive integrated moving average (ARIMA) model and trend extrapolation method to project the prevalence of ow/ob among children in 191 countries in 2030 [18]. The ARIMA model was described in detail in related substantive papers [19]. Briefly, the basic idea of the ARIMA method is to use the linear combination of past and present values of time series to project future values. The ARIMA model fitting in this study included four steps (Text S1). The trend extrapolation method, which is a way to infer the future development of the research object according to its past and current time trend, was only used to project the data that did not meet the fitting conditions of ARIMA model. In this paper, we mainly used the linear model in trend extrapolation method. All the model parameters can be found in supplemental tables S2 and S3. We used the childhood ow/ob data from 1975 to 2010 for model fitting, and projected the data from 2011 to 2030, and then used the difference between the actual and projected values from 2011 to 2016 to calculate the relative error and the mean of relative error for model evaluation (Supplemental Tables S4 and S5). Projection analyses were performed by SAS 9.4 and Excel.

We employed the standard deviational ellipse (SDE), using ArcGIS 10.5, to analyze the spatial–temporal distribution of childhood ow/ob, GNI per capita and urbanization rate in the world from the perspective of the mean center and migrating traces, as well as the distribution range, density, direction, and shape. First, based on the spatial location (latitude and longitude) of 190 countries (Eswatini excluded from 191 countries), we used the country’s indicators (childhood ow/ob, GNI per capita and urbanization rate) to represent the weights, and calculated the weight mean center of each indicator in every year. Second, we marked the weight mean center of childhood ow/ob every five years from 1975 to 2030 on the map, and connected them in chronological order to reveal its spatial–temporal patterns over 50 years. The same method was used to label the spatial–temporal patterns of GNI per capita, and urbanization rate from 1975 to 2018. We used the Atlantic version of the world map in this study, different maps may result in different locations of the weight mean center. However, the spatial–temporal migration trend of the weight mean center may not be affected.

The scatter plots were used to illustrate the relationship between GNI per capita, urbanization rate, and childhood ow/ob. Based on the scatter plots, we found a nonlinear relationship between GNI per capita and childhood ow/ob, and a linear relationship between urbanization rate and childhood ow/ob. First, piecewise regression was applied to estimate the association between GNI per capita and childhood ow/ob in countries with different levels of GNI per capita. Second, multivariate adjusted coefficients (βs) and their 95% CIs of urbanization rate effects on childhood ow/ob were estimated by linear regression adjusting for education, environmental factors (CO_2_ emissions, forest area), meat consumption, vegetable, and fruit consumption. The analyses were performed using mixed-effects models. The variable of time (year) was modeled as a random effect in the model in Stata 14.0.

## Results

### Time trends and projection of childhood ow/ob prevalence

**Fig. 1:**
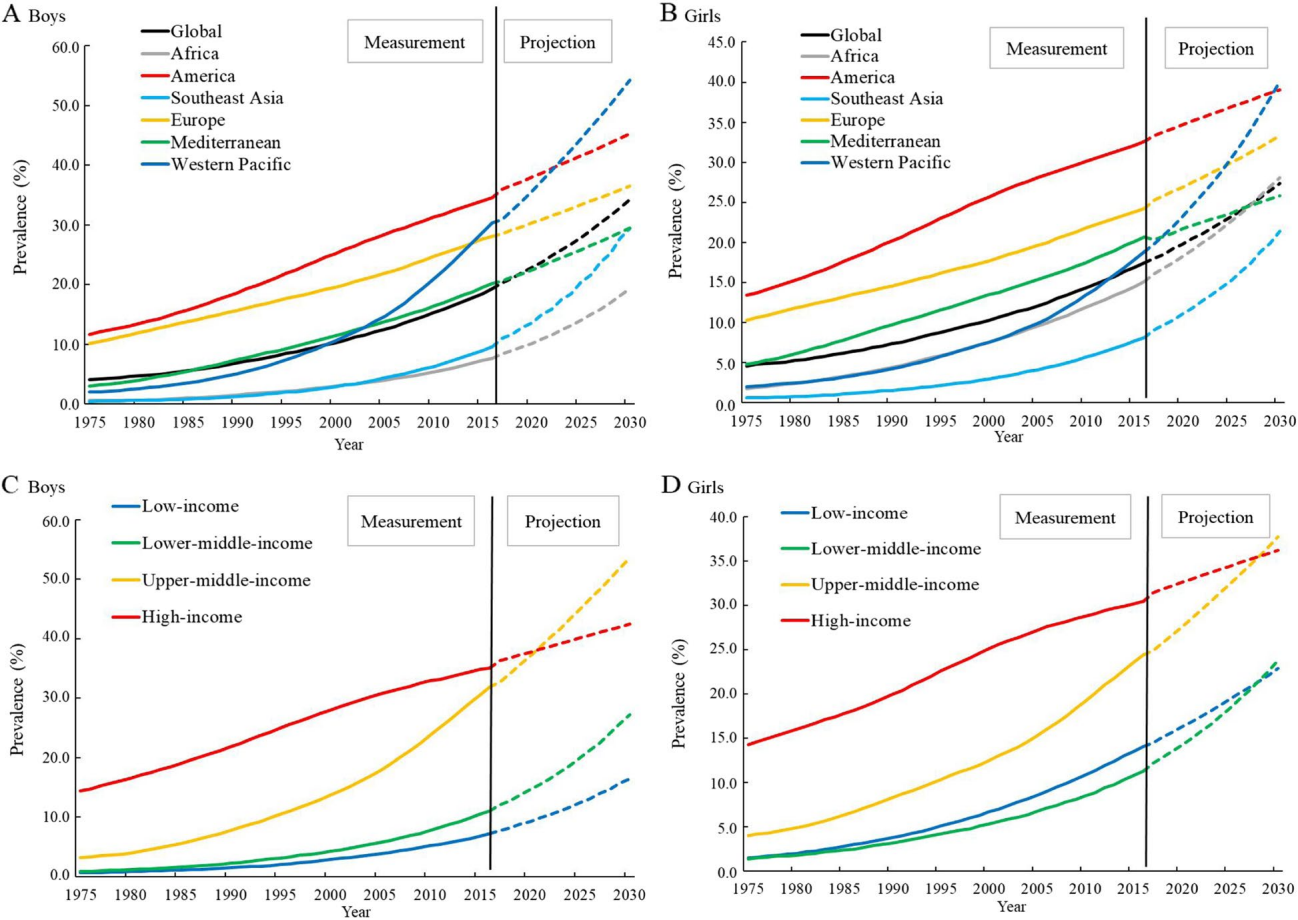
Time trends in prevalence of childhood overweight and obesity by regions and income level worldwide. Regions and income level are categorized according to the standards of the United Nations and the World Bank, respectively. Solid lines denote observed prevalence of childhood overweight and obesity, dotted lines denote projected prevalence of childhood overweight and obesity. **A** Time trends in prevalence (%) of childhood overweight and obesity by regions in boys. **B** Time trends in prevalence (%) of childhood overweight and obesity by regions in Girls. **C** Time trends in prevalence (%) of childhood overweight and obesity by income level in boys. **D** Time trends in prevalence (%) of childhood overweight and obesity by income level in Girls

Worldwide, the prevalence of ow/ob in boys increased faster than that of girls. The prevalence in boys increased from 4.1% (95%CI: 3.1%-5.3%) in 1975 to 19.3% (95%CI: 17.6%-21.1%) in 2016; and in girls it increased from 4.6% (95%CI: 3.6%- 5.9%) to 17.5% (95% CI: 16.1%-19.0%). The projected prevalence of ow/ob in 2030 would reached 34.2% (95% CI: 27.4%-42.5%) for boys and 27.4% (95% CI: 26.1%-28.7%) for girls. The burden of ow/ob among boys became increasingly high. Projective trends by country income levels and regional patterns illustrated that the prevalence and growth rate of ow/ob in both boys and girls were higher in high-income countries in Europe and America than in low- and middle-income countries in Africa, Southeast Asia and Western Pacific before 2000, but Africa, East Asia and Southeast Asia would have not only the highest childhood ow/ob rates but also the fastest growth rates after 2020. (Fig. 1 & Table S6).

**Fig. 2:**
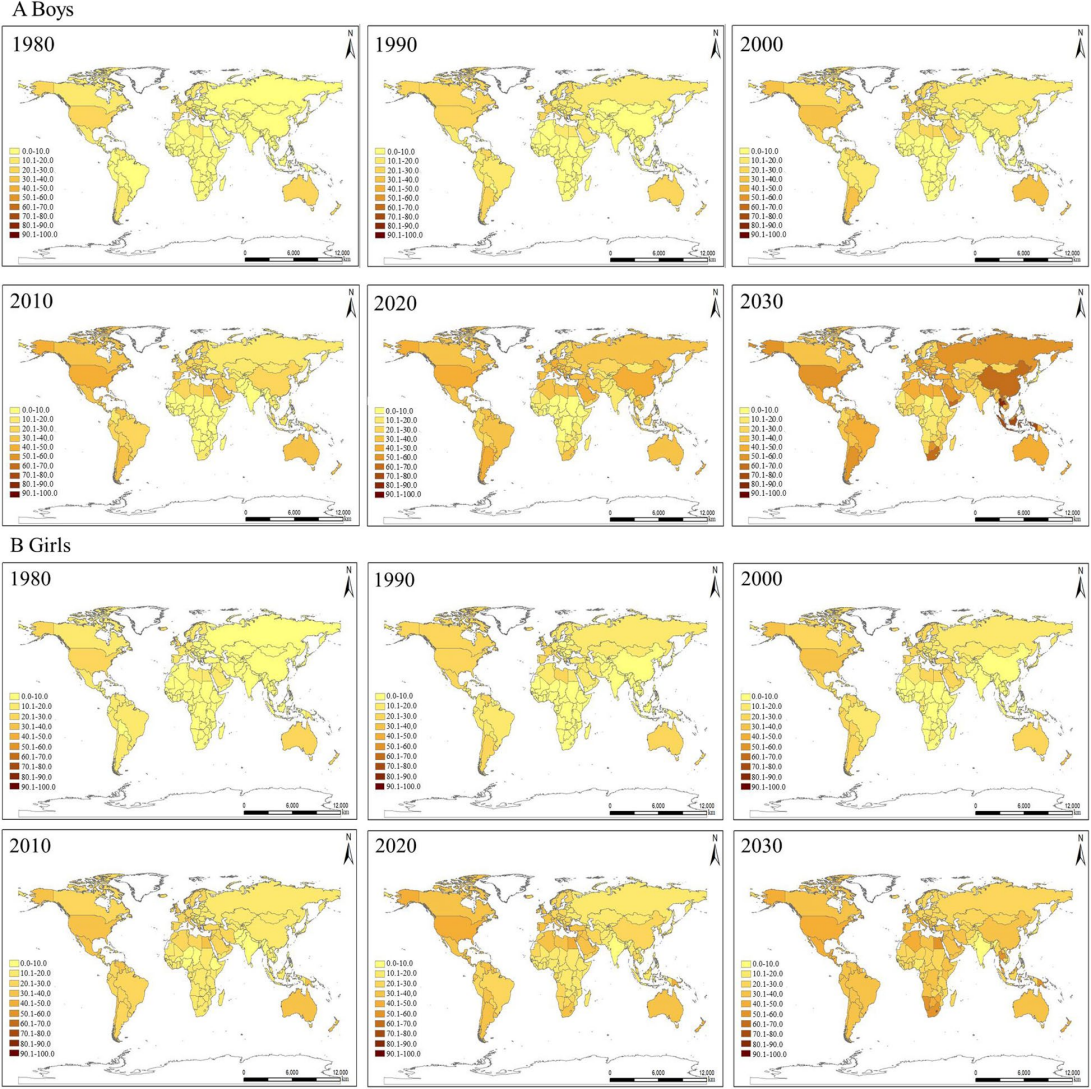
Observed and projected prevalence of overweight and obesity among boys and girls. 190 countries’ data were showed in world map. Monaco, San Marino, South Sudan and Sudan’ s childhood overweight and obesity prevalence were not projected because of missing data; Eswatini were not showed in world map

**Fig. 3:**
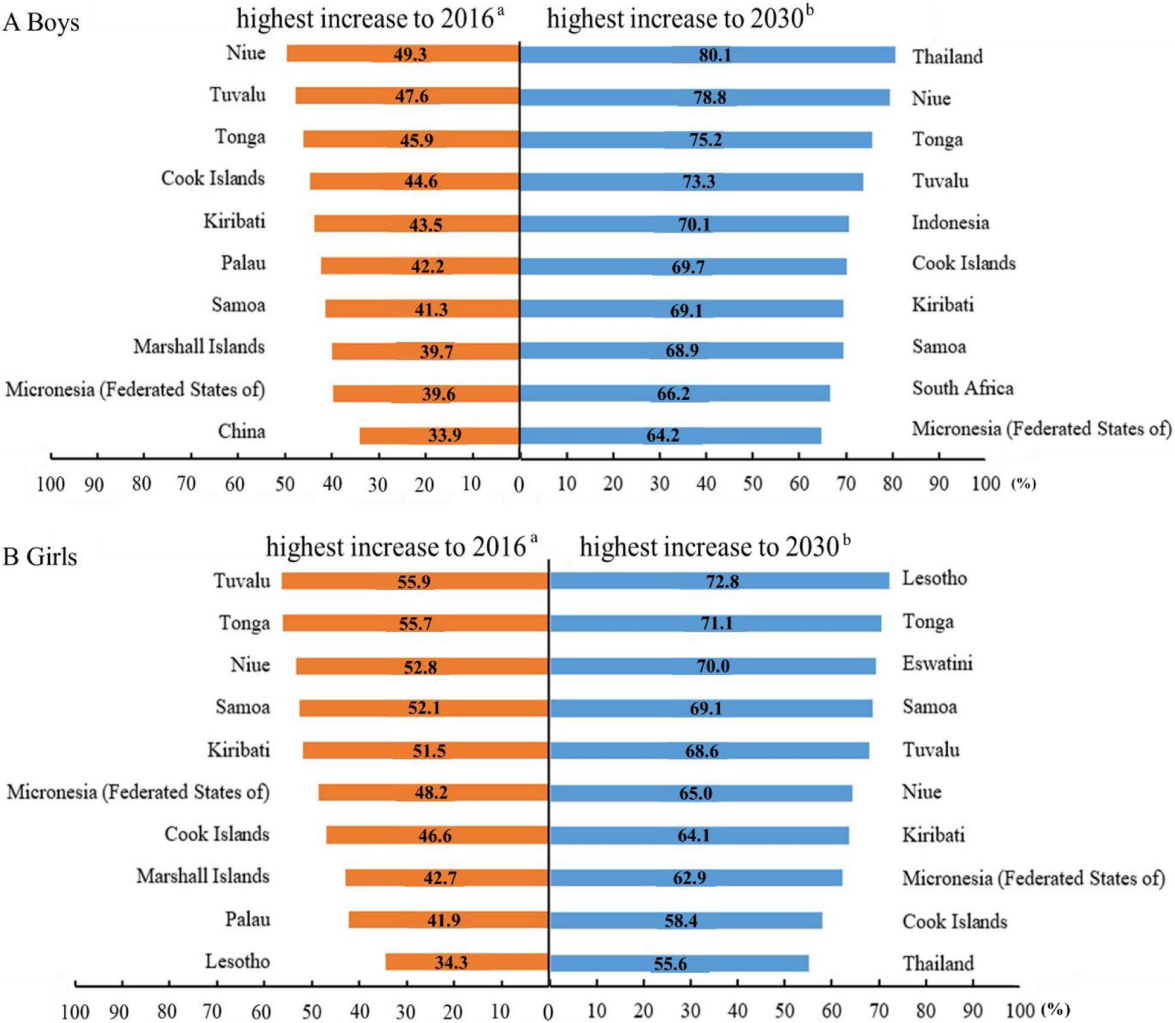
The top ten countries with the highest increase in prevalence in childhood overweight and obesity from 1975. ^a^ The prevalence in 2016 minus the prevalence in 1975. ^b^ The prevalence in 2030 minus the prevalence in 1975

By countries (Fig. 2 & Table S7), the prevalence of ow/ob among children in 1975 ranged from 0.3% (Bangladesh, Bhutan, India, Nepal) to 41.6% (Nauru), among which boys ranged from 0.1% (Burkina Faso) to 36.0% (Nauru) and girls from 0.3% (Nepal) to 47.6% (Nauru). Children with ow/ob were mainly aggregated in Europe, America and Australia, while the prevalence in African and Asian countries was low in 1975. The prevalence of ow/ob among children in 2015 ranged from 6.3% (India) to 64.4% (Nauru), which ranged from 4.4% (Uganda, Ethiopia) to 61.3% (Nauru) in boys and from 5.7% (India) to 67.8% (Nauru) in girls. According to the projection, the prevalence in 2030 ranged from 11.5% (Nicaragua) to 84.8% (Cook Islands) in all, 9.7% (Chad) to 85.6% (Cook Islands) in boys, and 10.8% (India) to 86.0% (Tonga) in girls. Table 1 and Fig. 3 showed the ten countries with the lowest and highest increase in childhood ow/ob from 1975 to 2016 and 1975 to 2030. It was found that most of the ten countries with the highest increase in 1975–2016 were island countries in the Pacific, such as Niue, Tuvalu. Meanwhile, the ten countries with the lowest increase can be divided into three categories: high-income countries (such as Singapore, Japan, et al.), low-income countries in the world (such as Nepal, Niger, et al.), and countries in the turbulent process of war (such as Ethiopia, et al.). Based on projection analysis, nine of the top ten countries with the least increase in 2030 are high-income countries (Singapore, Japan, Denmark, et al.).

**Table 1:** The top ten countries with the lowest increase in childhood overweight and obesity from 1975

	least increase to 2016^a^		least increase to 2030 ^b^	
	Country	Absolute growth	Country	Absolute growth
Boys	1 Singapore	4.3	1 Belgium	-1.5
	2 Ethiopia	4.5	2 Japan	4.5
	3 Uganda	4.5	3 Singapore	4.7
	4 Niger	4.9	4 Andorra	8.3
	5 Rwanda	5.0	5 Chad	9.5
	6 Burkina Faso	5.1	6 Denmark	9.6
	7 Guinea	5.3	7 Rwanda	9.7
	8 Senegal	5.3	8 Iceland	10.2
	9 Chad	5.3	9 Central African Republic	11.2
	10 Belgium	5.3	10 Sweden	11.7
Girls	1 Singapore	0.2	1 Belgium	-1.1
	2 Belgium	2.1	2 Singapore	0.0
	3 Japan	5.0	3 Denmark	2.9
	4 Denmark	6.2	4 Iceland	5.0
	5 India	6.7	5 Japan	5.6
	6 Iceland	6.8	6 Andorra	6.9
	7 Sweden	6.9	7 Sweden	7.1
	8 Viet Nam	8.6	8 Luxembourg	9.5
	9 Andorra	9.1	9 Switzerland	9.9
	10 Malta	9.3	10 India	10.4

^a^The prevalence in 2016 minus the prevalence in 1975

^b^The prevalence in 2030 minus the prevalence in 1975

### Spatial–temporal patterns of the global childhood ow/ob and economic indicators

**Fig. 4:**
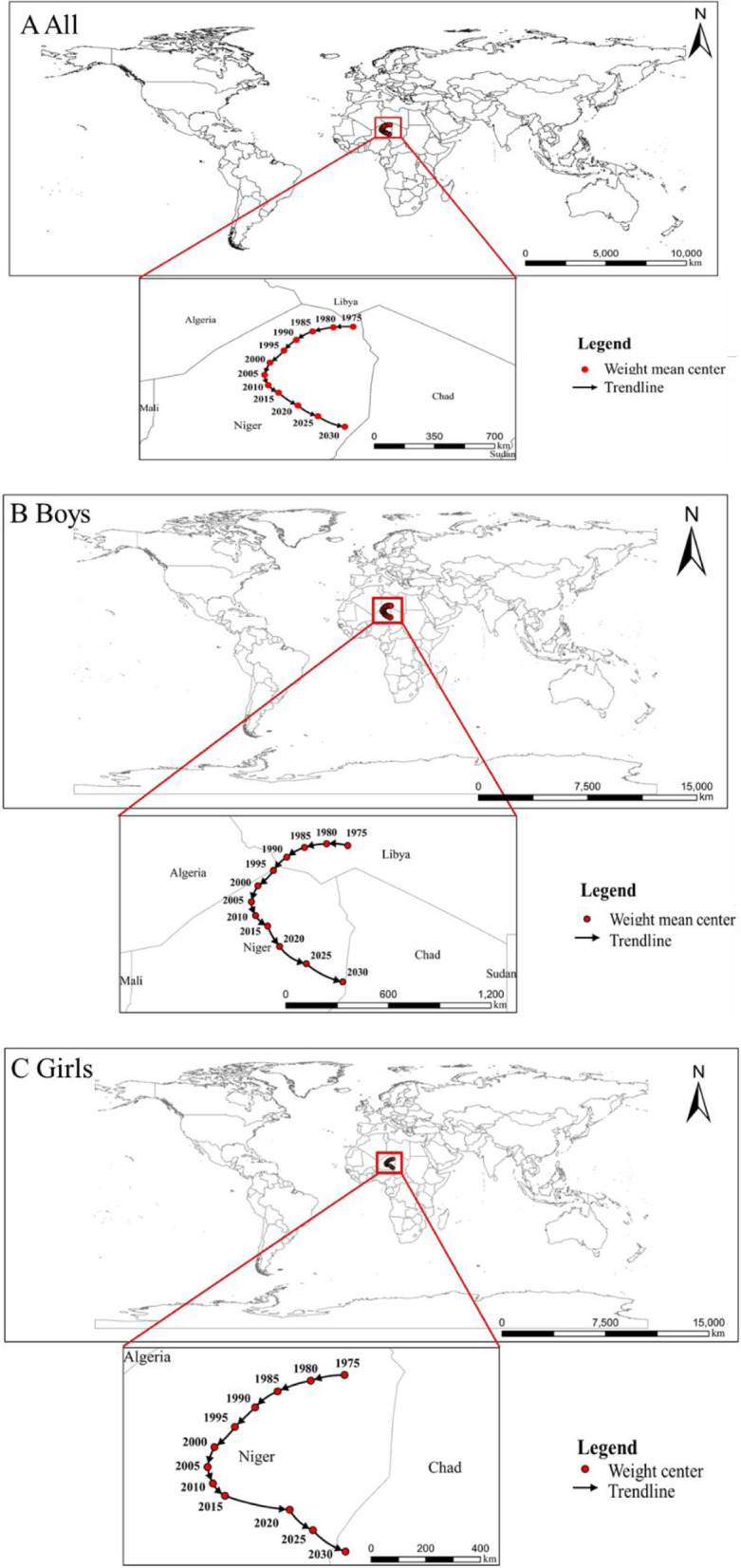
Spatial–temporal trends in global childhood overweight and obesity from 1975 to 2030. Standard deviational ellipse (SDE) method was used to analyze spatial–temporal distribution of childhood overweight and obesity. Weight mean center, the global weight mean center of overweight and obesity prevalence. **A** Spatial–temporal trends of all children’s prevalence of overweight and obesity from 1975 to 2030. **B** Spatial–temporal trends of boys’ prevalence of overweight and obesity from 1975 to 2030. **C** Spatial–temporal trends of girls’ prevalence of overweight and obesity from 1975 to 2030

Figure 4 showed the migrating trends of the weight mean center of global childhood ow/ob every five years from 1975 to 2030, a "C" shaped trend on the map. The turning time of the trends appeared since 2005. The weight mean center shifted from northeast to southwest before 2005 (1975 (15.6^。^E, 24.6^。^N) to 2005 (10.6^。^E, 21.7^。^N)), and then shifted from northwest to southeast from 2005 (10.6^。^E, 21.7^。^N) to 2030 (14.8^。^E, 17.4^。^N). The spatial–temporal trends of ow/ob for boys and girls were in general consistent with the overall trends. Such as from 1975 to 2005, boys’ ow/ob prevalence in Europe and America in the western part of the world increased by 11.8% and 16.7%, respectively, while the prevalence of ow/ob in Africa and Southeast Asia in eastern and southern part of the world increased by only 3.4% and 4.0% during the same period. However, from 2005 to 2030, the prevalence of ow/ob among boys in Africa and Southeast Asia increased faster than that in the past 30 years (2005 to 2030: 15.3% and 25.0%), and faster than that in Europe and America over the same period (2005 to 2030: 14.6% and 16.8%) (Table S6). The weight mean centers of boys were norther than those of girls, indicating that gender differences in the geographical distribution of childhood ow/ob existed, and boys in the north were heavier than their counterparts in the south. The latitude and longitude coordinates of the weight mean center, and the SDE’s coefficient about childhood ow/ob could be found in Table S8.

**Fig. 5:**
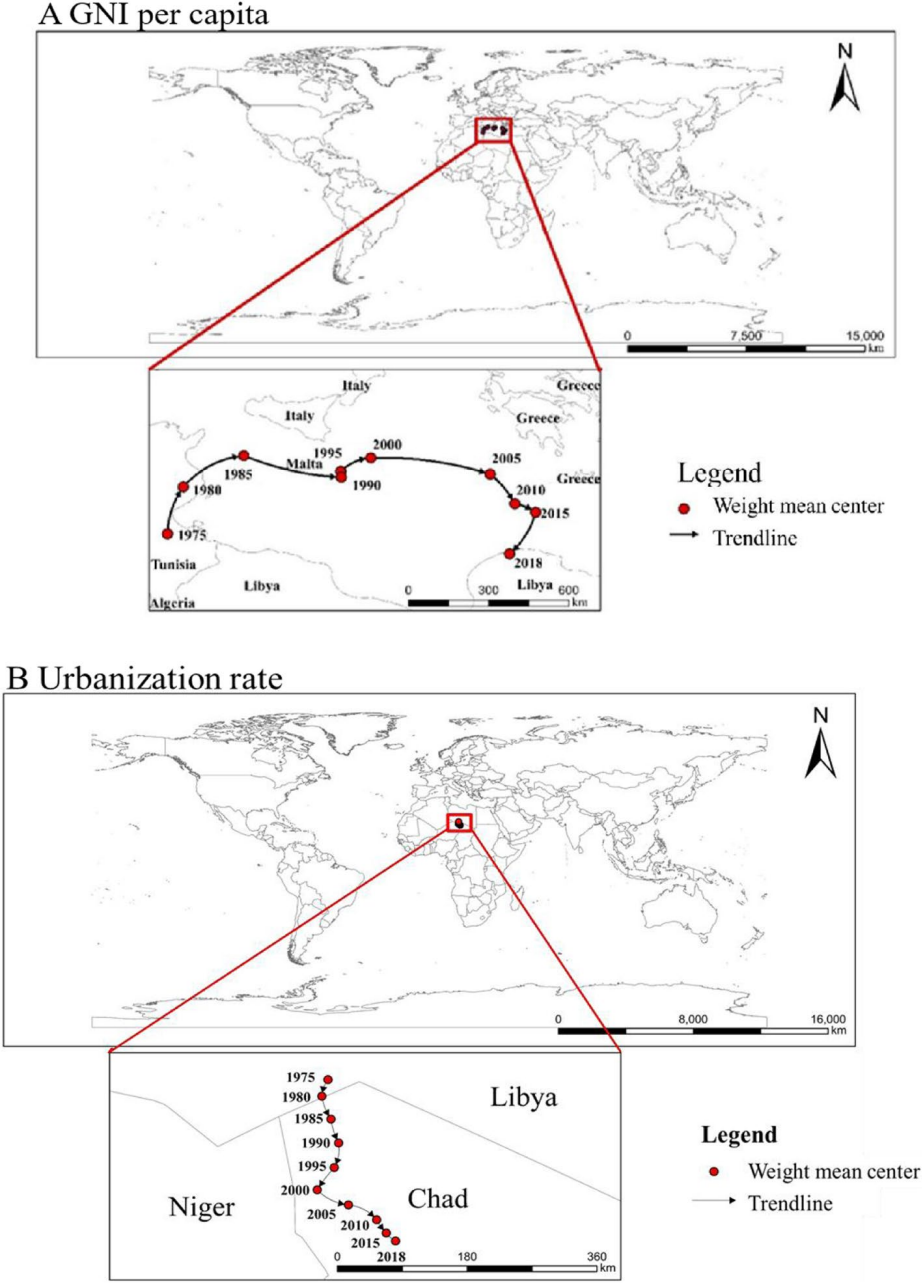
Spatial–temporal trends in GNI per capita and urbanization rate from 1975 to 2018. Standard deviational ellipse (SDE) method was used to analyze spatial–temporal distribution of GNI per capita and urbanization rate. **A** Spatial–temporal trends of GNI pre capita from 1975 to 2018 in 149 countries. **B** Spatial–temporal trends of urbanization rate from 1975 to 2018 in 190 countries. GNI per capita, gross national income per capita

The GNI per capita weight mean center was located close to the geographic weight mean center in the same region, which was at the junction of the African continent and the European continent in the current map. Compared to the weight mean center of childhood ow/ob, the GNI per capita's weight mean center was closer to the European continent in Fig. 5. The weight mean center of GNI per capita moved from west to east over time (1975 (9.8^。^E, 33.4^。^N) to 2018 (21.4^。^E, 32.7^。^N)), and stayed relatively stable in the north–south direction. However, from 2015 to 2018, it moved from north to south, and the trend of moving from west to east slowed down. The urbanization rate’s weight mean center was close to that of childhood ow/ob in Fig. 5. The moving trace of the weight mean center of urbanization rate was shown as an irregular "C" shape, from south to east from 1975 to 2000 (1975 (15.6^。^E, 23.5^。^N) to 2000 (15.5^。^E, 22.1^。^N)), and from northwest to southeast after 2000 to 2018 (16.5^。^E, 21.4^。^N)). The SDE’s coefficient (such as X standard deviation, Y standard deviation, Rotation) can be found in Table S9.

### Associations of the childhood ow/ob with economic indicators

Table 2 showed the adjusted βs and 95% CI for the associations between GNI per capita and childhood ow/ob prevalence. The mean of GNI per capita and $48,000 were used as the cutoff points, and piecewise regression was performed based on the scatter plots in Figure S1. When the country's GNI per capita was less than $48,000, for every $10,000 increasing in GNI per capita, the ow/ob rate increased by 3.8% (95% CI:2.2%, 5.4%) for boys and 2.4% (95% CI:1.0%, 3.7%) for girls. The association between GNI per capita and childhood ow/ob was not statistically significant when GNI per capita was above $48,000. In addition, in view of the possible lag in the impact of the national economy on childhood ow/ob, the 4-phase lag method was used to analyze the association between the national economy and childhood ow/ob, and similar results were shown in Table S10.

**Table 2:** Associations between GNI per capita and prevalence of childhood overweight and obesity using piecewise regression^a^

		Cutoff value 1						Cutoff value 2					
		< Mean			> = Mean			< 48,000			> = 48,000		
		β	95% CI	*P*	β	95% CI	*P*	β	95% CI	*P*	β	95% CI	*P*
Boys	GNI per capita	9.090	(5.220,12.954)	< 0.001	-0.765	(-1.746,0.216)	0.127	3.820	(2.232,5.404)	< 0.001	0.610	(-0.129,1.350)	0.106
	Higher education enrollment	0.062	(-0.151,0.275)	0.569	-0.050	(-0.196,0.095)	0.500	0.056	(-0.122,0.233)	0.538	-0.453	(-0.584,-0.321)	< 0.001
	CO_2_ Emissions	-0.205	(-0.433,0.022)	0.077	0.036	(-0.026,0.098)	0.257	-0.038	(-0.184,0.108)	0.613	0.148	(-0.041,0.337)	0.126
	Forest Area	0.003	(0.001,0.005)	0.014	-0.047	(-0.139,0.045)	0.318	0.002	(0.001,0.005)	0.035	-0.016	(-0.081,0.050)	0.635
	Meat consumption	15.800	(-5.527,37.126)	0.146	-4.390	(-12.101,3.313)	0.264	-3.590	(-19.697,12.523)	0.663	12.868	(-5.066,30.801)	0.160
	Vegetable and fruit consumption	1.860	(-2.328,6.053)	0.384	0.347	(-3.537,4.231)	0.861	1.500	(-1.761,4.769)	0.367	-1.920	(-24.036,-14.416)	< 0.001
Girls	GNI per capita	5.920	(2.887,8.951)	< 0.001	-0.123	(-1.11,0.864)	0.807	2.360	(1.008,3.715)	0.001	-0.263	(-0.984,0.458)	0.475
	Higher education enrollment	-0.027	(-0.151,0.097)	0.667	-0.384	(-0.568,-0.201)	< 0.001	-0.025	(-0.149,0.098)	0.688	-0.490	(-0.619,-0.362)	< 0.001
	CO_2_ Emissions	-0.059	(-0.233,0.115)	0.507	0.100	(0.022,0.178)	0.012	0.001	(-0.111,0.112)	0.997	0.150	(-0.034,0.334)	0.111
	Forest Area	0.001	(0.001,0.003)	0.062	-0.143	(-0.226,-0.060)	0.001	0.001	(-0.001,0.002)	0.586	-0.083	(-0.146,-0.019)	0.011
	Meat consumption	11.607	(-2.625,25.838)	0.110	-11.321	(-20.928,-1.714)	0.021	-0.986	(-13.777,11.805)	0.880	10.778	(-6.704,28.261)	0.227
	Vegetable and fruit consumption	0.232	(-2.430,2.894)	0.864	0.774	(-3.961,5.508)	0.749	0.478	(-2.121,3.078)	0.718	-1.770	(-22.411,-13.033)	< 0.001

^a^Piecewise analysis based on the inverted U-shaped relationship between GNI per capita and obesity. 58 countries were included in the model, and the remaining countries were excluded because each variable did not contain at least two observations. Cutoff values were chosen based on the mean of GNI per capita and the vertices of the inverted U-shaped relationship between GNI per capita and the prevalence of childhood overweight and obesity. Higher education enrollment (%), CO_2_ emissions (Ten million tons) and forest area (%) data were extracted from the World Bank, and used to evaluate the cultural level and social environment of the corresponding country. Meat consumption (Ten million tons) and vegetable and fruit consumption (Ten million tons) data were extracted from the Food and Agriculture Organization of the United Nations, and used to evaluate food consumption in the corresponding country. GNI per capita, gross national income per capita

Based on the scatter plots in Figure S2, Table 3 showed linear associations between urbanization rate and childhood ow/ob. For every 1.00% increase in the urbanization rate, childhood ow/ob rate increases by 0.28% (95% CI:0.20%, 0.37%) in boys, and 0.26% (95% CI:0.18%, 0.33%) in girls.

**Table 3:** The associations between urbanization rate and prevalence of childhood overweight and obesity

	Boys			Girls		
	β	95% CI	*P*	β	95% CI	*P*
Urbanization rate	0.283	(0.197,0.369)	< 0.001	0.255	(0.183,0.327)	< 0.001
Higher education enrollment	-0.084	(-0.250,0.083)	0.325	-0.182	(-0.297,-0.067)	0.002
CO_2_ Emissions	0.013	(-0.087,0.113)	0.800	0.023	(-0.053,0.098)	0.557
Forest Area	0.002	(-0.001,0.004)	0.138	0.001	(-6.228,23.343)	0.257
Meat consumption	1.710	(-10.528,13.948)	0.784	0.177	(-9.184,9.537)	0.970
Vegetable and fruit consumption	-1.490	(-3.721,0.742)	0.191	-0.727	(-2.554,1.100)	0.435

61 countries were included in the model, and the remaining countries were excluded because each variable did not contain at least two observations. Higher education enrollment (%), CO_2_ emissions (Ten million tons) and forest area (%) data were extracted from the World Bank, and used to evaluate the cultural level and social environment of the corresponding country. Meat consumption (Ten million tons) and vegetable and fruit consumption (Ten million tons) data were extracted from the Food and Agriculture Organization of the United Nations, and used to evaluate food consumption in the corresponding country

## Discussion

Using a GIS study approach (i.e., weight mean center), this study showed a “C” shape of the world's ow/ob burden migrating from Europe and America to Asia and Africa after 2005, which followed similar spatial–temporal patterns of global economic development. In addition, the prevalence of childhood ow/ob was projected to increase continuously, and increase faster in developing countries than developed countries from 2020 to 2030. The associations between economic indicators and childhood ow/ob indicated the importance of economic globalization in affecting increase of childhood ow/ob in the world, especially in developing countries.

To our knowledge, this is the first study that used global weight mean center, a novel GIS method, to describe the trends of spatial–temporal distribution of global childhood ow/ob from 1975 to 2030. It expanded the utilization of GIS in health science, and may have many potential useful applications in delivering health messages effectively. The novel utilization of GIS weight mean center has overcome the limitation of the traditional expression of obesity epidemic, which can only reflect one dimension – time trends or geographic distribution– in a single map. This analysis demonstrated the epidemic in two dimensions simultaneously– both time and space. Moreover, this study showed the importance of local-level factors in explaining geographic variation in obesity prevalence, thus holding implications for geographically targeted interventions to combat the ow/ob epidemic. Further, this study first projected the global and regional estimated prevalence of childhood ow/ob among school-aged children till 2030. This will help formulate tackling strategies against childhood ow/ob to achieve the United Nations Sustainable Development Goals.

The faster growth in childhood ow/ob in developing countries (low- and middle-income countries) reproted in this study was consistent with other studies on adult weight status. Adult’s BMI in South Asia, Southeast Asia, the Caribbean, and South Latin America had increased rapidly, but the growth rate in Europe was slower [20]. This study showed prevalence of ow/ob among children in Africa, the Western Pacific and Southeast Asia increased at an accelerated rate in the past decades. After 2020, both the prevalence and growth rate of childhood ow/ob in the Western Pacific were projected to exceed those in Europe and the North America. The phenomena might be related to the global economic development, cultural differences, and intergeneration effect of early life malnutrition. Previous results showed that as the economic developed, the prevalence of ow/ob in children gradually increased [21]. Since around 2000, Asian and African countries have experienced rapid economic development and significant social changes [13]. The following transitions in food consumption were closely associated with the growth in ow/ob among children and adolescents [22]. Cultural preference is another factor for body weight status. For example, some countries in Africa and the Pacific (such as Nauru and Samoa) had high prevalence of ow/ob, which may be associated to their preference for larger body types [23]. On the contrary, Japan's slow growth in ow/ob in the next ten years may be related to its social culture in favoring smaller body [24]. In addition, undernutrition and overnutrition coexist in developing countries undergoing rapid nutrition transition, women are vulnerable to the double burden of undernutrition and overnutrition, which has huge consequences not only for women themselves, but also for children [25], and animal experiment has shown that perinatal protein malnutrition has intergenerational effects on offspring [26].

The “C” shape and turning point of the geographic migrating trace in global childhood ow/ob may be related to the dual effects of economic development on childhood ow/ob [27]. We found that the positive association between low GNI per capita (< $48000) and childhood ow/ob, it was consistent with previous studies in adults [9]. However, the association between high GNI per capita and childhood ow/ob was not statistically significant. An important strength of the current study is that we identified the turning point of GNI per capita when the positive association disappeared. The breakpoint in the inverted "U"-shaped relationship between GNI per capita and childhood ow/ob in our study was between $40,000 and $50,000, a high-income level that is currently reached by fewer than 30 countries worldwide. Previous studies hardly could identify such turning points, since the income levels of countries included did not distribute in both sides of the potential cut-off income [28, 29].

The stages of ow/ob affected by economic development may be explained by the nutritiontransition theory [30], Western countries have entered a stage where the obesity no longer rises with socio-economic development, as economic development has resulted in healthier lifestyles and nutritional levels. Although the economies of Asian and African countries have developed to a certain extent, it is not enough to change people's unhealthy lifestyles, so they are still in the stage of rising obesity rates with economic development. In addition, the mechanism for the low childhood ow/ob increasing rate in developed countries may be also related to the strong commitment and leadership of their governments. For example, Denmark has controlled the increasing of childhood ow/ob since 2008, as the Danish government and the whole society have adopted many effective measures including improving diet and nutrition, promoting physical activity, strengthening ow/ob treatment, monitoring the society, and marketing environment, et al. [31]. The prevalence of ow/ob and related non-communicable diseases in Japan was at a low level, as the Japanese traditionally view diet and nutrition important affecting health and the future of their nation. Similarly, Singaporeans had one of the lowest prevalence of childhood ow/ob in the world, which may be partly explained by comprehensive policies and actions implemented by the Singapore's Ministry of Health and Health Promotion Committee, including good nutrition [32].

The similar spatial–temporal trend patterns between the urbanization rate and childhood ow/ob may be explained by urbanization-induced increases in nutrient levels, increased sedentary time, and decreased outdoor activities. Urbanization started in western countries due to the industrial revolution from around 1800 [33]. Since 2000, the world's urbanization rate has developed rapidly, and the rate reached 55% in 2018. Further, the increasing level of urbanization brought frequent food exchanges, and high-calorie ultra-processed food consumption. Such changes would affect the dietary structure and eating habits of children and adolescents, causing children to be overweight and obese [34]. Meanwhile, the acceleration of urbanization, especially the increasing number of electronic products such as mobile phones, TVs, and computers, has increased the sedentary behavior time among children, while outdoor activities have continued to decline, resulting in poor physical fitness [35]. Regarding the spatial–temporal patterns of both indicators, it should be noted that the turning point of urbanization rate was five years earlier than the point of childhood ow/ob. This highlighted the lagging effect of economic development on health outcomes.

This study has limitations. First, the GHO data cannot fully reflect the true prevalence of ow/ob among children in individual countries. To examine the magnitude of the potential bias, we compared some actual data of childhood ow/ob prevalence with the data we used in this study (Table S11). The data from both sources were very similar, with almost the same trends. Second, the confidence interval for the ow/ob prevalence became increasingly widen as the projection time increasing, although our projection model had excellent accuracy. This showed the limitation of our model to forecast on longer horizon of years. Third, it should be addressed that the current weight center is only prevalence-weighted, the weight center in Asia may be more influenced by some small pacific countries.

## Conclusions

The “C” curve shapped migrating trace of the weight mean center of childhood ow/ob indicated global ow/ob burden shifting from Europe and North America to Asia and Africa in the past two decades, which followed the shifting pattern of urbanization rate. Global childhood ow/ob prevalence would continue to increase till 2030, with faster rising rates in middle income countries and the Western Pacific region than other regions. Effective actions are needed to tackle the global childhood ow/ob epidemic and the disparities among countries and populations.

### Supplementary Information

**Additional file 1: Text S1.** The steps about the projection of childhood overweight and obesity prevalence with the ARIMA model. **Table S1.** The countries' name included in this study. **Table S2.** Projection model parameters of childhood overweight and obesity prevalence by regions and country income. **Table S3.** Projection model parameters of childhood overweight and obesity prevalence by countries. **Table S4.** Accuracy evaluation of projection models of children's obesity prevalence by regions and country income. **Table S5.** Accuracy evaluation of projection models of children's obesity prevalence by countries. **Table S6.** Observed and projected prevalence (95% CI) of overweight and obesity among children by regions and countries’ income from 1975 to 2030. **Table S7.** Observed and projected prevalence (95% CI) of overweight and obesity among children by countries from 1975 to 2030. **Table S8.** The SDE's coefficients of the weight mean center of global childhood overweight and obesity. **Table S9.** The SDE's coefficients of the weight mean center of GNI per capita and urbanization rate. **Table S10.** Lag analysis of the associations between GNI per capita and the prevalence of childhood overweight and obesity. **Table S11.** The prevalence of the childhood obesity based on the results of literature search. **Figure S1.** Scatterplot of GNI per capita and childhood overweight and obesity. Scatter plot of the relationship between GNI per capita and the prevalence of childhood overweight and obesity in 149 countries from 1975 to 2018. Black line is the scatter fitting curve based on quadratic equation. **Figure S2.** Scatterplot of urbanization rate and childhood overweight and obesity. Scatter plot of the relationship between urbanization rate and the prevalence of childhood overweight and obesity in 190 countries from 1975 to 2018. Black line is the scatter fitting curve based on linear equation.

## Data Availability

The datasets used during the current study are available from the corresponding author on reasonable request.

## Abbreviations

ARIMA: Autoregressive integrated moving average
BMI: Body mass index
GHO: Global Health Observatory
GIS: Geographic information science
GNI: Gross national income per capita
ow/ob: Overweight and obesity
SDE: Standard deviational ellipse
WHO: World Health Organization
