# Supplementary material for: Spatial–temporal trends in global childhood overweight and obesity from 1975 to 2030: a weight mean center and projection analysis of 191 countries
Source: Global Health. 2023 Aug 4;19:53. doi: 10.1186/s12992-023-00954-5 (PMC10403851; doi:10.1186/s12992-023-00954-5)
Supplement: Supplementary file 1 — Additional file 1: Text S1. The steps about the projection of childhood overweight and obesity prevalence with the ARIMA model. Table S1. The countries' name included in this study. Table S2. Projection model parameters of childhood overweight and obesity prevalence by regions and country income. Table S3. Projection model parameters of childhood overweight and obesity prevalence by countries. Table S4. Accuracy evaluation of projection models of children's obesity prevalence by regions and country income. Table S5. Accuracy evaluation of projection models of children's obesity prevalence by countries. Table S6. Observed and projected prevalence (95% CI) of overweight and obesity among children by regions and countries’ income from 1975 to 2030. Table S7. Observed and projected prevalence (95% CI) of overweight and obesity among children by countries from 1975 to 2030. Table S8. The SDE's coefficients of the weight mean center of global childhood overweight and obesity. Table S9. The SDE's coefficients of the weight mean center of GNI per capita and urbanization rate. Table S10. Lag analysis of the associations between GNI per capita and the prevalence of childhood overweight and obesity. Table S11. The prevalence of the childhood obesity based on the results of literature search. Figure S1. Scatterplot of GNI per capita and childhood overweight and obesity. Scatter plot of the relationship between GNI per capita and the prevalence of childhood overweight and obesity in 149 countries from 1975 to 2018. Black line is the scatter fitting curve based on quadratic equation. Figure S2. Scatterplot of urbanization rate and childhood overweight and obesity. Scatter plot of the relationship between urbanization rate and the prevalence of childhood overweight and obesity in 190 countries from 1975 to 2018. Black line is the scatter fitting curve based on linear equation. [file 12992_2023_954_MOESM1_ESM.docx]

**Supplemental materials**

**Text S1. The steps about the projection of childhood overweight and obesity prevalence with the ARIMA model**

**Table S1. The countries' name included in this study**

**Table S2. Projection model parameters of childhood overweight and obesity prevalence by regions and country income**

**Table S3. Projection model parameters of childhood overweight and obesity prevalence by countries**

**Table S4. Accuracy evaluation of projection models of children's obesity prevalence by regions and country income**

**Table S5. Accuracy evaluation of projection models of children's obesity prevalence by countries**

**Table S6. Observed and projected prevalence (95% CI) of overweight and obesity among children by regions and countries’ income from 1975 to 2030**

**Table S7. Observed and projected prevalence (95% CI) of overweight and obesity among children by countries from 1975 to 2030**

**Table S8. The SDE's coefficients of the weight mean center of global childhood overweight and obesity**

**Table S9. The SDE's coefficients of the weight mean center of GNI per capita and urbanization rate**

**Table S10. Lag analysis of the associations between GNI per capita and the prevalence of childhood overweight and obesity**

**Table S11. The prevalence of the childhood obesity based on the results of literature search**

**Figure S1. Scatterplot of GNI per capita and childhood overweight and obesity**

Scatter plot of the relationship between GNI per capita and the prevalence of childhood overweight and obesity in 149 countries from 1975 to 2018. Black line is the scatter fitting curve based on quadratic equation.

**Figure S2. Scatterplot of urbanization rate and childhood overweight and obesity**

Scatter plot of the relationship between urbanization rate and the prevalence of childhood overweight and obesity in 190 countries from 1975 to 2018. Black line is the scatter fitting curve based on linear equation.

**Text S1. The steps about the projection of childhood overweight and obesity prevalence with the ARIMA model**

Step 1, non-stationary time series were smoothed through log transformation and difference: drawing the measurement data sequence diagram, identifying the sequence characteristics, and using log transformation and difference to obtain a stationary random white noise sequence, non-stationary time series can be generally stabilized after the first or second order difference. In this study, after the log transformation, the data series after the difference of three times is still not stable, then the trend extrapolation method was used.

Step 2, model parameters were identified based on autocorrelation function (ACF) graph and the partial autocorrelation function (PACF) graph, SCAN model and Bayesian Information Criterion (BIC): using the ACF graph and the PACF graph to preliminarily determine the moving average order and autoregressive order, and selecting other coefficients base on the SCAN model and using BIC to identify the optimal model.

Step 3, the residual white noise of the ARIMA model was tested: except for the parameter estimation and model fitting test in step 2, we also perform white noise test on the model residuals. If the test fails, we need to perform model parameter estimation and fitting again.

Step 4, the projective effect of the model was evaluated: after the model passed the above tests, the difference between the projected data and the measured data was calculated to verify the evaluation of the model. If the model could not through the above steps, the trend extrapolation method was used to establish the project model.

**Table S1. The countries' name included in this study**

|  | Country name |  | Country name |  | Country name |
| --- | --- | --- | --- | --- | --- |
| 1 | Afghanistan | 71 | Guatemala | 141 | Republic of Moldova |
| 2 | Albania | 72 | Guinea | 142 | Republic of North Macedonia |
| 3 | Algeria | 73 | Guinea-Bissau | 143 | Romania |
| 4 | Andorra | 74 | Guyana | 144 | Russian Federation |
| 5 | Angola | 75 | Haiti | 145 | Rwanda |
| 6 | Antigua and Barbuda | 76 | Honduras | 146 | Saint Kitts and Nevis |
| 7 | Argentina | 77 | Hungary | 147 | Saint Lucia |
| 8 | Armenia | 78 | Iceland | 148 | Saint Vincent and the Grenadines |
| 9 | Australia | 79 | India | 149 | Samoa |
| 10 | Austria | 80 | Indonesia | 150 | San Marino ^*^ |
| 11 | Azerbaijan | 81 | Iran (Islamic Republic of) | 151 | Sao Tome and Principe |
| 12 | Bahamas | 82 | Iraq | 152 | Saudi Arabia |
| 13 | Bahrain | 83 | Ireland | 153 | Senegal |
| 14 | Bangladesh | 84 | Israel | 154 | Serbia |
| 15 | Barbados | 85 | Italy | 155 | Seychelles |
| 16 | Belarus | 86 | Jamaica | 156 | Sierra Leone |
| 17 | Belgium | 87 | Japan | 157 | Singapore |
| 18 | Belize | 88 | Jordan | 158 | Slovakia |
| 19 | Benin | 89 | Kazakhstan | 159 | Slovenia |
| 20 | Bhutan | 90 | Kenya | 160 | Solomon Islands |
| 21 | Bolivia (Plurinational State of) | 91 | Kiribati | 161 | Somalia |
| 22 | Bosnia and Herzegovina | 92 | Kuwait | 162 | South Africa |
| 23 | Botswana | 93 | Kyrgyzstan | 163 | South Sudan ^*^ |
| 24 | Brazil | 94 | Lao People's Democratic Republic | 164 | Spain |
| 25 | Brunei Darussalam | 95 | Latvia | 165 | Sri Lanka |
| 26 | Bulgaria | 96 | Lebanon | 166 | Sudan ^*^ |
| 27 | Burkina Faso | 97 | Lesotho | 167 | Sudan (former) |
| 28 | Burundi | 98 | Liberia | 168 | Suriname |
| 29 | Cabo Verde | 99 | Libya | 169 | Sweden |
| 30 | Cambodia | 100 | Lithuania | 170 | Switzerland |
| 31 | Cameroon | 101 | Luxembourg | 171 | Syrian Arab Republic |
| 32 | Canada | 102 | Madagascar | 172 | Tajikistan |
| 33 | Central African Republic | 103 | Malawi | 173 | Thailand |
| 34 | Chad | 104 | Malaysia | 174 | Timor-Leste |
| 35 | Chile | 105 | Maldives | 175 | Togo |
| 36 | China | 106 | Mali | 176 | Tonga |
| 37 | Colombia | 107 | Malta | 177 | Trinidad and Tobago |
| 38 | Comoros | 108 | Marshall Islands | 178 | Tunisia |
| 39 | Congo | 109 | Mauritania | 179 | Turkey |
| 40 | Cook Islands | 110 | Mauritius | 180 | Turkmenistan |
| 41 | Costa Rica | 111 | Mexico | 181 | Tuvalu |
| 42 | Cote d'Ivoire | 112 | Micronesia (Federated States of) | 182 | Uganda |
| 43 | Croatia | 113 | Monaco ^*^ | 183 | Ukraine |
| 44 | Cuba | 114 | Mongolia | 184 | United Arab Emirates |
| 45 | Cyprus | 115 | Montenegro | 185 | United Kingdom of Great Britain and Northern Ireland |
| 46 | Czechia | 116 | Morocco | 186 | United Republic of Tanzania |
| 47 | Democratic People's Republic of Korea | 117 | Mozambique | 187 | United States of America |
| 48 | Democratic Republic of the Congo | 118 | Myanmar | 188 | Uruguay |
| 49 | Denmark | 119 | Namibia | 189 | Uzbekistan |
| 50 | Djibouti | 120 | Nauru | 190 | Vanuatu |
| 51 | Dominica | 121 | Nepal | 191 | Venezuela (Bolivarian Republic of) |
| 52 | Dominican Republic | 122 | Netherlands | 192 | Viet Nam |
| 53 | Ecuador | 123 | New Zealand | 193 | Yemen |
| 54 | Egypt | 124 | Nicaragua | 194 | Zambia |
| 55 | El Salvador | 125 | Niger | 195 | Zimbabwe |
| 56 | Equatorial Guinea | 126 | Nigeria |  |  |
| 57 | Eritrea | 127 | Niue |  |  |
| 58 | Estonia | 128 | Norway |  |  |
| 59 | Eswatini | 129 | Oman |  |  |
| 60 | Ethiopia | 130 | Pakistan |  |  |
| 61 | Fiji | 131 | Palau |  |  |
| 62 | Finland | 132 | Panama |  |  |
| 63 | France | 133 | Papua New Guinea |  |  |
| 64 | Gabon | 134 | Paraguay |  |  |
| 65 | Gambia | 135 | Peru |  |  |
| 66 | Georgia | 136 | Philippines |  |  |
| 67 | Germany | 137 | Poland |  |  |
| 68 | Ghana | 138 | Portugal |  |  |
| 69 | Greece | 139 | Qatar |  |  |
| 70 | Grenada | 140 | Republic of Korea |  |  |

^*^Missing data. Four countries with missing data and Eswatini were not included in the study of weight center migration.

**Table S2. Projection model parameters of childhood overweight and obesity prevalence by regions and country income**

|  |  | ARIMA model | | | | | |  | Trend extrapolation model 's R^2 e^ |
| --- | --- | --- | --- | --- | --- | --- | --- | --- | --- |
|  |  | log transform ^a^ | Difference ^a^ | Unit root test ^b^ | white noise test ^b^ | parameter (*p, i, q*) ^c^ | residual white noise test ^d^ |  |  |
| Boys | Global | Yes | One | Pass | Pass | 2,1,1 | Pass |  | - |
|  | Africa | Yes | One | Pass | Pass | 0,1,1 | Pass |  | - |
|  | America | Yes | One | No pass | - | - | - |  | 0.9977 |
|  | Southeast Asia | Yes | Two | Pass | Pass | 0,1,1 | Pass |  | - |
|  | Europe | Yes | One | Pass | No pass | - | - |  | 0.9990 |
|  | Mediterranean | Yes | One | Pass | No pass | - | - |  | 0.9997 |
|  | Western Pacific | Yes | One | Pass | No pass | - | - |  | 0.9963 |
|  | Low-income | Yes | Two | Pass | Pass | 2,2,1 | Pass |  | - |
|  | Lower-middle-income | Yes | Three | Pass | Pass | 1,3,0 | Pass |  | - |
|  | Upper-middle-income | Yes | One | Pass | No pass | - | - |  | 0.9982 |
|  | High-income | Yes | One | Pass | No pass | - | - |  | 0.9970 |
|  |  |  |  |  |  |  |  |  |  |
| Girls | Global | Yes | One | Pass | Pass | 1,1,1 | Pass |  | - |
|  | Africa | Yes | One | Pass | Pass | 1,1,2 | Pass |  | - |
|  | America | Yes | One | Pass | No pass | - | - |  | 0.9979 |
|  | Southeast Asia | Yes | Two | Pass | Pass | 1,2,1 | Pass |  | - |
|  | Europe | Yes | One | Pass | Pass | 1,1,1 | Pass |  | - |
|  | Mediterranean | No | One | Pass | Pass | 1,1,1 | Pass |  | - |
|  | Western Pacific | Yes | One | Pass | Pass | 1,1,2 | Pass |  | - |
|  | Low-income | Yes | One | Pass | No pass | - | - |  | 0.9998 |
|  | Lower-middle-income | Yes | One | Pass | Pass | 0,1,0 | Pass |  | - |
|  | Upper-middle-income | Yes | One | Pass | No pass | - | - |  | 0.9980 |
|  | High-income | Yes | One | Pass | No pass | - | - |  | 0.9950 |

^a^ Smoothing non-stationary time series through log transformation and difference. ^b^ Checking whether the transformed sequence meets the stationarity requirements. ^c^ Identifying model parameters based on autocorrelation function (ACF) graph and the partial autocorrelation function (PACF) graph, SCAN model and Bayesian Information Criterion (BIC). ^d^ Residual error test after model establishment. ^e^ Data that cannot be modeled by ARIMA, and were used trend extrapolation to build the model.

**Table S3. Projection model parameters of childhood overweight and obesity prevalence by countries**

|  |  | ARIMA model | | | | | |  | Trend extrapolation model 's R^2 e^ |
| --- | --- | --- | --- | --- | --- | --- | --- | --- | --- |
|  |  | log transform ^a^ | Difference ^a^ | Unit root test ^b^ | white noise test ^b^ | parameters (*p, i, q*) ^c^ | residual white noise test ^d^ |  |  |
| Boys | Afghanistan | Yes | Two | Pass | Pass | 0,2,0 | Pass |  | - |
|  | Albania | Yes | One | Pass | Pass | 0,1,0 | Pass |  | - |
|  | Algeria | Yes | One | No pass | - | - | - |  | 0.9995 |
|  | Andorra | Yes | One | No pass | - | - | - |  | 0.9829 |
|  | Angola | Yes | One | Pass | No pass | - | - |  | 0.9922 |
|  | Antigua and Barbuda | Yes | One | No pass | - | - | - |  | 0.9996 |
|  | Argentina | Yes | One | No pass | - | - | - |  | 0.9999 |
|  | Armenia | NO | One | No pass | - | - | - |  | 0.9898 |
|  | Australia | Yes | Two | No pass | - | - | - |  | 0.9923 |
|  | Austria | Yes | One | No pass | - | - | - |  | 0.9964 |
|  | Azerbaijan | Yes | One | No pass | - | - | - |  | 0.997 |
|  | Bahamas | NO | One | No pass | - | - | - |  | 0.9988 |
|  | Bahrain | Yes | One | Pass | No pass | - | - |  | 0.9998 |
|  | Bangladesh | Yes | Two | Pass | Pass | 2,2,3 | Pass |  | - |
|  | Barbados | Yes | One | Pass | No pass | - | - |  | 0.999 |
|  | Belarus | NO | One | No pass | - | - | - |  | 0.9967 |
|  | Belgium | Yes | One | No pass | - | - | - |  | 0.9872 |
|  | Belize | Yes | One | No pass | - | - | - |  | 1 |
|  | Benin | Yes | Two | Pass | Pass | 1,2,2 | Pass |  | - |
|  | Bhutan | Yes | Three | Pass | Pass | 2,3,2 | Pass |  | - |
|  | Bolivia (Plurinational State of) | Yes | One | No pass | - | - | - |  | 0.9985 |
|  | Bosnia and Herzegovina | Yes | One | Pass | No pass | - | - |  | 0.9994 |
|  | Botswana | Yes | One | Pass | Pass | 0,1,0 | Pass |  | - |
|  | Brazil | Yes | One | Pass | No pass | - | - |  | 0.9998 |
|  | Brunei Darussalam | NO | One | Pass | No pass | - | - |  | 0.9995 |
|  | Bulgaria | Yes | One | No pass | - | - | - |  | 0.9983 |
|  | Burkina Faso | Yes | One | Pass | Pass | 1,1,1 | Pass |  | - |
|  | Burundi | Yes | One | Pass | Pass | 1,1,1 | Pass |  | - |
|  | Cabo Verde | Yes | One | Pass | Pass | 1,1,3 | Pass |  | - |
|  | Cambodia | Yes | One | Pass | Pass | 0,1,0 | Pass |  | - |
|  | Cameroon | Yes | One | Pass | No pass | - | - |  | 0.9995 |
|  | Canada | Yes | One | No pass | - | - | - |  | 0.9934 |
|  | Central African Republic | Yes | One | Pass | No pass | - | - |  | 0.9927 |
|  | Chad | Yes | One | Pass | No pass | - | - |  | 0.9948 |
|  | Chile | Yes | One | Pass | No pass | - | - |  | 0.9999 |
|  | China | Yes | One | Pass | Pass | 1,1,1 | Pass |  | - |
|  | Colombia | Yes | One | Pass | No pass | - | - |  | 0.9993 |
|  | Comoros | Yes | One | Pass | Pass | 1,1,0 | Pass |  | - |
|  | Congo | Yes | One | Pass | Pass | 1,1,0 | Pass |  | - |
|  | Cook Islands | Yes | Two | Pass | No pass | - | - |  | 0.997 |
|  | Costa Rica | Yes | One | No pass | - | - | - |  | 0.9989 |
|  | Cote d'Ivoire | Yes | One | Pass | Pass | 1,1,0 | Pass |  | - |
|  | Croatia | Yes | One | Pass | No pass | - | - |  | 0.9996 |
|  | Cuba | Yes | One | Pass | No pass | - | - |  | 0.9986 |
|  | Cyprus | NO | One | No pass | - | - | - |  | 0.9979 |
|  | Czechia | Yes | One | No pass | - | - | - |  | 0.9902 |
|  | Democratic People's Republic of Korea | Yes | One | Pass | No pass | - | - |  | 0.9995 |
|  | Democratic Republic of the Congo | Yes | Three | Pass | Pass | 0,3,0 | Pass |  | - |
|  | Denmark | Yes | One | Pass | No pass | - | - |  | 0.9979 |
|  | Djibouti | Yes | One | Pass | No pass | - | - |  | 0.9978 |
|  | Dominica | Yes | Two | Pass | No pass | - | - |  | 0.9967 |
|  | Dominican Republic | NO | One | Pass | No pass | - | - |  | 0.9996 |
|  | Ecuador | Yes | One | Pass | No pass | - | - |  | 0.9995 |
|  | Egypt | Yes | One | Pass | No pass | - | - |  | 0.9997 |
|  | El Salvador | Yes | One | No pass | - | - | - |  | 0.9971 |
|  | Equatorial Guinea | Yes | One | Pass | Pass | 1,1,1 | Pass |  | - |
|  | Eritrea | Yes | Three | Pass | Pass | 1,1,0 | Pass |  | - |
|  | Estonia | Yes | One | No pass | - | - | - |  | 0.9877 |
|  | Eswatini | Yes | Two | Pass | Pass | 0,1,1 | Pass |  | - |
|  | Ethiopia | Yes | Two | Pass | Pass | 2,2,2 | Pass |  | - |
|  | Fiji | Yes | One | Pass | No pass | - | - |  | 0.9989 |
|  | Finland | Yes | Two | Pass | No pass | - | - |  | 0.9967 |
|  | France | Yes | One | Pass | No pass | - | - |  | 0.9981 |
|  | Gabon | Yes | One | Pass | No pass | - | - |  | 0.9996 |
|  | Gambia | Yes | Two | Pass | Pass | 1,2,1 | Pass |  | - |
|  | Georgia | Yes | One | No pass | - | - | - |  | 0.9928 |
|  | Germany | Yes | Two | Pass | No pass | - | - |  | 0.9947 |
|  | Ghana | Yes | Two | Pass | Pass | 0,2,1 | Pass |  | - |
|  | Greece | NO | One | No pass | - | - | - |  | 0.9997 |
|  | Grenada | Yes | One | Pass | Pass | 1,1,1 | Pass |  | - |
|  | Guatemala | Yes | One | Pass | No pass | - | - |  | 0.9996 |
|  | Guinea | Yes | Three | Pass | Pass | 0,3,0 | Pass |  | - |
|  | Guinea-Bissau | Yes | Two | Pass | Pass | 1,2,3 | Pass |  | - |
|  | Guyana | Yes | One | Pass | Pass | 1,1,0 | Pass |  | - |
|  | Haiti | NO | One | Pass | No pass | - | - |  | 0.9996 |
|  | Honduras | Yes | One | Pass | No pass | - | - |  | 0.9998 |
|  | Hungary | Yes | One | No pass | - | - | - |  | 0.9967 |
|  | Iceland | Yes | One | No pass | - | - | - |  | 0.9981 |
|  | India | Yes | One | Pass | Pass | 0,1,1 | Pass |  | - |
|  | Indonesia | Yes | One | Pass | Pass | 1,1,0 | Pass |  | - |
|  | Iran (Islamic Republic of) | Yes | One | Pass | No pass | - | - |  | 0.9982 |
|  | Iraq | NO | One | No pass | - | - | - |  | 0.9996 |
|  | Ireland | Yes | One | Pass | No pass | - | - |  | 0.999 |
|  | Israel | Yes | One | No pass | - | - | - |  | 0.9996 |
|  | Italy | Yes | One | Pass | No pass | - | - |  | 0.9989 |
|  | Jamaica | Yes | One | Pass | No pass | - | - |  | 0.9999 |
|  | Japan | NO | One | No pass | - | - | - |  | 0.9967 |
|  | Jordan | Yes | One | No pass | - | - | - |  | 0.9949 |
|  | Kazakhstan | NO | One | No pass | - | - | - |  | 0.996 |
|  | Kenya | Yes | Two | Pass | Pass | 2,2,1 | Pass |  | - |
|  | Kiribati | Yes | One | Pass | No pass | - | - |  | 0.9983 |
|  | Kuwait | NO | One | No pass | - | - | - |  | 0.959 |
|  | Kyrgyzstan | Yes | One | Pass | Pass | 2,1,2 | Pass |  | - |
|  | Lao People's Democratic Republic | Yes | Two | Pass | Pass | 0,2,0 | Pass |  | - |
|  | Latvia | Yes | One | Pass | No pass | - | - |  | 0.9907 |
|  | Lebanon | Yes | One | No pass | - | - | - |  | 0.9997 |
|  | Lesotho | Yes | One | Pass | Pass | 2,1,2 | Pass |  | - |
|  | Liberia | Yes | One | Pass | Pass | 1,1,1 | Pass |  | - |
|  | Libya | NO | One | No pass | - | - | - |  | 0.9966 |
|  | Lithuania | Yes | One | Pass | No pass | - | - |  | 0.9944 |
|  | Luxembourg | Yes | One | Pass | No pass | - | - |  | 0.9987 |
|  | Madagascar | Yes | Two | Pass | Pass | 2,2,2 | Pass |  | - |
|  | Malawi | Yes | Two | Pass | Pass | 2,2,2 | Pass |  | - |
|  | Malaysia | Yes | One | Pass | No pass | - | - |  | 0.9991 |
|  | Maldives | Yes | Two | Pass | No pass | - | - |  | 0.9899 |
|  | Mali | Yes | Three | Pass | Pass | 1,3,1 | Pass |  | - |
|  | Malta | Yes | One | No pass | - | - | - |  | 0.9979 |
|  | Marshall Islands | Yes | One | Pass | No pass | - | - |  | 0.9991 |
|  | Mauritania | Yes | One | Pass | No pass | - | - |  | 0.9999 |
|  | Mauritius | Yes | One | Pass | Pass | 0,1,0 | Pass |  | - |
|  | Mexico | Yes | One | Pass | No pass | - | - |  | 0.9989 |
|  | Micronesia (Federated States of) | NO | One | Pass | No pass | - | - |  | 0.9999 |
|  | Monaco | NO data | - | - | - | - | - |  | - |
|  | Mongolia | Yes | One | Pass | No pass | - | - |  | 0.9932 |
|  | Montenegro | Yes | One | Pass | No pass | - | - |  | 0.9987 |
|  | Morocco | Yes | One | Pass | No pass | - | - |  | 0.9994 |
|  | Mozambique | Yes | One | Pass | Pass | 0,1,0 | Pass |  | - |
|  | Myanmar | Yes | Two | Pass | Pass | 1,2,1 | Pass |  | - |
|  | Namibia | Yes | Two | Pass | Pass | 1,2,1 | Pass |  | - |
|  | Nauru | Yes | One | No pass | - | - | - |  | 0.9996 |
|  | Nepal | Yes | One | Pass | No pass | - | - |  | 0.9972 |
|  | Netherlands | Yes | One | No pass | - | - | - |  | 0.9949 |
|  | New Zealand | Yes | Two | No pass | - | - | - |  | 0.9957 |
|  | Nicaragua | Yes | One | Pass | No pass | - | - |  | 0.9998 |
|  | Niger | Yes | Three | Pass | Pass | 0,1,0 | Pass |  | - |
|  | Nigeria | Yes | Three | Pass | Pass | 0,3,0 | Pass |  | - |
|  | Niue | Yes | One | Pass | No pass | - | - |  | 0.9996 |
|  | Norway | Yes | One | Pass | No pass | - | - |  | 0.9976 |
|  | Oman | Yes | One | Pass | No pass | - | - |  | 0.9956 |
|  | Pakistan | Yes | Two | Pass | Pass | 2,1,1 | Pass |  | - |
|  | Palau | Yes | One | Pass | No pass | - | - |  | 0.9991 |
|  | Panama | Yes | One | Pass | No pass | - | - |  | 0.9973 |
|  | Papua New Guinea | Yes | One | Pass | No pass | - | - |  | 0.9999 |
|  | Paraguay | Yes | One | Pass | No pass | - | - |  | 0.9995 |
|  | Peru | Yes | One | Pass | No pass | - | - |  | 1 |
|  | Philippines | Yes | One | Pass | No pass | - | - |  | 0.9991 |
|  | Poland | Yes | One | No pass | - | - | - |  | 0.9966 |
|  | Portugal | Yes | One | No pass | - | - | - |  | 0.9923 |
|  | Qatar | Yes | One | Pass | No pass | - | - |  | 0.9956 |
|  | Republic of Korea | Yes | One | No pass | - | - | - |  | 0.998 |
|  | Republic of Moldova | Yes | One | Pass | No pass | - | - |  | 0.9948 |
|  | Republic of North Macedonia | Yes | One | Pass | No pass | - | - |  | 0.999 |
|  | Romania | Yes | One | Pass | No pass | - | - |  | 0.9966 |
|  | Russian Federation | Yes | Two | No pass | - | - | - |  | 0.9946 |
|  | Rwanda | Yes | One | Pass | No pass | - | - |  | 0.9983 |
|  | Saint Kitts and Nevis | Yes | One | Pass | Pass | 1,1,0 | Pass |  | - |
|  | Saint Lucia | Yes | One | Pass | No pass | - | - |  | 0.9988 |
|  | Saint Vincent and the Grenadines | Yes | One | Pass | No pass | - | - |  | 0.9999 |
|  | Samoa | NO | One | No pass | - | - | - |  | 0.9994 |
|  | San Marino | NO data | - | - | - | - | - |  | - |
|  | Sao Tome and Principe | Yes | Two | Pass | Pass | 3,2,2 | Pass |  | - |
|  | Saudi Arabia | Yes | Two | Pass | No pass | - | - |  | 0.9996 |
|  | Senegal | Yes | Two | Pass | Pass | 1,2,1 | Pass |  | - |
|  | Serbia | Yes | One | Pass | No pass | - | - |  | 0.9999 |
|  | Seychelles | Yes | One | Pass | No pass | - | - |  | 0.9997 |
|  | Sierra Leone | Yes | Two | Pass | Pass | 1,2,2 | Pass |  | - |
|  | Singapore | Yes | Two | Pass | No pass | - | - |  | 0.9812 |
|  | Slovakia | Yes | One | No pass | - | - | - |  | 0.9956 |
|  | Slovenia | Yes | One | Pass | No pass | - | - |  | 0.9976 |
|  | Solomon Islands | Yes | One | Pass | No pass | - | - |  | 0.9997 |
|  | Somalia | Yes | Two | Pass | Pass | 0,1,1 | Pass |  | - |
|  | South Africa | Yes | One | Pass | No pass | - | - |  | 0.9991 |
|  | South Sudan | NO data | - | - | - | - | - |  | - |
|  | Spain | NO | One | Pass | No pass | - | - |  | 0.9998 |
|  | Sri Lanka | Yes | Two | Pass | Pass | 3,2,2 | Pass |  | - |
|  | Sudan | NO data | - | - | - | - | - |  | - |
|  | Sudan (former) | Yes | One | No pass | - | - | - |  | 0.9994 |
|  | Suriname | NO | One | No pass | - | - | - |  | 0.9982 |
|  | Sweden | NO | One | No pass | - | - | - |  | 0.9951 |
|  | Switzerland | Yes | Two | Pass | No pass | - | - |  | 0.9786 |
|  | Syrian Arab Republic | Yes | One | Pass | No pass | - | - |  | 0.9998 |
|  | Tajikistan | Yes | One | Pass | Pass | 0,1,0 | Pass |  | - |
|  | Thailand | Yes | One | Pass | Pass | 1,1,2 | Pass |  | - |
|  | Timor-Leste | Yes | Two | Pass | Pass | 2,2,2 | Pass |  | - |
|  | Togo | Yes | One | Pass | Pass | 1,1,1 | Pass |  | - |
|  | Tonga | Yes | One | No pass | - | - | - |  | 0.999 |
|  | Trinidad and Tobago | Yes | One | Pass | Pass | 2,1,1 | Pass |  | - |
|  | Tunisia | Yes | One | Pass | No pass | - | - |  | 0.9998 |
|  | Turkey | Yes | Two | Pass | No pass | - | - |  | 0.9984 |
|  | Turkmenistan | Yes | One | Pass | No pass | - | - |  | 0.999 |
|  | Tuvalu | Yes | One | No pass | - | - | - |  | 0.9984 |
|  | Uganda | Yes | Three | Pass | Pass | 1,3,2 | Pass |  | - |
|  | Ukraine | Yes | One | No pass | - | - | - |  | 0.9939 |
|  | United Arab Emirates | Yes | One | Pass | No pass | - | - |  | 0.9923 |
|  | United Kingdom of Great Britain and Northern Ireland | Yes | One | No pass | - | - | - |  | 0.9901 |
|  | United Republic of Tanzania | Yes | Two | Pass | Pass | 1,2,3 | Pass |  | - |
|  | United States of America | Yes | One | No pass | - | - | - |  | 0.9928 |
|  | Uruguay | Yes | One | Pass | Pass | 1,1,2 | Pass |  | - |
|  | Uzbekistan | Yes | One | Pass | Pass | 2,1,2 | Pass |  | - |
|  | Vanuatu | Yes | One | Pass | No pass | - | - |  | 0.9999 |
|  | Venezuela (Bolivarian Republic of) | Yes | One | No pass | - | - | - |  | 0.9995 |
|  | Viet Nam | Yes | One | Pass | Pass | 1,1,1 | Pass |  | - |
|  | Yemen | Yes | One | Pass | Pass | 0,1,1 | Pass |  | - |
|  | Zambia | Yes | One | Pass | Pass | 0,1,2 | Pass |  | - |
|  | Zimbabwe | Yes | Three | Pass | Pass | 0,3,1 | Pass |  | - |
|  |  |  |  |  |  |  |  |  |  |
| Girls | Afghanistan | Yes | Three | Pass | Pass | 1,3,0 | Pass |  | - |
|  | Albania | Yes | One | Pass | Pass | 0,1,1 | Pass |  | - |
|  | Algeria | Yes | One | No pass | - | - | - |  | 0.9984 |
|  | Andorra | Yes | One | No pass | - | - | - |  | 0.9698 |
|  | Angola | Yes | One | Pass | Pass | 1,1,1 | Pass |  | - |
|  | Antigua and Barbuda | Yes | One | No pass | - | - | - |  | 0.9996 |
|  | Argentina | No | One | Pass | No pass | - | - |  | 0.9993 |
|  | Armenia | No | One | Pass | Pass | 1,1,1 | No pass |  | 0.9923 |
|  | Australia | Yes | One | No pass | - | - | - |  | 0.9911 |
|  | Austria | No | One | Pass | Pass | 1,1,1 | No pass |  | 0.9984 |
|  | Azerbaijan | No | One | Pass | Pass | 0,1,0 | Pass |  | - |
|  | Bahamas | Yes | One | Pass | No pass | - | - |  | 0.9987 |
|  | Bahrain | No | One | Pass | Pass | 1,1,1 | Pass |  | - |
|  | Bangladesh | Yes | One | Pass | Pass | 1,1,0 | Pass |  | - |
|  | Barbados | Yes | One | Pass | No pass | - | - |  | 0.9991 |
|  | Belarus | No | One | Pass | No pass | - | - |  | 0.9979 |
|  | Belgium | Yes | One | Pass | No pass | - | - |  | 0.9851 |
|  | Belize | Yes | One | Pass | No pass | - | - |  | 0.9997 |
|  | Benin | Yes | One | Pass | No pass | - | - |  | 0.9999 |
|  | Bhutan | Yes | Two | Pass | Pass | 1,2,1 | Pass |  | - |
|  | Bolivia (Plurinational State of) | No | One | Pass | No pass | - | - |  | 0.9981 |
|  | Bosnia and Herzegovina | Yes | One | Pass | No pass | - | - |  | 0.9989 |
|  | Botswana | Yes | One | No pass | - | - | - |  | 0.9987 |
|  | Brazil | No | One | Pass | Pass | 0,1,0 | Pass |  | - |
|  | Brunei Darussalam | No | One | Pass | Pass | 1,1,2 | Pass |  | - |
|  | Bulgaria | No | One | Pass | No pass | - | - |  | 0.9984 |
|  | Burkina Faso | Yes | One | Pass | Pass | 1,1,0 | Pass |  | - |
|  | Burundi | Yes | One | Pass | Pass | 1,1,2 | Pass |  | - |
|  | Cabo Verde | Yes | One | No pass | - | - | - |  | 0.9947 |
|  | Cambodia | Yes | One | Pass | Pass | 1,1,0 | Pass |  | - |
|  | Cameroon | Yes | One | Pass | No pass | - | - |  | 0.9987 |
|  | Canada | Yes | One | No pass | - | - | - |  | 0.9948 |
|  | Central African Republic | Yes | One | Pass | Pass | 1,1,3 | Pass |  | - |
|  | Chad | Yes | One | Pass | Pass | 1,1,2 | Pass |  | - |
|  | Chile | No | One | Pass | Pass | 1,1,0 | Pass |  | - |
|  | China | Yes | One | Pass | Pass | 2,1,2 | Pass |  | - |
|  | Colombia | No | One | Pass | Pass | 1,1,2 | Pass |  | - |
|  | Comoros | Yes | One | Pass | No pass | - | - |  | 0.9998 |
|  | Congo | Yes | One | Pass | No pass | - | - |  | 0.9999 |
|  | Cook Islands | Yes | One | No pass | - | - | - |  | 0.9969 |
|  | Costa Rica | Yes | One | No pass | - | - | - |  | 0.9981 |
|  | Cote d'Ivoire | Yes | One | Pass | No pass | - | - |  | 0.9997 |
|  | Croatia | Yes | One | Pass | No pass | - | - |  | 0.9992 |
|  | Cuba | No | One | Pass | No pass | - | - |  | 0.9987 |
|  | Cyprus | No | One | No pass | - | - | - |  | 0.998 |
|  | Czechia | No | One | No pass | - | - | - |  | 0.9876 |
|  | Democratic People's Republic of Korea | Yes | One | Pass | No pass | - | - |  | 0.9992 |
|  | Democratic Republic of the Congo | Yes | One | Pass | Pass | 1,1,0 | Pass |  | - |
|  | Denmark | Yes | One | Pass | No pass | - | - |  | 0.9967 |
|  | Djibouti | No | One | Pass | No pass | - | - |  | 0.9987 |
|  | Dominica | Yes | Two | Pass | No pass | - | - |  | 0.996 |
|  | Dominican Republic | No | One | Pass | No pass | - | - |  | 0.9996 |
|  | Ecuador | No | One | Pass | No pass | - | - |  | 0.9994 |
|  | Egypt | Yes | One | No pass | - | - | - |  | 0.9996 |
|  | El Salvador | Yes | One | No pass | - | - | - |  | 0.9965 |
|  | Equatorial Guinea | Yes | One | No pass | - | - | - |  | 0.9991 |
|  | Eritrea | Yes | One | Pass | No pass | - | - |  | 0.9996 |
|  | Estonia | No | One | Pass | No pass | - | - |  | 0.9893 |
|  | Eswatini | Yes | One | Pass | Pass | 1,1,0 | Pass |  | - |
|  | Ethiopia | Yes | One | Pass | Pass | 0,1,0 | Pass |  | - |
|  | Fiji | No | One | No pass | - | - | - |  | 0.9975 |
|  | Finland | No | One | No pass | - | - | - |  | 0.997 |
|  | France | No | One | Pass | Pass | 2,1,2 | Pass |  | - |
|  | Gabon | No | One | Pass | No pass | - | - |  | 0.9987 |
|  | Gambia | Yes | One | Pass | No pass | - | - |  | 0.9982 |
|  | Georgia | No | One | No pass | - | - | - |  | 0.9948 |
|  | Germany | Yes | One | Pass | No pass | - | - |  | 0.9962 |
|  | Ghana | Yes | One | Pass | No pass | - | - |  | 0.9988 |
|  | Greece | No | One | No pass | - | - | - |  | 0.9994 |
|  | Grenada | Yes | One | Pass | Pass | 1,1,1 | Pass |  | - |
|  | Guatemala | Yes | One | No pass | - | - | - |  | 0.9991 |
|  | Guinea | Yes | One | Pass | No pass | - | - |  | 0.9999 |
|  | Guinea-Bissau | Yes | One | Pass | Pass | 1,1,1 | Pass |  | - |
|  | Guyana | No | One | Pass | Pass | 0,1,0 | Pass |  | - |
|  | Haiti | Yes | One | Pass | No pass | - | - |  | 0.9995 |
|  | Honduras | Yes | One | Pass | No pass | - | - |  | 0.9993 |
|  | Hungary | No | One | Pass | Pass | 1,1,2 | Pass |  | - |
|  | Iceland | Yes | One | Pass | No pass | - | - |  | 0.9978 |
|  | India | Yes | One | Pass | No pass | - | - |  | 0.9934 |
|  | Indonesia | Yes | One | Pass | No pass | - | - |  | 0.9998 |
|  | Iran (Islamic Republic of) | Yes | One | No pass | - | - | - |  | 0.9956 |
|  | Iraq | No | One | Pass | No pass | - | - |  | 0.9991 |
|  | Ireland | Yes | One | Pass | No pass | - | - |  | 0.9983 |
|  | Israel | Yes | One | Pass | Pass | 1,1,1 | Pass |  | - |
|  | Italy | Yes | One | Pass | No pass | - | - |  | 0.997 |
|  | Jamaica | Yes | One | Pass | No pass | - | - |  | 0.9997 |
|  | Japan | No | One | Pass | Pass | 1,1,1 | Pass |  | - |
|  | Jordan | Yes | One | No pass | - | - | - |  | 0.993 |
|  | Kazakhstan | No | One | Pass | Pass | 2,1,2 | Pass |  | - |
|  | Kenya | Yes | One | Pass | Pass | 1,1,2 | Pass |  | - |
|  | Kiribati | No | One | No pass | - | - | - |  | 0.9981 |
|  | Kuwait | Yes | One | No pass | - | - | - |  | 0.9973 |
|  | Kyrgyzstan | No | One | Pass | Pass | 1,1,1 | Pass |  | - |
|  | Lao People's Democratic Republic | Yes | Three | Pass | Pass | 0,3,2 | Pass |  | - |
|  | Latvia | No | One | Pass | Pass | 1,1,0 | Pass |  | - |
|  | Lebanon | No | One | Pass | No pass | - | - |  | 0.9993 |
|  | Lesotho | Yes | One | Pass | Pass | 1,1,2 | Pass |  | - |
|  | Liberia | Yes | One | Pass | No pass | - | - |  | 0.9917 |
|  | Libya | Yes | One | No pass | - | - | - |  | 0.9947 |
|  | Lithuania | Yes | One | Pass | No pass | - | - |  | 0.9931 |
|  | Luxembourg | No | One | No pass | - | - | - |  | 0.9995 |
|  | Madagascar | Yes | One | Pass | No pass | - | - |  | 0.9999 |
|  | Malawi | Yes | One | Pass | Pass | 2,1,1 | Pass |  | - |
|  | Malaysia | Yes | One | Pass | Pass | 1,1,2 | Pass |  | - |
|  | Maldives | Yes | One | Pass | Pass | 1,1,1 | Pass |  | - |
|  | Mali | Yes | One | Pass | Pass | 2,1,1 | Pass |  | - |
|  | Malta | Yes | One | Pass | No pass | - | - |  | 0.998 |
|  | Marshall Islands | No | One | Pass | No pass | - | - |  | 0.9984 |
|  | Mauritania | Yes | One | Pass | No pass | - | - |  | 0.9985 |
|  | Mauritius | Yes | One | Pass | Pass | 2,1,2 | Pass |  | - |
|  | Mexico | Yes | One | Pass | No pass | - | - |  | 0.9993 |
|  | Micronesia (Federated States of) | Yes | One | Pass | No pass | - | - |  | 0.9998 |
|  | Monaco | NO data | - | - | - | - | - |  | - |
|  | Mongolia | No | One | Pass | Pass | 2,1,2 | Pass |  | - |
|  | Montenegro | Yes | One | Pass | No pass | - | - |  | 0.9985 |
|  | Morocco | Yes | One | Pass | No pass | - | - |  | 0.999 |
|  | Mozambique | Yes | One | No pass | - | - | - |  | 0.9975 |
|  | Myanmar | Yes | Two | Pass | Pass | 1,2,1 | Pass |  | - |
|  | Namibia | Yes | One | Pass | Pass | 2,1,2 | Pass |  | - |
|  | Nauru | No | One | Pass | Pass | 1,1,2 | Pass |  | - |
|  | Nepal | Yes | Two | Pass | Pass | 2,1,1 | Pass |  | - |
|  | Netherlands | Yes | One | No pass | - | - | - |  | 0.9957 |
|  | New Zealand | Yes | One | Pass | No pass | - | - |  | 0.9969 |
|  | Nicaragua | Yes | One | Pass | No pass | - | - |  | 0.9995 |
|  | Niger | No | One | Pass | Pass | 0,1,0 | Pass |  | - |
|  | Nigeria | No | One | Pass | Pass | 1,1,2 | Pass |  | - |
|  | Niue | Yes | One | Pass | No pass | - | - |  | 0.9981 |
|  | Norway | Yes | One | No pass | - | - | - |  | 0.9988 |
|  | Oman | Yes | One | No pass | - | - | - |  | 0.9952 |
|  | Pakistan | Yes | One | Pass | No pass | - | - |  | 0.9964 |
|  | Palau | No | One | Pass | No pass | - | - |  | 0.9992 |
|  | Panama | Yes | One | Pass | No pass | - | - |  | 0.9974 |
|  | Papua New Guinea | Yes | One | Pass | No pass | - | - |  | 0.9999 |
|  | Paraguay | Yes | One | Pass | No pass | - | - |  | 0.9991 |
|  | Peru | No | One | Pass | Pass | 0,1,0 | Pass |  | - |
|  | Philippines | No | One | Pass | No pass | - | - |  | 0.9986 |
|  | Poland | Yes | One | Pass | No pass | - | - |  | 0.9962 |
|  | Portugal | Yes | One | Pass | No pass | - | - |  | 0.9953 |
|  | Qatar | Yes | One | No pass | - | - | - |  | 0.993 |
|  | Republic of Korea | Yes | One | Pass | - | - | - |  | 0.9984 |
|  | Republic of Moldova | No | One | Pass | Pass | 1,1,1 | Pass |  | - |
|  | Republic of North Macedonia | Yes | One | Pass | No pass | - | - |  | 0.9988 |
|  | Romania | Yes | One | Pass | No pass | - | - |  | 0.9979 |
|  | Russian Federation | Yes | One | No pass | - | - | - |  | 0.9697 |
|  | Rwanda | Yes | One | No pass | - | - | - |  | 0.9995 |
|  | Saint Kitts and Nevis | Yes | One | Pass | Pass | 1,1,2 | Pass |  | - |
|  | Saint Lucia | Yes | One | Pass | No pass | - | - |  | 0.9991 |
|  | Saint Vincent and the Grenadines | Yes | One | Pass | No pass | - | - |  | 0.9996 |
|  | Samoa | Yes | One | No pass | - | - | - |  | 0.9988 |
|  | San Marino | NO data | - | - | - | - | - |  | - |
|  | Sao Tome and Principe | Yes | One | Pass | No pass | - | - |  | 0.9978 |
|  | Saudi Arabia | No | One | Pass | No pass | - | - |  | 0.9994 |
|  | Senegal | Yes | One | No pass | - | - | - |  | 0.9999 |
|  | Serbia | Yes | One | Pass | No pass | - | - |  | 0.9993 |
|  | Seychelles | No | One | Pass | No pass | - | - |  | 0.9997 |
|  | Sierra Leone | No | One | Pass | No pass | - | - |  | 0.9999 |
|  | Singapore | Yes | One | Pass | Pass | 1,1,1 | Pass |  | - |
|  | Slovakia | Yes | One | No pass | - | - | - |  | 0.9955 |
|  | Slovenia | No | One | No pass | - | - | - |  | 0.997 |
|  | Solomon Islands | Yes | One | Pass | No pass | - | - |  | 0.9994 |
|  | Somalia | Yes | One | Pass | No pass | - | - |  | 0.9999 |
|  | South Africa | Yes | One | No pass | - | - | - |  | 0.9927 |
|  | South Sudan | NO data | - | - | - | - | - |  | - |
|  | Spain | No | One | No pass | - | - | - |  | 0.998 |
|  | Sri Lanka | Yes | One | Pass | Pass | 1,1,2 | Pass |  | - |
|  | Sudan | NO data | - | - | - | - | - |  | - |
|  | Sudan (former) | Yes | One | Pass | No pass | - | - |  | 0.9987 |
|  | Suriname | No | One | No pass | - | - | - |  | 0.9972 |
|  | Sweden | Yes | One | Pass | No pass | - | - |  | 0.9951 |
|  | Switzerland | Yes | One | No pass | - | - | - |  | 0.9821 |
|  | Syrian Arab Republic | Yes | One | Pass | No pass | - | - |  | 0.9996 |
|  | Tajikistan | Yes | One | Pass | Pass | 3,1,2 | Pass |  | - |
|  | Thailand | Yes | One | Pass | Pass | 0,1,0 | Pass |  | - |
|  | Timor-Leste | Yes | One | Pass | Pass | 1,1,2 | Pass |  | - |
|  | Togo | Yes | One | Pass | Pass | 0,1,0 | Pass |  | - |
|  | Tonga | Yes | One | No pass | - | - | - |  | 0.9981 |
|  | Trinidad and Tobago | Yes | One | Pass | Pass | 2,1,1 | Pass |  | - |
|  | Tunisia | Yes | One | Pass | No pass | - | - |  | 0.9985 |
|  | Turkey | Yes | One | No pass | - | - | - |  | 0.9965 |
|  | Turkmenistan | Yes | One | Pass | Pass | 1,1,0 | Pass |  | - |
|  | Tuvalu | Yes | One | No pass | - | - | - |  | 0.998 |
|  | Uganda | Yes | One | Pass | No pass | - | - |  | 0.9998 |
|  | Ukraine | No | One | Pass | Pass | 2,1,2 | Pass |  | - |
|  | United Arab Emirates | Yes | One | Pass | No pass | - | - |  | 0.9987 |
|  | United Kingdom of Great Britain and Northern Ireland | Yes | One | No pass | - | - | - |  | 0.9913 |
|  | United Republic of Tanzania | Yes | One | Pass | No pass | - | - |  | 0.9999 |
|  | United States of America | Yes | One | No pass | - | - | - |  | 0.9923 |
|  | Uruguay | No | One | Pass | Pass | 1,1,2 | Pass |  | - |
|  | Uzbekistan | No | One | Pass | No pass | - | - |  | 0.9981 |
|  | Vanuatu | Yes | One | Pass | No pass | - | - |  | 0.9993 |
|  | Venezuela (Bolivarian Republic of) | Yes | One | Pass | No pass | - | - |  | 0.9997 |
|  | Viet Nam | Yes | One | Pass | Pass | 0,1,1 | Pass |  | - |
|  | Yemen | Yes | One | Pass | No pass | - | - |  | 0.9997 |
|  | Zambia | Yes | One | No pass | - | - | - |  | 0.9975 |
|  | Zimbabwe | Yes | One | Pass | Pass | 1,1,2 | Pass |  | - |

^a^ Smoothing non-stationary time series through log transformation and difference. ^b^ Checking whether the transformed sequence meets the stationarity requirements. ^c^ Identifying model parameters based on autocorrelation function (ACF) graph and the partial autocorrelation function (PACF) graph, SCAN model and Bayesian Information Criterion (BIC). ^d^ Residual error test after model establishment. ^e^ Data that cannot be modeled by ARIMA, and were used trend extrapolation to build the model.

**Table S4. Accuracy evaluation of projection models of children's obesity prevalence by regions and country income**

|  |  | Boys | | | | | | |  | Girls | | | | | | |
| --- | --- | --- | --- | --- | --- | --- | --- | --- | --- | --- | --- | --- | --- | --- | --- | --- |
|  |  | 2011 | 2012 | 2013 | 2014 | 2015 | 2016 | Mean of relative error |  | 2011 | 2012 | 2013 | 2014 | 2015 | 2016 | Mean of relative error |
| Global | Measurement | 15.9 | 16.5 | 17.2 | 17.8 | 18.5 | 19.3 |  |  | 14.8 | 15.3 | 15.8 | 16.4 | 16.9 | 17.5 |  |
|  | Projection | 15.9 | 16.6 | 17.3 | 18.0 | 18.7 | 19.5 |  |  | 14.8 | 15.3 | 15.8 | 16.3 | 16.8 | 17.4 |  |
|  | Relative error | 0.2 | 0.5 | 0.3 | 0.9 | 1.1 | 0.9 | 0.7 |  | 0.0 | 0.3 | 0.0 | 0.7 | 0.3 | 0.7 | 0.3 |
| Africa | Measurement | 5.7 | 6.1 | 6.5 | 6.9 | 7.3 | 7.7 |  |  | 12.4 | 12.9 | 13.4 | 13.9 | 14.5 | 15.1 |  |
|  | Projection | 5.8 | 6.2 | 6.6 | 7.0 | 7.4 | 7.9 |  |  | 12.4 | 13.0 | 13.6 | 14.1 | 14.8 | 15.4 |  |
|  | Relative error | 1.3 | 0.8 | 0.8 | 1.2 | 1.9 | 3.0 | 1.5 |  | 0.3 | 0.6 | 1.1 | 1.8 | 1.8 | 2.1 | 1.3 |
| America | Measurement | 31.8 | 32.4 | 32.9 | 33.5 | 34.0 | 34.6 |  |  | 30.5 | 30.9 | 31.3 | 31.7 | 32.1 | 32.6 |  |
|  | Projection | 31.9 | 32.6 | 33.2 | 33.9 | 34.6 | 35.2 |  |  | 30.6 | 31.1 | 31.5 | 32.0 | 32.4 | 32.9 |  |
|  | Relative error | 0.3 | 0.5 | 1.0 | 1.2 | 1.7 | 1.9 | 1.1 |  | 0.3 | 0.5 | 0.7 | 0.9 | 1.0 | 0.9 | 0.7 |
| Southeast Asia | Measurement | 6.8 | 7.3 | 7.9 | 8.4 | 9.0 | 9.6 |  |  | 6.0 | 6.4 | 6.8 | 7.2 | 7.7 | 8.1 |  |
|  | Projection | 7.0 | 7.3 | 8.1 | 8.6 | 9.5 | 10.0 |  |  | 6.1 | 6.5 | 7.0 | 7.5 | 7.9 | 8.5 |  |
|  | Relative error | 2.3 | 0.7 | 2.7 | 2.0 | 5.1 | 4.1 | 2.8 |  | 1.3 | 2.0 | 2.3 | 3.7 | 3.2 | 5.2 | 2.9 |
| Europe | Measurement | 25.3 | 25.8 | 26.4 | 27.0 | 27.6 | 28.1 |  |  | 22.2 | 22.6 | 23.0 | 23.4 | 23.8 | 24.2 |  |
|  | Projection | 25.3 | 25.8 | 26.3 | 26.9 | 27.4 | 28.0 |  |  | 22.3 | 22.7 | 23.2 | 23.7 | 24.2 | 24.8 |  |
|  | Relative error | 0.2 | 0.0 | 0.2 | 0.4 | 0.6 | 0.4 | 0.3 |  | 0.3 | 0.6 | 1.0 | 1.4 | 1.8 | 2.3 | 1.2 |
| Mediterranean | Measurement | 17.0 | 17.6 | 18.2 | 18.8 | 19.5 | 20.2 |  |  | 18.0 | 18.5 | 19.0 | 19.6 | 20.1 | 20.7 |  |
|  | Projection | 17.1 | 17.6 | 18.2 | 18.8 | 19.4 | 20.0 |  |  | 17.9 | 18.3 | 18.7 | 19.2 | 19.6 | 20.0 |  |
|  | Relative error | 0.3 | 0.1 | 0.0 | 0.1 | 0.6 | 1.0 | 0.4 |  | 0.5 | 0.9 | 1.3 | 2.2 | 2.6 | 3.4 | 1.8 |
| Western Pacific | Measurement | 22.4 | 23.9 | 25.5 | 27.1 | 28.7 | 30.4 |  |  | 14.2 | 15.1 | 16.0 | 16.9 | 17.9 | 18.8 |  |
|  | Projection | 22.5 | 23.8 | 25.1 | 26.5 | 27.9 | 29.4 |  |  | 14.2 | 15.0 | 15.9 | 16.8 | 17.7 | 18.7 |  |
|  | Relative error | 0.4 | 0.5 | 1.4 | 2.2 | 2.7 | 3.3 | 1.7 |  | 0.2 | 0.4 | 0.7 | 0.7 | 1.0 | 0.4 | 0.6 |
| Low-income | Measurement | 5.5 | 5.8 | 6.1 | 6.4 | 6.8 | 7.2 |  |  | 11.4 | 11.9 | 12.4 | 13.0 | 13.5 | 14.1 |  |
|  | Projection | 5.5 | 5.8 | 6.2 | 6.5 | 7.0 | 7.3 |  |  | 11.4 | 11.9 | 12.4 | 12.9 | 13.4 | 14.0 |  |
|  | Relative error | 0.2 | 0.6 | 1.5 | 2.3 | 2.3 | 2.1 | 1.5 |  | 0.4 | 0.4 | 0.3 | 0.9 | 0.6 | 1.0 | 0.6 |
| Lower-middle-income | Measurement | 8.3 | 8.8 | 9.3 | 9.9 | 10.5 | 11.1 |  |  | 8.9 | 9.4 | 9.8 | 10.3 | 10.8 | 11.3 |  |
|  | Projection | 8.3 | 8.8 | 9.4 | 10.0 | 10.6 | 11.4 |  |  | 8.9 | 9.4 | 9.9 | 10.4 | 11.0 | 11.6 |  |
|  | Relative error | 0.1 | 0.0 | 1.2 | 1.2 | 1.1 | 2.3 | 1.0 |  | 0.6 | 0.2 | 1.2 | 1.4 | 1.8 | 2.5 | 1.3 |
| Upper-middle-income | Measurement | 25.1 | 26.4 | 27.8 | 29.2 | 30.5 | 31.9 |  |  | 20.1 | 21.0 | 21.8 | 22.7 | 23.6 | 24.4 |  |
|  | Projection | 25.0 | 26.2 | 27.5 | 28.8 | 30.1 | 31.4 |  |  | 20.0 | 20.8 | 21.6 | 22.4 | 23.2 | 24.0 |  |
|  | Relative error | 0.2 | 0.6 | 1.1 | 1.5 | 1.5 | 1.6 | 1.1 |  | 0.5 | 1.1 | 1.1 | 1.5 | 1.8 | 1.5 | 1.3 |
| High-income | Measurement | 33.2 | 33.6 | 34.0 | 34.4 | 34.8 | 35.1 |  |  | 29.1 | 29.4 | 29.7 | 29.9 | 30.2 | 30.5 |  |
|  | Projection | 33.3 | 33.8 | 34.3 | 34.8 | 35.3 | 35.8 |  |  | 29.1 | 29.5 | 29.9 | 30.3 | 30.7 | 31.1 |  |
|  | Relative error | 0.4 | 0.7 | 1.0 | 1.2 | 1.5 | 2.0 | 1.1 |  | 0.1 | 0.5 | 0.8 | 1.4 | 1.7 | 1.9 | 1.1 |

Relative error=100%*(|Measurement-Projection|/Projection)

**Table S5. Accuracy evaluation of projection models of children's obesity prevalence by countries**

|  |  | Boys | | | | | | |  | Girls | | | | | | |
| --- | --- | --- | --- | --- | --- | --- | --- | --- | --- | --- | --- | --- | --- | --- | --- | --- |
|  |  | 2011 | 2012 | 2013 | 2014 | 2015 | 2016 | Mean of relative error |  | 2011 | 2012 | 2013 | 2014 | 2015 | 2016 | Mean of relative error |
| Afghanistan | Measurement | 6.5 | 7.0 | 7.4 | 7.9 | 8.5 | 9.0 |  |  | 7.1 | 7.6 | 8.1 | 8.7 | 9.2 | 9.9 |  |
|  | Projection | 6.8 | 7.3 | 8.1 | 8.7 | 9.6 | 10.3 |  |  | 7.3 | 7.7 | 8.3 | 9.2 | 9.7 | 10.3 |  |
|  | Relative error | 4.6 | 4.3 | 9.5 | 10.1 | 12.9 | 14.4 | 9.3 |  | 2.8 | 1.3 | 2.5 | 5.7 | 5.4 | 4.0 | 3.6 |
| Albania | Measurement | 23.7 | 24.7 | 25.8 | 26.9 | 28.0 | 29.2 |  |  | 16.7 | 17.5 | 18.2 | 18.9 | 19.7 | 20.5 |  |
|  | Projection | 23.8 | 25.1 | 26.5 | 28.0 | 29.5 | 31.1 |  |  | 16.8 | 17.6 | 18.5 | 19.4 | 20.4 | 21.4 |  |
|  | Relative error | 0.4 | 1.6 | 2.7 | 4.1 | 5.4 | 6.5 | 3.5 |  | 0.6 | 0.6 | 1.6 | 2.6 | 3.6 | 4.4 | 2.2 |
| Algeria | Measurement | 26.4 | 27.5 | 28.5 | 29.5 | 30.6 | 31.6 |  |  | 26.9 | 27.6 | 28.3 | 28.9 | 29.6 | 30.3 |  |
|  | Projection | 26.5 | 27.5 | 28.5 | 29.6 | 30.7 | 31.8 |  |  | 27.0 | 27.8 | 28.5 | 29.3 | 30.0 | 30.8 |  |
|  | Relative error | 0.4 | 0.0 | 0.0 | 0.3 | 0.3 | 0.6 | 0.3 |  | 0.4 | 0.7 | 0.7 | 1.4 | 1.4 | 1.7 | 1.1 |
| Andorra | Measurement | 38.5 | 38.5 | 38.6 | 38.7 | 38.7 | 38.8 |  |  | 32.2 | 32.3 | 32.4 | 32.5 | 32.6 | 32.7 |  |
|  | Projection | 38.5 | 38.5 | 38.4 | 38.2 | 38.1 | 37.9 |  |  | 32.2 | 32.2 | 32.1 | 32.1 | 32.0 | 31.9 |  |
|  | Relative error | 0.0 | 0.0 | 0.5 | 1.3 | 1.6 | 2.3 | 1.0 |  | 0.0 | 0.3 | 0.9 | 1.2 | 1.8 | 2.4 | 1.1 |
| Angola | Measurement | 4.8 | 5.2 | 5.6 | 6.1 | 6.6 | 7.1 |  |  | 11.9 | 12.5 | 13.1 | 13.7 | 14.3 | 14.9 |  |
|  | Projection | 4.8 | 5.2 | 5.5 | 5.9 | 6.2 | 6.6 |  |  | 12.1 | 12.8 | 13.5 | 14.4 | 15.2 | 16.1 |  |
|  | Relative error | 0.0 | 0.0 | 1.8 | 3.3 | 6.1 | 7.0 | 3.0 |  | 1.7 | 2.4 | 3.1 | 5.1 | 6.3 | 8.1 | 4.5 |
| Antigua and Barbuda | Measurement | 21.1 | 22.0 | 22.9 | 23.9 | 24.9 | 26.0 |  |  | 23.3 | 24.1 | 24.9 | 25.7 | 26.6 | 27.5 |  |
|  | Projection | 21.3 | 22.1 | 23.0 | 23.9 | 24.7 | 25.7 |  |  | 23.4 | 24.1 | 24.8 | 25.6 | 26.3 | 27.1 |  |
|  | Relative error | 0.9 | 0.5 | 0.4 | 0.0 | 0.8 | 1.2 | 0.6 |  | 0.4 | 0.0 | 0.4 | 0.4 | 1.1 | 1.5 | 0.6 |
| Argentina | Measurement | 37.4 | 38.1 | 38.8 | 39.5 | 40.2 | 40.9 |  |  | 30.0 | 30.4 | 30.7 | 31.1 | 31.5 | 31.8 |  |
|  | Projection | 37.4 | 38.1 | 38.8 | 39.5 | 40.2 | 41.0 |  |  | 30.1 | 30.4 | 30.7 | 31.0 | 31.2 | 31.5 |  |
|  | Relative error | 0.0 | 0.0 | 0.0 | 0.0 | 0.0 | 0.2 | 0.0 |  | 0.3 | 0.0 | 0.0 | 0.3 | 1.0 | 0.9 | 0.4 |
| Armenia | Measurement | 16.1 | 16.6 | 17.1 | 17.6 | 18.1 | 18.6 |  |  | 18.5 | 18.8 | 19.1 | 19.4 | 19.7 | 20.1 |  |
|  | Projection | 16.3 | 16.6 | 16.9 | 17.2 | 17.5 | 17.9 |  |  | 18.6 | 18.7 | 18.9 | 19.1 | 19.2 | 19.4 |  |
|  | Relative error | 1.2 | 0.0 | 1.2 | 2.3 | 3.3 | 3.8 | 2.0 |  | 0.5 | 0.5 | 1.0 | 1.5 | 2.5 | 3.5 | 1.6 |
| Australia | Measurement | 34.2 | 34.4 | 34.7 | 35.0 | 35.3 | 35.5 |  |  | 31.9 | 32.1 | 32.2 | 32.3 | 32.4 | 32.6 |  |
|  | Projection | 34.2 | 34.6 | 35.0 | 35.4 | 35.8 | 36.2 |  |  | 32.1 | 32.3 | 32.6 | 32.8 | 33.0 | 33.2 |  |
|  | Relative error | 0.0 | 0.6 | 0.9 | 1.1 | 1.4 | 2.0 | 1.0 |  | 0.6 | 0.6 | 1.2 | 1.5 | 1.9 | 1.8 | 1.3 |
| Austria | Measurement | 27.8 | 28.1 | 28.4 | 28.8 | 29.1 | 29.4 |  |  | 22.0 | 22.3 | 22.6 | 22.9 | 23.2 | 23.5 |  |
|  | Projection | 27.9 | 28.3 | 28.6 | 28.9 | 29.2 | 29.5 |  |  | 22.1 | 22.4 | 22.6 | 22.9 | 23.1 | 23.4 |  |
|  | Relative error | 0.4 | 0.7 | 0.7 | 0.3 | 0.3 | 0.3 | 0.5 |  | 0.5 | 0.4 | 0.0 | 0.0 | 0.4 | 0.4 | 0.3 |
| Azerbaijan | Measurement | 15.4 | 16.0 | 16.6 | 17.2 | 17.9 | 18.6 |  |  | 16.9 | 17.2 | 17.7 | 18.1 | 18.5 | 19.0 |  |
|  | Projection | 15.6 | 16.1 | 16.6 | 17.0 | 17.5 | 18.0 |  |  | 16.8 | 17.0 | 17.3 | 17.5 | 17.8 | 18.1 |  |
|  | Relative error | 1.3 | 0.6 | 0.0 | 1.2 | 2.2 | 3.2 | 1.4 |  | 0.6 | 1.2 | 2.3 | 3.3 | 3.8 | 4.7 | 2.7 |
| Bahamas | Measurement | 32.8 | 33.4 | 34.1 | 34.7 | 35.5 | 36.2 |  |  | 33.1 | 33.5 | 33.9 | 34.4 | 34.9 | 35.4 |  |
|  | Projection | 33.0 | 33.6 | 34.3 | 34.9 | 35.5 | 36.1 |  |  | 33.2 | 33.6 | 33.9 | 34.3 | 34.6 | 35.0 |  |
|  | Relative error | 0.6 | 0.6 | 0.6 | 0.6 | 0.0 | 0.3 | 0.5 |  | 0.3 | 0.3 | 0.0 | 0.3 | 0.9 | 1.1 | 0.5 |
| Bahrain | Measurement | 33.6 | 34.2 | 34.7 | 35.3 | 36.0 | 36.7 |  |  | 32.2 | 32.5 | 32.8 | 33.1 | 33.5 | 33.8 |  |
|  | Projection | 33.4 | 34.1 | 34.7 | 35.4 | 36.0 | 36.7 |  |  | 32.2 | 32.5 | 32.8 | 33.1 | 33.5 | 33.8 |  |
|  | Relative error | 0.6 | 0.3 | 0.0 | 0.3 | 0.0 | 0.0 | 0.2 |  | 0.0 | 0.0 | 0.0 | 0.0 | 0.0 | 0.0 | 0.0 |
| Bangladesh | Measurement | 6.6 | 7.1 | 7.6 | 8.1 | 8.7 | 9.3 |  |  | 6.4 | 6.8 | 7.3 | 7.7 | 8.2 | 8.7 |  |
|  | Projection | 6.7 | 7.3 | 8.0 | 8.7 | 9.6 | 10.4 |  |  | 6.6 | 7.1 | 7.7 | 8.3 | 9.0 | 9.7 |  |
|  | Relative error | 1.5 | 2.8 | 5.3 | 7.4 | 10.3 | 11.8 | 6.5 |  | 3.1 | 4.4 | 5.5 | 7.8 | 9.8 | 11.5 | 7.0 |
| Barbados | Measurement | 21.9 | 22.8 | 23.9 | 24.9 | 26.0 | 27.2 |  |  | 23.7 | 24.5 | 25.4 | 26.2 | 26.7 | 27.6 |  |
|  | Projection | 22.0 | 22.9 | 23.9 | 24.9 | 25.9 | 27.0 |  |  | 23.7 | 24.5 | 25.4 | 26.2 | 27.1 | 28.0 |  |
|  | Relative error | 0.5 | 0.4 | 0.0 | 0.0 | 0.4 | 0.7 | 0.3 |  | 0.0 | 0.0 | 0.0 | 0.4 | 0.4 | 0.4 | 0.2 |
| Belarus | Measurement | 22.5 | 23.3 | 24.1 | 24.9 | 25.7 | 26.5 |  |  | 16.6 | 17.0 | 17.4 | 17.9 | 18.3 | 18.7 |  |
|  | Projection | 22.7 | 23.3 | 23.9 | 24.5 | 25.1 | 25.7 |  |  | 16.7 | 17.0 | 17.4 | 17.7 | 18.0 | 18.4 |  |
|  | Relative error | 0.9 | 0.0 | 0.8 | 1.6 | 2.3 | 3.0 | 1.4 |  | 0.6 | 0.0 | 0.0 | 1.1 | 1.6 | 1.6 | 0.8 |
| Belgium | Measurement | 24.3 | 24.2 | 24.0 | 23.9 | 23.8 | 23.6 |  |  | 24.7 | 24.6 | 24.5 | 24.5 | 24.4 | 24.3 |  |
|  | Projection | 24.5 | 24.3 | 24.1 | 23.8 | 23.5 | 23.2 |  |  | 24.9 | 24.8 | 24.7 | 24.5 | 24.3 | 24.1 |  |
|  | Relative error | 0.8 | 0.4 | 0.4 | 0.4 | 1.3 | 1.7 | 0.8 |  | 0.8 | 0.8 | 0.8 | 0.0 | 0.4 | 0.8 | 0.6 |
| Belize | Measurement | 24.0 | 24.9 | 25.7 | 26.6 | 27.5 | 28.4 |  |  | 25.3 | 25.9 | 26.6 | 27.3 | 28.0 | 28.7 |  |
|  | Projection | 24.1 | 24.9 | 25.8 | 26.7 | 27.6 | 28.5 |  |  | 25.4 | 26.0 | 26.7 | 27.4 | 28.1 | 28.9 |  |
|  | Relative error | 0.4 | 0.0 | 0.4 | 0.4 | 0.4 | 0.4 | 0.3 |  | 0.4 | 0.4 | 0.4 | 0.4 | 0.4 | 0.7 | 0.5 |
| Benin | Measurement | 5.3 | 5.7 | 6.0 | 6.4 | 6.7 | 7.1 |  |  | 13.0 | 13.5 | 14.0 | 14.5 | 15.0 | 15.6 |  |
|  | Projection | 5.3 | 5.6 | 6.0 | 6.3 | 6.7 | 7.1 |  |  | 13.0 | 13.5 | 14.0 | 14.5 | 15.0 | 15.5 |  |
|  | Relative error | 0.0 | 1.8 | 0.0 | 1.6 | 0.0 | 0.0 | 0.6 |  | 0.0 | 0.0 | 0.0 | 0.0 | 0.0 | 0.6 | 0.1 |
| Bhutan | Measurement | 7.7 | 8.2 | 8.8 | 9.4 | 10.1 | 10.7 |  |  | 7.0 | 7.4 | 7.9 | 8.4 | 8.9 | 9.4 |  |
|  | Projection | 8.2 | 9.0 | 9.7 | 11.1 | 12.1 | 13.0 |  |  | 7.2 | 7.8 | 8.4 | 9.1 | 9.9 | 10.7 |  |
|  | Relative error | 6.5 | 9.8 | 10.2 | 18.1 | 19.8 | 21.5 | 14.3 |  | 2.9 | 5.4 | 6.3 | 8.3 | 11.2 | 13.8 | 8.0 |
| Bolivia (Plurinational State of) | Measurement | 23.0 | 23.6 | 24.3 | 25.0 | 25.6 | 26.3 |  |  | 27.3 | 27.9 | 28.4 | 28.9 | 29.4 | 30.0 |  |
|  | Projection | 23.1 | 23.8 | 24.5 | 25.2 | 25.9 | 26.6 |  |  | 27.4 | 27.9 | 28.5 | 29.0 | 29.6 | 30.1 |  |
|  | Relative error | 0.4 | 0.8 | 0.8 | 0.8 | 1.2 | 1.1 | 0.9 |  | 0.4 | 0.0 | 0.4 | 0.3 | 0.7 | 0.3 | 0.4 |
| Bosnia and Herzegovina | Measurement | 20.7 | 21.6 | 22.4 | 23.3 | 24.3 | 25.2 |  |  | 14.4 | 15.0 | 15.6 | 16.2 | 16.8 | 17.5 |  |
|  | Projection | 20.8 | 21.6 | 22.4 | 23.3 | 24.1 | 25.0 |  |  | 14.4 | 14.9 | 15.4 | 16.0 | 16.6 | 17.1 |  |
|  | Relative error | 0.5 | 0.0 | 0.0 | 0.0 | 0.8 | 0.8 | 0.4 |  | 0.0 | 0.7 | 1.3 | 1.2 | 1.2 | 2.3 | 1.1 |
| Botswana | Measurement | 7.6 | 8.1 | 8.6 | 9.2 | 9.8 | 10.4 |  |  | 20.0 | 20.9 | 21.8 | 22.7 | 23.7 | 24.6 |  |
|  | Projection | 7.9 | 8.7 | 9.6 | 10.7 | 11.8 | 13.1 |  |  | 20.1 | 21.0 | 22.0 | 23.0 | 24.0 | 25.1 |  |
|  | Relative error | 3.9 | 7.4 | 11.6 | 16.3 | 20.4 | 26.0 | 14.3 |  | 0.5 | 0.5 | 0.9 | 1.3 | 1.3 | 2.0 | 1.1 |
| Brazil | Measurement | 26.2 | 26.9 | 27.6 | 28.3 | 29.0 | 29.7 |  |  | 24.3 | 24.7 | 25.1 | 25.5 | 25.9 | 26.3 |  |
|  | Projection | 26.1 | 26.8 | 27.6 | 28.3 | 29.1 | 29.9 |  |  | 24.2 | 24.7 | 25.1 | 25.5 | 26.0 | 26.4 |  |
|  | Relative error | 0.4 | 0.4 | 0.0 | 0.0 | 0.3 | 0.7 | 0.3 |  | 0.4 | 0.0 | 0.0 | 0.0 | 0.4 | 0.4 | 0.2 |
| Brunei Darussalam | Measurement | 26.5 | 27.6 | 28.6 | 29.7 | 30.7 | 31.7 |  |  | 18.4 | 19.1 | 19.7 | 20.4 | 21.0 | 21.7 |  |
|  | Projection | 26.4 | 27.5 | 28.6 | 29.8 | 30.9 | 32.1 |  |  | 18.4 | 19.1 | 19.8 | 20.5 | 21.2 | 21.9 |  |
|  | Relative error | 0.4 | 0.4 | 0.0 | 0.3 | 0.7 | 1.3 | 0.5 |  | 0.0 | 0.0 | 0.5 | 0.5 | 1.0 | 0.9 | 0.5 |
| Bulgaria | Measurement | 28.8 | 29.8 | 30.8 | 31.8 | 32.7 | 33.7 |  |  | 20.0 | 20.6 | 21.2 | 21.9 | 22.5 | 23.1 |  |
|  | Projection | 28.8 | 29.6 | 30.5 | 31.4 | 32.2 | 33.1 |  |  | 20.1 | 20.6 | 21.1 | 21.7 | 22.2 | 22.8 |  |
|  | Relative error | 0.0 | 0.7 | 1.0 | 1.3 | 1.5 | 1.8 | 1.1 |  | 0.5 | 0.0 | 0.5 | 0.9 | 1.3 | 1.3 | 0.8 |
| Burkina Faso | Measurement | 3.4 | 3.7 | 4.1 | 4.4 | 4.8 | 5.2 |  |  | 7.7 | 8.1 | 8.6 | 9.1 | 9.6 | 10.2 |  |
|  | Projection | 3.6 | 3.9 | 4.3 | 4.8 | 5.3 | 5.8 |  |  | 7.7 | 8.2 | 8.8 | 9.4 | 10.1 | 10.7 |  |
|  | Relative error | 5.9 | 5.4 | 4.9 | 9.1 | 10.4 | 11.5 | 7.9 |  | 0.0 | 1.2 | 2.3 | 3.3 | 5.2 | 4.9 | 2.8 |
| Burundi | Measurement | 4.4 | 4.7 | 5.1 | 5.5 | 5.9 | 6.3 |  |  | 11.1 | 11.7 | 12.3 | 13.0 | 13.6 | 14.3 |  |
|  | Projection | 4.5 | 5.0 | 5.5 | 6.0 | 6.6 | 7.2 |  |  | 11.3 | 12.0 | 12.8 | 13.7 | 14.7 | 15.8 |  |
|  | Relative error | 2.3 | 6.4 | 7.8 | 9.1 | 11.9 | 14.3 | 8.6 |  | 1.8 | 2.6 | 4.1 | 5.4 | 8.1 | 10.5 | 5.4 |
| Cabo Verde | Measurement | 6.7 | 7.0 | 7.4 | 7.7 | 8.1 | 8.5 |  |  | 14.2 | 14.6 | 15.0 | 15.4 | 15.8 | 16.2 |  |
|  | Projection | 6.8 | 7.1 | 7.6 | 8.0 | 8.6 | 9.1 |  |  | 14.2 | 14.6 | 15.1 | 15.6 | 16.1 | 16.6 |  |
|  | Relative error | 1.5 | 1.4 | 2.7 | 3.9 | 6.2 | 7.1 | 3.8 |  | 0.0 | 0.0 | 0.7 | 1.3 | 1.9 | 2.5 | 1.1 |
| Cambodia | Measurement | 9.2 | 10.0 | 10.8 | 11.7 | 12.7 | 13.7 |  |  | 6.4 | 6.8 | 7.3 | 7.7 | 8.2 | 8.7 |  |
|  | Projection | 9.3 | 10.1 | 11.0 | 12.1 | 13.2 | 14.4 |  |  | 6.6 | 7.1 | 7.6 | 8.1 | 8.8 | 9.4 |  |
|  | Relative error | 1.1 | 1.0 | 1.9 | 3.4 | 3.9 | 5.1 | 2.7 |  | 3.1 | 4.4 | 4.1 | 5.2 | 7.3 | 8.0 | 5.4 |
| Cameroon | Measurement | 6.2 | 6.5 | 6.9 | 7.2 | 7.6 | 8.0 |  |  | 15.3 | 15.8 | 16.2 | 16.6 | 17.0 | 17.4 |  |
|  | Projection | 6.2 | 6.5 | 6.8 | 7.2 | 7.5 | 7.8 |  |  | 15.4 | 15.8 | 16.3 | 16.8 | 17.3 | 17.8 |  |
|  | Relative error | 0.0 | 0.0 | 1.4 | 0.0 | 1.3 | 2.5 | 0.9 |  | 0.7 | 0.0 | 0.6 | 1.2 | 1.8 | 2.3 | 1.1 |
| Canada | Measurement | 32.5 | 32.8 | 33.1 | 33.4 | 33.7 | 34.0 |  |  | 29.0 | 29.2 | 29.4 | 29.6 | 29.8 | 30.0 |  |
|  | Projection | 32.6 | 33.0 | 33.4 | 33.8 | 34.2 | 34.6 |  |  | 29.9 | 30.2 | 30.5 | 30.9 | 31.2 | 31.5 |  |
|  | Relative error | 0.3 | 0.6 | 0.9 | 1.2 | 1.5 | 1.8 | 1.1 |  | 3.1 | 3.4 | 3.7 | 4.4 | 4.7 | 5.0 | 4.1 |
| Central African Republic | Measurement | 4.7 | 5.1 | 5.4 | 5.8 | 6.2 | 6.7 |  |  | 11.9 | 12.4 | 12.9 | 13.5 | 14.0 | 14.6 |  |
|  | Projection | 4.8 | 5.1 | 5.4 | 5.7 | 6.0 | 6.3 |  |  | 11.9 | 12.4 | 13.0 | 13.5 | 14.1 | 14.7 |  |
|  | Relative error | 2.1 | 0.0 | 0.0 | 1.7 | 3.2 | 6.0 | 2.2 |  | 0.0 | 0.0 | 0.8 | 0.0 | 0.7 | 0.7 | 0.4 |
| Chad | Measurement | 3.9 | 4.2 | 4.5 | 4.8 | 5.1 | 5.5 |  |  | 9.7 | 10.1 | 10.5 | 10.9 | 11.4 | 11.9 |  |
|  | Projection | 4.0 | 4.2 | 4.4 | 4.7 | 4.9 | 5.2 |  |  | 9.7 | 10.2 | 10.6 | 11.1 | 11.6 | 12.2 |  |
|  | Relative error | 2.6 | 0.0 | 2.2 | 2.1 | 3.9 | 5.5 | 2.7 |  | 0.0 | 1.0 | 1.0 | 1.8 | 1.8 | 2.5 | 1.4 |
| Chile | Measurement | 33.5 | 34.2 | 35.0 | 35.7 | 36.5 | 37.3 |  |  | 31.2 | 31.7 | 32.1 | 32.6 | 33.0 | 33.5 |  |
|  | Projection | 33.5 | 34.2 | 34.9 | 35.6 | 36.4 | 37.1 |  |  | 31.2 | 31.5 | 31.9 | 32.3 | 32.7 | 33.1 |  |
|  | Relative error | 0.0 | 0.0 | 0.3 | 0.3 | 0.3 | 0.5 | 0.2 |  | 0.0 | 0.6 | 0.6 | 0.9 | 0.9 | 1.2 | 0.7 |
| China | Measurement | 25.0 | 27.0 | 29.0 | 31.0 | 33.1 | 35.1 |  |  | 15.1 | 16.2 | 17.3 | 18.5 | 19.7 | 20.9 |  |
|  | Projection | 25.0 | 27.1 | 29.3 | 31.7 | 34.3 | 37.1 |  |  | 15.1 | 16.3 | 17.7 | 19.1 | 20.7 | 22.3 |  |
|  | Relative error | 0.0 | 0.4 | 1.0 | 2.3 | 3.6 | 5.7 | 2.2 |  | 0.0 | 0.6 | 2.3 | 3.2 | 5.1 | 6.7 | 3.0 |
| Colombia | Measurement | 20.1 | 20.5 | 21.0 | 21.5 | 22.0 | 22.5 |  |  | 23.8 | 24.3 | 24.8 | 25.3 | 25.7 | 26.2 |  |
|  | Projection | 20.1 | 20.6 | 21.0 | 21.4 | 21.8 | 22.3 |  |  | 23.8 | 24.2 | 24.7 | 25.1 | 25.5 | 25.9 |  |
|  | Relative error | 0.0 | 0.5 | 0.0 | 0.5 | 0.9 | 0.9 | 0.5 |  | 0.0 | 0.4 | 0.4 | 0.8 | 0.8 | 1.1 | 0.6 |
| Comoros | Measurement | 5.7 | 6.1 | 6.4 | 6.7 | 7.1 | 7.5 |  |  | 14.0 | 14.6 | 15.1 | 15.7 | 16.4 | 17.0 |  |
|  | Projection | 5.9 | 6.2 | 6.7 | 7.2 | 7.7 | 8.2 |  |  | 14.0 | 14.6 | 15.1 | 15.7 | 16.3 | 16.9 |  |
|  | Relative error | 3.5 | 1.6 | 4.7 | 7.5 | 8.5 | 9.3 | 5.9 |  | 0.0 | 0.0 | 0.0 | 0.0 | 0.6 | 0.6 | 0.2 |
| Congo | Measurement | 5.8 | 6.2 | 6.6 | 7.0 | 7.5 | 8.0 |  |  | 11.7 | 12.1 | 12.5 | 12.9 | 13.3 | 13.7 |  |
|  | Projection | 5.8 | 6.1 | 6.5 | 7.0 | 7.4 | 7.9 |  |  | 11.8 | 12.2 | 12.5 | 12.9 | 13.3 | 13.7 |  |
|  | Relative error | 0.0 | 1.6 | 1.5 | 0.0 | 1.3 | 1.3 | 1.0 |  | 0.9 | 0.8 | 0.0 | 0.0 | 0.0 | 0.0 | 0.3 |
| Cook Islands | Measurement | 55.0 | 56.1 | 57.2 | 58.2 | 59.2 | 60.2 |  |  | 62.1 | 62.9 | 63.7 | 64.5 | 65.2 | 66.0 |  |
|  | Projection | 54.7 | 56.1 | 57.6 | 59.1 | 60.6 | 62.1 |  |  | 62.0 | 63.1 | 64.2 | 65.4 | 66.5 | 67.6 |  |
|  | Relative error | 0.5 | 0.0 | 0.7 | 1.5 | 2.4 | 3.2 | 1.4 |  | 0.2 | 0.3 | 0.8 | 1.4 | 2.0 | 2.4 | 1.2 |
| Costa Rica | Measurement | 26.1 | 26.9 | 27.7 | 28.5 | 29.3 | 30.1 |  |  | 29.6 | 30.3 | 31.0 | 31.7 | 32.3 | 33.0 |  |
|  | Projection | 26.0 | 26.9 | 27.9 | 28.8 | 29.8 | 30.8 |  |  | 29.5 | 30.3 | 31.2 | 32.0 | 32.9 | 33.8 |  |
|  | Relative error | 0.4 | 0.0 | 0.7 | 1.1 | 1.7 | 2.3 | 1.0 |  | 0.3 | 0.0 | 0.6 | 0.9 | 1.9 | 2.4 | 1.0 |
| Cote d'Ivoire | Measurement | 6.8 | 7.2 | 7.6 | 8.0 | 8.4 | 8.9 |  |  | 14.6 | 15.0 | 15.5 | 16.0 | 16.4 | 16.9 |  |
|  | Projection | 7.0 | 7.5 | 8.0 | 8.6 | 9.2 | 9.8 |  |  | 14.5 | 15.0 | 15.5 | 16.0 | 16.5 | 17.1 |  |
|  | Relative error | 2.9 | 4.2 | 5.3 | 7.5 | 9.5 | 10.1 | 6.6 |  | 0.7 | 0.0 | 0.0 | 0.0 | 0.6 | 1.2 | 0.4 |
| Croatia | Measurement | 27.9 | 28.8 | 29.7 | 30.7 | 31.7 | 32.8 |  |  | 19.0 | 19.7 | 20.3 | 21.0 | 21.7 | 22.5 |  |
|  | Projection | 27.9 | 28.8 | 29.7 | 30.7 | 31.6 | 32.6 |  |  | 19.0 | 19.6 | 20.2 | 20.8 | 21.5 | 22.1 |  |
|  | Relative error | 0.0 | 0.0 | 0.0 | 0.0 | 0.3 | 0.6 | 0.2 |  | 0.0 | 0.5 | 0.5 | 1.0 | 0.9 | 1.8 | 0.8 |
| Cuba | Measurement | 27.8 | 28.4 | 29.1 | 29.7 | 30.3 | 31.0 |  |  | 27.1 | 27.5 | 27.9 | 28.2 | 28.6 | 29.0 |  |
|  | Projection | 27.9 | 28.5 | 29.1 | 29.7 | 30.3 | 31.0 |  |  | 27.2 | 27.5 | 27.8 | 28.2 | 28.5 | 28.8 |  |
|  | Relative error | 0.4 | 0.4 | 0.0 | 0.0 | 0.0 | 0.0 | 0.1 |  | 0.4 | 0.0 | 0.4 | 0.0 | 0.3 | 0.7 | 0.3 |
| Cyprus | Measurement | 34.4 | 34.7 | 35.1 | 35.5 | 35.9 | 36.2 |  |  | 28.0 | 28.3 | 28.7 | 29.0 | 29.4 | 29.8 |  |
|  | Projection | 34.8 | 34.9 | 35.1 | 35.2 | 35.3 | 35.4 |  |  | 28.2 | 28.5 | 28.7 | 28.9 | 29.0 | 29.2 |  |
|  | Relative error | 1.2 | 0.6 | 0.0 | 0.8 | 1.7 | 2.2 | 1.1 |  | 0.7 | 0.7 | 0.0 | 0.3 | 1.4 | 2.0 | 0.9 |
| Czechia | Measurement | 27.7 | 28.7 | 29.6 | 30.6 | 31.6 | 32.5 |  |  | 19.2 | 19.8 | 20.4 | 21.0 | 21.6 | 22.1 |  |
|  | Projection | 27.9 | 28.6 | 29.2 | 29.9 | 30.6 | 31.3 |  |  | 19.3 | 19.7 | 20.1 | 20.5 | 21.0 | 21.4 |  |
|  | Relative error | 0.7 | 0.3 | 1.4 | 2.3 | 3.2 | 3.7 | 1.9 |  | 0.5 | 0.5 | 1.5 | 2.4 | 2.8 | 3.2 | 1.8 |
| Democratic People's Republic of Korea | Measurement | 23.3 | 24.2 | 25.2 | 26.2 | 27.2 | 28.3 |  |  | 14.3 | 14.8 | 15.4 | 16.0 | 16.6 | 17.3 |  |
|  | Projection | 23.3 | 24.2 | 25.2 | 26.1 | 27.1 | 28.0 |  |  | 14.4 | 14.9 | 15.4 | 15.9 | 16.4 | 17.0 |  |
|  | Relative error | 0.0 | 0.0 | 0.0 | 0.4 | 0.4 | 1.1 | 0.3 |  | 0.7 | 0.7 | 0.0 | 0.6 | 1.2 | 1.7 | 0.8 |
| Democratic Republic of the Congo | Measurement | 4.3 | 4.7 | 5.0 | 5.4 | 5.8 | 6.2 |  |  | 11.3 | 11.8 | 12.4 | 12.9 | 13.5 | 14.1 |  |
|  | Projection | 4.3 | 4.6 | 5.0 | 5.4 | 5.8 | 6.3 |  |  | 11.4 | 12.1 | 12.7 | 13.5 | 14.2 | 15.0 |  |
|  | Relative error | 0.0 | 2.1 | 0.0 | 0.0 | 0.0 | 1.6 | 0.6 |  | 0.9 | 2.5 | 2.4 | 4.7 | 5.2 | 6.4 | 3.7 |
| Denmark | Measurement | 26.8 | 26.8 | 26.8 | 26.9 | 26.9 | 26.9 |  |  | 23.1 | 23.1 | 23.0 | 23.0 | 23.0 | 22.9 |  |
|  | Projection | 27.0 | 27.0 | 27.0 | 26.9 | 26.9 | 26.8 |  |  | 23.3 | 23.2 | 23.1 | 23.0 | 22.8 | 22.6 |  |
|  | Relative error | 0.7 | 0.7 | 0.7 | 0.0 | 0.0 | 0.4 | 0.4 |  | 0.9 | 0.4 | 0.4 | 0.0 | 0.9 | 1.3 | 0.7 |
| Djibouti | Measurement | 11.4 | 11.7 | 12.0 | 12.4 | 12.7 | 13.1 |  |  | 19.8 | 20.1 | 20.4 | 20.7 | 21.1 | 21.4 |  |
|  | Projection | 11.6 | 11.8 | 12.0 | 12.2 | 12.5 | 12.7 |  |  | 19.8 | 20.1 | 20.3 | 20.5 | 20.7 | 20.9 |  |
|  | Relative error | 1.8 | 0.9 | 0.0 | 1.6 | 1.6 | 3.1 | 1.5 |  | 0.0 | 0.0 | 0.5 | 1.0 | 1.9 | 2.3 | 1.0 |
| Dominica | Measurement | 27.9 | 28.7 | 29.5 | 30.4 | 31.2 | 32.0 |  |  | 30.2 | 30.8 | 31.4 | 32.0 | 32.6 | 33.2 |  |
|  | Projection | 28.1 | 28.9 | 29.7 | 30.5 | 31.3 | 32.1 |  |  | 30.4 | 31.0 | 31.5 | 32.1 | 32.6 | 33.2 |  |
|  | Relative error | 0.7 | 0.7 | 0.7 | 0.3 | 0.3 | 0.3 | 0.5 |  | 0.7 | 0.6 | 0.3 | 0.3 | 0.0 | 0.0 | 0.3 |
| Dominican Republic | Measurement | 28.4 | 29.4 | 30.3 | 31.3 | 32.2 | 33.1 |  |  | 29.3 | 30.0 | 30.7 | 31.3 | 32.0 | 32.6 |  |
|  | Projection | 28.1 | 29.1 | 30.2 | 31.2 | 32.2 | 33.3 |  |  | 29.1 | 29.8 | 30.6 | 31.3 | 32.1 | 32.9 |  |
|  | Relative error | 1.1 | 1.0 | 0.3 | 0.3 | 0.0 | 0.6 | 0.6 |  | 0.7 | 0.7 | 0.3 | 0.0 | 0.3 | 0.9 | 0.5 |
| Ecuador | Measurement | 23.9 | 24.6 | 25.3 | 26.0 | 26.7 | 27.5 |  |  | 26.3 | 26.9 | 27.4 | 28.0 | 28.6 | 29.1 |  |
|  | Projection | 23.9 | 24.6 | 25.3 | 26.1 | 26.8 | 27.5 |  |  | 26.4 | 26.9 | 27.5 | 28.0 | 28.5 | 29.1 |  |
|  | Relative error | 0.0 | 0.0 | 0.0 | 0.4 | 0.4 | 0.0 | 0.1 |  | 0.4 | 0.0 | 0.4 | 0.0 | 0.3 | 0.0 | 0.2 |
| Egypt | Measurement | 31.1 | 32.1 | 33.0 | 34.0 | 35.0 | 36.0 |  |  | 33.4 | 34.2 | 35.0 | 35.8 | 36.6 | 37.4 |  |
|  | Projection | 30.9 | 31.8 | 32.7 | 33.6 | 34.6 | 35.5 |  |  | 33.4 | 34.3 | 35.2 | 36.1 | 37.0 | 37.9 |  |
|  | Relative error | 0.6 | 0.9 | 0.9 | 1.2 | 1.1 | 1.4 | 1.0 |  | 0.0 | 0.3 | 0.6 | 0.8 | 1.1 | 1.3 | 0.7 |
| El Salvador | Measurement | 24.3 | 24.9 | 25.5 | 26.2 | 26.9 | 27.7 |  |  | 30.2 | 30.8 | 31.4 | 32.0 | 32.6 | 33.3 |  |
|  | Projection | 24.3 | 25.1 | 25.9 | 26.7 | 27.5 | 28.4 |  |  | 30.3 | 31.0 | 31.8 | 32.6 | 33.4 | 34.2 |  |
|  | Relative error | 0.0 | 0.8 | 1.6 | 1.9 | 2.2 | 2.5 | 1.5 |  | 0.3 | 0.6 | 1.3 | 1.9 | 2.5 | 2.7 | 1.6 |
| Equatorial Guinea | Measurement | 4.8 | 5.1 | 5.5 | 5.8 | 6.3 | 6.7 |  |  | 12.0 | 12.5 | 13.0 | 13.6 | 14.1 | 14.7 |  |
|  | Projection | 4.8 | 5.2 | 5.5 | 5.9 | 6.4 | 6.8 |  |  | 12.1 | 12.6 | 13.1 | 13.6 | 14.1 | 14.6 |  |
|  | Relative error | 0.0 | 2.0 | 0.0 | 1.7 | 1.6 | 1.5 | 1.1 |  | 0.8 | 0.8 | 0.8 | 0.0 | 0.0 | 0.7 | 0.5 |
| Eritrea | Measurement | 4.6 | 4.9 | 5.2 | 5.6 | 6.0 | 6.4 |  |  | 12.2 | 12.8 | 13.4 | 14.0 | 14.7 | 15.3 |  |
|  | Projection | 4.6 | 5.0 | 5.4 | 5.8 | 6.4 | 6.9 |  |  | 12.1 | 12.7 | 13.3 | 13.9 | 14.5 | 15.1 |  |
|  | Relative error | 0.0 | 2.0 | 3.8 | 3.6 | 6.7 | 7.8 | 4.0 |  | 0.8 | 0.8 | 0.7 | 0.7 | 1.4 | 1.3 | 1.0 |
| Estonia | Measurement | 19.8 | 20.4 | 21.0 | 21.6 | 22.3 | 22.9 |  |  | 16.2 | 16.6 | 17.0 | 17.4 | 17.8 | 18.2 |  |
|  | Projection | 19.8 | 20.2 | 20.7 | 21.1 | 21.5 | 21.9 |  |  | 16.1 | 16.4 | 16.7 | 17.0 | 17.3 | 17.6 |  |
|  | Relative error | 0.0 | 1.0 | 1.4 | 2.3 | 3.6 | 4.4 | 2.1 |  | 0.6 | 1.2 | 1.8 | 2.3 | 2.8 | 3.3 | 2.0 |
| Eswatini | Measurement | 5.3 | 5.8 | 6.4 | 7.0 | 7.6 | 8.3 |  |  | 18.7 | 19.8 | 21.1 | 22.3 | 23.7 | 25.0 |  |
|  | Projection | 5.3 | 5.9 | 6.4 | 7.7 | 8.5 |  |  |  | 18.8 | 20.2 | 21.6 | 23.2 | 24.9 | 26.7 |  |
|  | Relative error | 0.0 | 1.7 | 0.0 | 1.4 | 1.3 | 2.4 | 1.1 |  | 0.5 | 2.0 | 2.4 | 4.0 | 5.1 | 6.8 | 3.5 |
| Ethiopia | Measurement | 3.4 | 3.7 | 3.9 | 4.1 | 4.4 | 4.7 |  |  | 9.9 | 10.4 | 10.9 | 11.4 | 11.9 | 12.5 |  |
|  | Projection | 3.6 | 3.9 | 4.3 | 4.6 | 5.0 | 5.4 |  |  | 10.1 | 10.7 | 11.4 | 12.2 | 12.9 | 13.7 |  |
|  | Relative error | 5.9 | 5.4 | 10.3 | 12.2 | 13.6 | 14.9 | 10.4 |  | 2.0 | 2.9 | 4.6 | 7.0 | 8.4 | 9.6 | 5.8 |
| Fiji | Measurement | 25.4 | 26.2 | 27.0 | 27.8 | 28.6 | 29.4 |  |  | 36.7 | 37.3 | 37.9 | 38.5 | 39.0 | 39.6 |  |
|  | Projection | 25.5 | 26.3 | 27.1 | 27.9 | 28.8 | 29.6 |  |  | 36.9 | 37.6 | 38.2 | 38.9 | 39.5 | 40.2 |  |
|  | Relative error | 0.4 | 0.4 | 0.4 | 0.4 | 0.7 | 0.7 | 0.5 |  | 0.5 | 0.8 | 0.8 | 1.0 | 1.3 | 1.5 | 1.0 |
| Finland | Measurement | 28.3 | 28.5 | 28.8 | 29.0 | 29.2 | 29.5 |  |  | 23.2 | 23.3 | 23.5 | 23.6 | 23.8 | 23.9 |  |
|  | Projection | 28.6 | 28.7 | 28.8 | 28.9 | 29.0 | 29.1 |  |  | 23.4 | 23.5 | 23.5 | 23.5 | 23.5 | 23.5 |  |
|  | Relative error | 1.1 | 0.7 | 0.0 | 0.3 | 0.7 | 1.4 | 0.7 |  | 0.9 | 0.9 | 0.0 | 0.4 | 1.3 | 1.7 | 0.9 |
| France | Measurement | 29.9 | 30.2 | 30.4 | 30.7 | 30.9 | 31.2 |  |  | 27.4 | 27.7 | 28.0 | 28.2 | 28.5 | 28.8 |  |
|  | Projection | 29.8 | 30.1 | 30.4 | 30.7 | 31.0 | 31.3 |  |  | 27.4 | 27.7 | 28.0 | 28.4 | 28.7 | 29.0 |  |
|  | Relative error | 0.3 | 0.3 | 0.0 | 0.0 | 0.3 | 0.3 | 0.2 |  | 0.0 | 0.0 | 0.0 | 0.7 | 0.7 | 0.7 | 0.4 |
| Gabon | Measurement | 9.7 | 10.1 | 10.4 | 10.8 | 11.3 | 11.7 |  |  | 17.9 | 18.3 | 18.6 | 18.9 | 19.3 | 19.6 |  |
|  | Projection | 9.8 | 10.2 | 10.6 | 11.0 | 11.4 | 11.8 |  |  | 18.0 | 18.4 | 18.8 | 19.1 | 19.5 | 19.9 |  |
|  | Relative error | 1.0 | 1.0 | 1.9 | 1.9 | 0.9 | 0.9 | 1.3 |  | 0.6 | 0.5 | 1.1 | 1.1 | 1.0 | 1.5 | 1.0 |
| Gambia | Measurement | 6.4 | 6.7 | 7.1 | 7.5 | 7.9 | 8.3 |  |  | 13.2 | 13.6 | 14.0 | 14.4 | 14.8 | 15.2 |  |
|  | Projection | 6.5 | 7.0 | 7.5 | 8.1 | 8.7 | 9.3 |  |  | 13.2 | 13.6 | 14.1 | 14.6 | 15.1 | 15.6 |  |
|  | Relative error | 1.6 | 4.5 | 5.6 | 8.0 | 10.1 | 12.0 | 7.0 |  | 0.0 | 0.0 | 0.7 | 1.4 | 2.0 | 2.6 | 1.1 |
| Georgia | Measurement | 16.9 | 17.6 | 18.4 | 19.3 | 20.1 | 20.9 |  |  | 16.7 | 17.2 | 17.6 | 18.1 | 18.6 | 19.1 |  |
|  | Projection | 17.2 | 17.7 | 18.2 | 18.8 | 19.3 | 19.9 |  |  | 16.9 | 17.2 | 17.5 | 17.8 | 18.0 | 18.3 |  |
|  | Relative error | 1.8 | 0.6 | 1.1 | 2.6 | 4.0 | 4.8 | 2.5 |  | 1.2 | 0.0 | 0.6 | 1.7 | 3.2 | 4.2 | 1.8 |
| Germany | Measurement | 26.9 | 27.1 | 27.3 | 27.6 | 27.8 | 28.1 |  |  | 23.4 | 23.7 | 23.9 | 24.1 | 24.3 | 24.6 |  |
|  | Projection | 39.2 | 40.1 | 41.0 | 42.0 | 42.9 | 43.8 |  |  | 23.6 | 23.8 | 23.9 | 24.1 | 24.3 | 24.5 |  |
|  | Relative error | 45.7 | 48.0 | 50.2 | 52.2 | 54.3 | 55.9 | 51.1 |  | 0.9 | 0.4 | 0.0 | 0.0 | 0.0 | 0.4 | 0.3 |
| Ghana | Measurement | 5.0 | 5.3 | 5.5 | 5.8 | 6.1 | 6.4 |  |  | 13.0 | 13.5 | 13.9 | 14.3 | 14.8 | 15.2 |  |
|  | Projection | 5.1 | 5.4 | 5.8 | 6.2 | 6.6 | 7.0 |  |  | 13.0 | 13.5 | 14.0 | 14.5 | 15.0 | 15.5 |  |
|  | Relative error | 2.0 | 1.9 | 5.5 | 6.9 | 8.2 | 9.4 | 5.7 |  | 0.0 | 0.0 | 0.7 | 1.4 | 1.4 | 2.0 | 0.9 |
| Greece | Measurement | 39.0 | 39.4 | 39.9 | 40.4 | 40.8 | 41.3 |  |  | 31.0 | 31.4 | 31.8 | 32.3 | 32.7 | 33.1 |  |
|  | Projection | 38.9 | 39.4 | 39.9 | 40.4 | 40.9 | 41.4 |  |  | 30.9 | 31.4 | 31.8 | 32.3 | 32.7 | 33.2 |  |
|  | Relative error | 0.3 | 0.0 | 0.0 | 0.0 | 0.2 | 0.2 | 0.1 |  | 0.3 | 0.0 | 0.0 | 0.0 | 0.0 | 0.3 | 0.1 |
| Grenada | Measurement | 20.9 | 21.9 | 22.8 | 23.8 | 24.8 | 25.9 |  |  | 22.9 | 23.6 | 24.4 | 25.2 | 26.1 | 26.9 |  |
|  | Projection | 21.0 | 22.0 | 23.1 | 24.2 | 25.4 | 26.6 |  |  | 22.9 | 23.7 | 24.5 | 25.4 | 26.3 | 27.2 |  |
|  | Relative error | 0.5 | 0.5 | 1.3 | 1.7 | 2.4 | 2.7 | 1.5 |  | 0.0 | 0.4 | 0.4 | 0.8 | 0.8 | 1.1 | 0.6 |
| Guatemala | Measurement | 22.5 | 23.3 | 24.0 | 24.8 | 25.7 | 26.5 |  |  | 27.0 | 27.7 | 28.5 | 29.2 | 29.9 | 30.7 |  |
|  | Projection | 22.5 | 23.3 | 24.1 | 25.0 | 25.8 | 26.7 |  |  | 27.0 | 27.8 | 28.6 | 29.4 | 30.2 | 31.1 |  |
|  | Relative error | 0.0 | 0.0 | 0.4 | 0.8 | 0.4 | 0.8 | 0.4 |  | 0.0 | 0.4 | 0.4 | 0.7 | 1.0 | 1.3 | 0.6 |
| Guinea | Measurement | 4.1 | 4.4 | 4.6 | 4.9 | 5.3 | 5.6 |  |  | 11.1 | 11.6 | 12.0 | 12.5 | 13.0 | 13.5 |  |
|  | Projection | 4.3 | 4.5 | 4.9 | 5.3 | 5.7 | 6.1 |  |  | 11.1 | 11.5 | 11.9 | 12.4 | 12.9 | 13.3 |  |
|  | Relative error | 4.9 | 2.3 | 6.5 | 8.2 | 7.5 | 8.9 | 6.4 |  | 0.0 | 0.9 | 0.8 | 0.8 | 0.8 | 1.5 | 0.8 |
| Guinea-Bissau | Measurement | 5.4 | 5.7 | 6.1 | 6.4 | 6.8 | 7.3 |  |  | 12.2 | 12.6 | 13.1 | 13.6 | 14.1 | 14.6 |  |
|  | Projection | 5.4 | 5.6 | 6.0 | 6.4 | 6.8 | 7.1 |  |  | 12.2 | 12.8 | 13.4 | 14.0 | 14.6 | 15.3 |  |
|  | Relative error | 0.0 | 1.8 | 1.6 | 0.0 | 0.0 | 2.7 | 1.0 |  | 0.0 | 1.6 | 2.3 | 2.9 | 3.5 | 4.8 | 2.5 |
| Guyana | Measurement | 19.4 | 20.3 | 21.2 | 22.2 | 23.1 | 24.0 |  |  | 21.8 | 22.6 | 23.4 | 24.2 | 25.1 | 25.9 |  |
|  | Projection | 19.5 | 20.6 | 21.7 | 22.9 | 24.1 | 25.4 |  |  | 21.9 | 22.8 | 23.8 | 24.8 | 25.9 | 27.0 |  |
|  | Relative error | 0.5 | 1.5 | 2.4 | 3.2 | 4.3 | 5.8 | 3.0 |  | 0.5 | 0.9 | 1.7 | 2.5 | 3.2 | 4.2 | 2.2 |
| Haiti | Measurement | 24.5 | 25.7 | 26.9 | 28.1 | 29.3 | 30.5 |  |  | 20.9 | 21.7 | 22.4 | 23.1 | 23.9 | 24.6 |  |
|  | Projection | 24.1 | 25.4 | 26.7 | 28.0 | 29.4 | 30.8 |  |  | 20.7 | 21.5 | 22.4 | 23.2 | 24.1 | 25.0 |  |
|  | Relative error | 1.6 | 1.2 | 0.7 | 0.4 | 0.3 | 1.0 | 0.9 |  | 1.0 | 0.9 | 0.0 | 0.4 | 0.8 | 1.6 | 0.8 |
| Honduras | Measurement | 21.1 | 21.8 | 22.6 | 23.4 | 24.2 | 25.0 |  |  | 26.0 | 26.7 | 27.4 | 28.2 | 28.9 | 29.6 |  |
|  | Projection | 21.1 | 21.9 | 22.7 | 23.5 | 24.3 | 25.2 |  |  | 26.0 | 26.8 | 27.6 | 28.4 | 29.2 | 30.0 |  |
|  | Relative error | 0.0 | 0.5 | 0.4 | 0.4 | 0.4 | 0.8 | 0.4 |  | 0.0 | 0.4 | 0.7 | 0.7 | 1.0 | 1.4 | 0.7 |
| Hungary | Measurement | 27.6 | 28.6 | 29.6 | 30.7 | 31.7 | 32.7 |  |  | 20.5 | 21.1 | 21.8 | 22.5 | 23.2 | 23.8 |  |
|  | Projection | 27.6 | 28.4 | 29.3 | 30.1 | 31.0 | 31.9 |  |  | 20.4 | 21.1 | 21.7 | 22.4 | 23.1 | 23.9 |  |
|  | Relative error | 0.0 | 0.7 | 1.0 | 2.0 | 2.2 | 2.4 | 1.4 |  | 0.5 | 0.0 | 0.5 | 0.4 | 0.4 | 0.4 | 0.4 |
| Iceland | Measurement | 30.1 | 30.2 | 30.3 | 30.4 | 30.5 | 30.7 |  |  | 25.8 | 25.8 | 25.8 | 25.9 | 25.9 | 26.0 |  |
|  | Projection | 30.4 | 30.4 | 30.4 | 30.4 | 30.4 | 30.4 |  |  | 26.0 | 25.9 | 25.9 | 25.9 | 25.8 | 25.8 |  |
|  | Relative error | 1.0 | 0.7 | 0.3 | 0.0 | 0.3 | 1.0 | 0.6 |  | 0.8 | 0.4 | 0.4 | 0.0 | 0.4 | 0.8 | 0.5 |
| India | Measurement | 5.0 | 5.5 | 5.9 | 6.4 | 6.9 | 7.4 |  |  | 4.4 | 4.7 | 5.0 | 5.3 | 5.7 | 6.1 |  |
|  | Projection | 5.0 | 5.4 | 5.8 | 6.3 | 6.8 | 7.4 |  |  | 4.5 | 4.7 | 5.0 | 5.3 | 5.6 | 5.8 |  |
|  | Relative error | 0.0 | 1.8 | 1.7 | 1.6 | 1.4 | 0.0 | 1.1 |  | 2.3 | 0.0 | 0.0 | 0.0 | 1.8 | 4.9 | 1.5 |
| Indonesia | Measurement | 12.3 | 13.1 | 13.9 | 14.8 | 15.6 | 16.5 |  |  | 11.3 | 11.8 | 12.4 | 13.0 | 13.6 | 14.2 |  |
|  | Projection | 12.7 | 13.9 | 15.2 | 16.6 | 18.2 | 19.9 |  |  | 11.2 | 11.8 | 12.4 | 13.0 | 13.6 | 14.3 |  |
|  | Relative error | 3.3 | 6.1 | 9.4 | 12.2 | 16.7 | 20.6 | 11.4 |  | 0.9 | 0.0 | 0.0 | 0.0 | 0.0 | 0.7 | 0.3 |
| Iran (Islamic Republic of) | Measurement | 23.1 | 23.7 | 24.4 | 25.0 | 25.6 | 26.2 |  |  | 23.2 | 23.6 | 23.9 | 24.3 | 24.6 | 24.9 |  |
|  | Projection | 23.0 | 23.7 | 24.5 | 25.3 | 26.0 | 26.8 |  |  | 23.2 | 23.7 | 24.2 | 24.7 | 25.3 | 25.8 |  |
|  | Relative error | 0.4 | 0.0 | 0.4 | 1.2 | 1.6 | 2.3 | 1.0 |  | 0.0 | 0.4 | 1.3 | 1.6 | 2.8 | 3.6 | 1.6 |
| Iraq | Measurement | 28.5 | 29.3 | 30.0 | 30.7 | 31.5 | 32.2 |  |  | 29.2 | 29.7 | 30.2 | 30.7 | 31.3 | 31.8 |  |
|  | Projection | 28.4 | 29.1 | 29.8 | 30.6 | 31.3 | 32.0 |  |  | 29.2 | 29.7 | 30.2 | 30.6 | 31.1 | 31.6 |  |
|  | Relative error | 0.4 | 0.7 | 0.7 | 0.3 | 0.6 | 0.6 | 0.6 |  | 0.0 | 0.0 | 0.0 | 0.3 | 0.6 | 0.6 | 0.3 |
| Ireland | Measurement | 29.1 | 29.7 | 30.2 | 30.7 | 31.3 | 31.8 |  |  | 28.4 | 28.7 | 29.1 | 29.5 | 29.8 | 30.2 |  |
|  | Projection | 29.0 | 29.7 | 30.3 | 31.0 | 31.7 | 32.4 |  |  | 28.3 | 28.8 | 29.2 | 29.7 | 30.2 | 30.7 |  |
|  | Relative error | 0.3 | 0.0 | 0.3 | 1.0 | 1.3 | 1.9 | 0.8 |  | 0.4 | 0.3 | 0.3 | 0.7 | 1.3 | 1.7 | 0.8 |
| Israel | Measurement | 37.2 | 37.3 | 37.4 | 37.5 | 37.6 | 37.8 |  |  | 31.4 | 31.6 | 31.7 | 31.9 | 32.0 | 32.2 |  |
|  | Projection | 37.2 | 37.3 | 37.5 | 37.6 | 37.6 | 37.7 |  |  | 31.5 | 31.6 | 31.8 | 32.0 | 32.2 | 32.3 |  |
|  | Relative error | 0.0 | 0.0 | 0.3 | 0.3 | 0.0 | 0.3 | 0.2 |  | 0.3 | 0.0 | 0.3 | 0.3 | 0.6 | 0.3 | 0.3 |
| Italy | Measurement | 37.8 | 38.1 | 38.4 | 38.7 | 39.1 | 39.4 |  |  | 32.2 | 32.6 | 33.0 | 33.3 | 33.7 | 34.0 |  |
|  | Projection | 37.8 | 38.2 | 38.6 | 38.9 | 39.3 | 39.6 |  |  | 32.3 | 32.7 | 33.2 | 33.7 | 34.1 | 34.6 |  |
|  | Relative error | 0.0 | 0.3 | 0.5 | 0.5 | 0.5 | 0.5 | 0.4 |  | 0.3 | 0.3 | 0.6 | 1.2 | 1.2 | 1.8 | 0.9 |
| Jamaica | Measurement | 24.3 | 25.2 | 26.0 | 26.9 | 27.8 | 28.8 |  |  | 27.2 | 27.9 | 28.6 | 29.2 | 30.0 | 30.7 |  |
|  | Projection | 24.3 | 25.2 | 26.0 | 27.0 | 27.9 | 28.8 |  |  | 27.3 | 28.0 | 28.7 | 29.4 | 30.2 | 30.9 |  |
|  | Relative error | 0.0 | 0.0 | 0.0 | 0.4 | 0.4 | 0.0 | 0.1 |  | 0.4 | 0.4 | 0.3 | 0.7 | 0.7 | 0.7 | 0.5 |
| Japan | Measurement | 17.1 | 17.1 | 16.9 | 16.8 | 16.7 | 16.6 |  |  | 11.9 | 11.9 | 11.9 | 11.8 | 11.8 | 11.8 |  |
|  | Projection | 16.9 | 16.9 | 16.8 | 16.8 | 16.7 | 16.6 |  |  | 11.9 | 11.9 | 12.0 | 12.0 | 12.1 | 12.1 |  |
|  | Relative error | 1.2 | 1.2 | 0.6 | 0.0 | 0.0 | 0.0 | 0.5 |  | 0.0 | 0.0 | 0.8 | 1.7 | 2.5 | 2.5 | 1.3 |
| Jordan | Measurement | 28.1 | 28.8 | 29.4 | 30.0 | 30.7 | 31.4 |  |  | 28.9 | 29.2 | 29.6 | 29.9 | 30.3 | 30.7 |  |
|  | Projection | 28.4 | 29.1 | 29.8 | 30.5 | 31.2 | 32.0 |  |  | 29.1 | 29.5 | 29.9 | 30.3 | 30.7 | 31.1 |  |
|  | Relative error | 1.1 | 1.0 | 1.4 | 1.7 | 1.6 | 1.9 | 1.5 |  | 0.7 | 1.0 | 1.0 | 1.3 | 1.3 | 1.3 | 1.1 |
| Kazakhstan | Measurement | 16.8 | 17.5 | 18.1 | 18.8 | 19.5 | 20.2 |  |  | 17.2 | 17.5 | 17.9 | 18.3 | 18.7 | 19.1 |  |
|  | Projection | 17.0 | 17.5 | 18.0 | 18.4 | 18.9 | 19.4 |  |  | 17.1 | 17.4 | 17.7 | 18.0 | 18.3 | 18.6 |  |
|  | Relative error | 1.2 | 0.0 | 0.6 | 2.1 | 3.1 | 4.0 | 1.8 |  | 0.6 | 0.6 | 1.1 | 1.6 | 2.1 | 2.6 | 1.4 |
| Kenya | Measurement | 4.7 | 5.0 | 5.3 | 5.6 | 6.0 | 6.3 |  |  | 13.2 | 13.8 | 14.4 | 15.0 | 15.7 | 16.3 |  |
|  | Projection | 4.9 | 5.2 | 5.7 | 6.1 | 6.7 | 7.2 |  |  | 13.2 | 13.9 | 14.5 | 15.2 | 15.9 | 16.7 |  |
|  | Relative error | 4.3 | 4.0 | 7.5 | 8.9 | 11.7 | 14.3 | 8.5 |  | 0.0 | 0.7 | 0.7 | 1.3 | 1.3 | 2.5 | 1.1 |
| Kiribati | Measurement | 44.6 | 45.8 | 47.1 | 48.3 | 49.5 | 50.7 |  |  | 55.4 | 56.4 | 57.3 | 58.2 | 59.2 | 60.1 |  |
|  | Projection | 44.5 | 46.0 | 47.5 | 49.0 | 50.5 | 52.1 |  |  | 55.4 | 56.6 | 57.8 | 59.1 | 60.3 | 61.5 |  |
|  | Relative error | 0.2 | 0.4 | 0.8 | 1.4 | 2.0 | 2.8 | 1.3 |  | 0.0 | 0.4 | 0.9 | 1.5 | 1.9 | 2.3 | 1.2 |
| Kuwait | Measurement | 42.2 | 42.7 | 43.2 | 43.7 | 44.2 | 44.8 |  |  | 39.0 | 39.2 | 39.3 | 39.5 | 39.7 | 39.9 |  |
|  | Projection | 42.1 | 42.6 | 43.1 | 43.6 | 44.0 | 44.5 |  |  | 39.0 | 39.2 | 39.3 | 39.4 | 39.4 | 39.5 |  |
|  | Relative error | 0.2 | 0.2 | 0.2 | 0.2 | 0.5 | 0.7 | 0.3 |  | 0.0 | 0.0 | 0.0 | 0.3 | 0.8 | 1.0 | 0.4 |
| Kyrgyzstan | Measurement | 12.7 | 13.3 | 14.0 | 14.6 | 15.4 | 16.1 |  |  | 14.0 | 14.3 | 14.8 | 15.2 | 15.7 | 16.1 |  |
|  | Projection | 12.7 | 13.2 | 13.7 | 14.3 | 14.9 | 15.6 |  |  | 13.9 | 14.2 | 14.6 | 14.9 | 15.2 | 15.5 |  |
|  | Relative error | 0.0 | 0.8 | 2.1 | 2.1 | 3.2 | 3.1 | 1.9 |  | 0.7 | 0.7 | 1.4 | 2.0 | 3.2 | 3.7 | 2.0 |
| Lao People's Democratic Republic | Measurement | 10.6 | 11.5 | 12.4 | 13.4 | 14.5 | 15.6 |  |  | 8.5 | 9.1 | 9.7 | 10.3 | 10.9 | 11.6 |  |
|  | Projection | 10.8 | 11.8 | 13.0 | 14.2 | 15.7 | 17.1 |  |  | 8.7 | 9.3 | 10.0 | 10.8 | 11.6 | 12.5 |  |
|  | Relative error | 1.9 | 2.6 | 4.8 | 6.0 | 8.3 | 9.6 | 5.5 |  | 2.4 | 2.2 | 3.1 | 4.9 | 6.4 | 7.8 | 4.5 |
| Latvia | Measurement | 20.9 | 21.6 | 22.3 | 23.0 | 23.8 | 24.4 |  |  | 16.3 | 16.7 | 17.1 | 17.6 | 18.0 | 18.5 |  |
|  | Projection | 21.0 | 21.4 | 21.9 | 22.4 | 22.8 | 23.3 |  |  | 16.2 | 16.5 | 16.8 | 17.0 | 17.3 | 17.5 |  |
|  | Relative error | 0.5 | 0.9 | 1.8 | 2.6 | 4.2 | 4.5 | 2.4 |  | 0.6 | 1.2 | 1.8 | 3.4 | 3.9 | 5.4 | 2.7 |
| Lebanon | Measurement | 33.2 | 33.7 | 34.2 | 34.7 | 35.3 | 35.9 |  |  | 28.8 | 29.0 | 29.2 | 29.4 | 29.7 | 29.9 |  |
|  | Projection | 33.2 | 33.7 | 34.3 | 34.8 | 35.3 | 35.9 |  |  | 29.0 | 29.1 | 29.3 | 29.4 | 29.5 | 29.6 |  |
|  | Relative error | 0.0 | 0.0 | 0.3 | 0.3 | 0.0 | 0.0 | 0.1 |  | 0.7 | 0.3 | 0.3 | 0.0 | 0.7 | 1.0 | 0.5 |
| Lesotho | Measurement | 4.0 | 4.4 | 4.8 | 5.2 | 5.7 | 6.2 |  |  | 18.5 | 19.6 | 20.8 | 22.1 | 23.4 | 24.7 |  |
|  | Projection | 3.9 | 4.2 | 4.6 | 5.0 | 5.5 | 5.9 |  |  | 18.6 | 20.0 | 21.4 | 23.0 | 24.8 | 26.6 |  |
|  | Relative error | 2.5 | 4.5 | 4.2 | 3.8 | 3.5 | 4.8 | 3.9 |  | 0.5 | 2.0 | 2.9 | 4.1 | 6.0 | 7.7 | 3.9 |
| Liberia | Measurement | 4.9 | 5.1 | 5.4 | 5.6 | 5.9 | 6.2 |  |  | 12.3 | 12.7 | 13.1 | 13.5 | 13.9 | 14.3 |  |
|  | Projection | 4.9 | 5.2 | 5.5 | 5.8 | 6.2 | 6.6 |  |  | 12.4 | 12.7 | 13.0 | 13.3 | 13.6 | 13.9 |  |
|  | Relative error | 0.0 | 2.0 | 1.9 | 3.6 | 5.1 | 6.5 | 3.2 |  | 0.8 | 0.0 | 0.8 | 1.5 | 2.2 | 2.8 | 1.4 |
| Libya | Measurement | 30.1 | 30.9 | 31.6 | 32.4 | 33.1 | 33.8 |  |  | 29.3 | 29.8 | 30.2 | 30.6 | 31.1 | 31.5 |  |
|  | Projection | 30.1 | 30.8 | 31.4 | 32.0 | 32.7 | 33.3 |  |  | 29.4 | 29.7 | 30.0 | 30.2 | 30.5 | 30.7 |  |
|  | Relative error | 0.0 | 0.3 | 0.6 | 1.2 | 1.2 | 1.5 | 0.8 |  | 0.3 | 0.3 | 0.7 | 1.3 | 1.9 | 2.5 | 1.2 |
| Lithuania | Measurement | 19.8 | 20.3 | 21.0 | 21.7 | 22.3 | 23.1 |  |  | 15.8 | 16.1 | 16.5 | 16.9 | 17.4 | 17.8 |  |
|  | Projection | 20.0 | 20.4 | 20.8 | 21.2 | 21.6 | 22.0 |  |  | 16.0 | 16.3 | 16.5 | 16.8 | 17.1 | 17.4 |  |
|  | Relative error | 1.0 | 0.5 | 1.0 | 2.3 | 3.1 | 4.8 | 2.1 |  | 1.3 | 1.2 | 0.0 | 0.6 | 1.7 | 2.2 | 1.2 |
| Luxembourg | Measurement | 27.4 | 27.5 | 27.6 | 27.7 | 27.8 | 27.9 |  |  | 23.9 | 23.9 | 24.0 | 24.1 | 24.1 | 24.2 |  |
|  | Projection | 27.5 | 27.7 | 27.8 | 27.9 | 28.0 | 28.1 |  |  | 23.9 | 24.0 | 24.0 | 24.1 | 24.2 | 24.2 |  |
|  | Relative error | 0.4 | 0.7 | 0.7 | 0.7 | 0.7 | 0.7 | 0.7 |  | 0.0 | 0.4 | 0.0 | 0.0 | 0.4 | 0.0 | 0.1 |
| Madagascar | Measurement | 5.9 | 6.3 | 6.7 | 7.1 | 7.5 | 8.0 |  |  | 11.1 | 11.5 | 12.0 | 12.5 | 13.0 | 13.5 |  |
|  | Projection | 6.2 | 6.5 | 7.2 | 7.6 | 8.4 | 8.9 |  |  | 11.1 | 11.6 | 12.1 | 12.5 | 13.0 | 13.5 |  |
|  | Relative error | 5.1 | 3.2 | 7.5 | 7.0 | 12.0 | 11.3 | 7.7 |  | 0.0 | 0.9 | 0.8 | 0.0 | 0.0 | 0.0 | 0.3 |
| Malawi | Measurement | 4.6 | 4.9 | 5.2 | 5.6 | 6.0 | 6.4 |  |  | 11.9 | 12.5 | 13.0 | 13.6 | 14.2 | 14.9 |  |
|  | Projection | 4.6 | 5.0 | 5.4 | 5.8 | 6.2 | 6.8 |  |  | 12.0 | 12.5 | 13.1 | 13.8 | 14.4 | 15.1 |  |
|  | Relative error | 0.0 | 2.0 | 3.8 | 3.6 | 3.3 | 6.2 | 3.2 |  | 0.8 | 0.0 | 0.8 | 1.5 | 1.4 | 1.3 | 1.0 |
| Malaysia | Measurement | 24.5 | 25.6 | 26.7 | 27.8 | 28.9 | 30.0 |  |  | 19.4 | 20.2 | 20.9 | 21.7 | 22.4 | 23.2 |  |
|  | Projection | 24.3 | 25.5 | 26.8 | 28.1 | 29.4 | 30.7 |  |  | 19.5 | 20.3 | 21.2 | 22.0 | 22.9 | 23.9 |  |
|  | Relative error | 0.8 | 0.4 | 0.4 | 1.1 | 1.7 | 2.3 | 1.1 |  | 0.5 | 0.5 | 1.4 | 1.4 | 2.2 | 3.0 | 1.5 |
| Maldives | Measurement | 13.4 | 14.5 | 15.7 | 16.9 | 18.1 | 19.4 |  |  | 10.5 | 11.1 | 11.9 | 12.6 | 13.4 | 14.2 |  |
|  | Projection | 13.5 | 14.4 | 15.3 | 16.2 | 17.2 | 18.2 |  |  | 10.7 | 11.6 | 12.4 | 13.4 | 14.4 | 15.5 |  |
|  | Relative error | 0.7 | 0.7 | 2.5 | 4.1 | 5.0 | 6.2 | 3.2 |  | 1.9 | 4.5 | 4.2 | 6.3 | 7.5 | 9.2 | 5.6 |
| Mali | Measurement | 4.9 | 5.3 | 5.6 | 6.0 | 6.4 | 6.9 |  |  | 12.1 | 12.6 | 13.1 | 13.7 | 14.2 | 14.8 |  |
|  | Projection | 5.1 | 5.5 | 5.9 | 6.5 | 7.0 | 7.5 |  |  | 12.2 | 12.8 | 13.4 | 14.0 | 14.7 | 15.4 |  |
|  | Relative error | 4.1 | 3.8 | 5.4 | 8.3 | 9.4 | 8.7 | 6.6 |  | 0.8 | 1.6 | 2.3 | 2.2 | 3.5 | 4.1 | 2.4 |
| Malta | Measurement | 39.0 | 39.1 | 39.2 | 39.3 | 39.4 | 39.5 |  |  | 33.0 | 33.1 | 33.2 | 33.4 | 33.5 | 33.7 |  |
|  | Projection | 39.0 | 39.2 | 39.3 | 39.4 | 39.5 | 39.6 |  |  | 33.0 | 33.2 | 33.3 | 33.5 | 33.7 | 33.9 |  |
|  | Relative error | 0.0 | 0.3 | 0.3 | 0.3 | 0.3 | 0.3 | 0.3 |  | 0.0 | 0.3 | 0.3 | 0.3 | 0.6 | 0.6 | 0.4 |
| Marshall Islands | Measurement | 50.0 | 51.1 | 52.1 | 53.1 | 54.1 | 55.1 |  |  | 59.7 | 60.5 | 61.3 | 62.0 | 62.8 | 63.5 |  |
|  | Projection | 49.9 | 51.1 | 52.2 | 53.4 | 54.6 | 55.8 |  |  | 59.9 | 60.8 | 61.8 | 62.7 | 63.7 | 64.6 |  |
|  | Relative error | 0.2 | 0.0 | 0.2 | 0.6 | 0.9 | 1.3 | 0.5 |  | 0.3 | 0.5 | 0.8 | 1.1 | 1.4 | 1.7 | 1.0 |
| Mauritania | Measurement | 7.1 | 7.5 | 7.9 | 8.3 | 8.7 | 9.1 |  |  | 15.2 | 15.7 | 16.1 | 16.6 | 17.0 | 17.5 |  |
|  | Projection | 7.1 | 7.5 | 7.8 | 8.2 | 8.6 | 9.0 |  |  | 15.2 | 15.7 | 16.3 | 16.8 | 17.4 | 17.9 |  |
|  | Relative error | 0.0 | 0.0 | 1.3 | 1.2 | 1.1 | 1.1 | 0.8 |  | 0.0 | 0.0 | 1.2 | 1.2 | 2.4 | 2.3 | 1.2 |
| Mauritius | Measurement | 8.7 | 9.1 | 9.6 | 10.0 | 10.5 | 11.0 |  |  | 15.6 | 16.1 | 16.6 | 17.2 | 17.7 | 18.3 |  |
|  | Projection | 8.7 | 9.2 | 9.6 | 10.1 | 10.6 | 11.1 |  |  | 15.8 | 16.3 | 17.0 | 17.6 | 18.3 | 19.0 |  |
|  | Relative error | 0.0 | 1.1 | 0.0 | 1.0 | 1.0 | 0.9 | 0.7 |  | 1.3 | 1.2 | 2.4 | 2.3 | 3.4 | 3.8 | 2.4 |
| Mexico | Measurement | 33.3 | 33.8 | 34.3 | 34.7 | 35.2 | 35.7 |  |  | 32.7 | 33.2 | 33.7 | 34.2 | 34.7 | 35.1 |  |
|  | Projection | 33.3 | 33.9 | 34.5 | 35.1 | 35.7 | 36.3 |  |  | 32.8 | 33.3 | 33.8 | 34.4 | 34.9 | 35.4 |  |
|  | Relative error | 0.0 | 0.3 | 0.6 | 1.2 | 1.4 | 1.7 | 0.9 |  | 0.3 | 0.3 | 0.3 | 0.6 | 0.6 | 0.9 | 0.5 |
| Micronesia (Federated States of) | Measurement | 39.3 | 40.7 | 42.1 | 43.6 | 45.0 | 46.5 |  |  | 50.5 | 51.7 | 52.9 | 54.1 | 55.2 | 56.4 |  |
|  | Projection | 39.2 | 40.6 | 42.0 | 43.5 | 45.0 | 46.6 |  |  | 50.4 | 51.6 | 52.9 | 54.2 | 55.6 | 56.9 |  |
|  | Relative error | 0.3 | 0.2 | 0.2 | 0.2 | 0.0 | 0.2 | 0.2 |  | 0.2 | 0.2 | 0.0 | 0.2 | 0.7 | 0.9 | 0.4 |
| Mongolia | Measurement | 13.7 | 14.3 | 14.8 | 15.5 | 16.1 | 16.7 |  |  | 17.1 | 17.5 | 17.8 | 18.2 | 18.5 | 18.9 |  |
|  | Projection | 13.8 | 14.2 | 14.7 | 15.1 | 15.6 | 16.1 |  |  | 17.1 | 17.5 | 17.8 | 18.1 | 18.4 | 18.7 |  |
|  | Relative error | 0.7 | 0.7 | 0.7 | 2.6 | 3.1 | 3.6 | 1.9 |  | 0.0 | 0.0 | 0.0 | 0.5 | 0.5 | 1.1 | 0.4 |
| Montenegro | Measurement | 25.5 | 26.3 | 27.2 | 28.0 | 28.9 | 29.8 |  |  | 17.0 | 17.4 | 17.9 | 18.4 | 19.0 | 19.5 |  |
|  | Projection | 25.4 | 26.4 | 27.3 | 28.3 | 29.3 | 30.3 |  |  | 17.0 | 17.5 | 18.1 | 18.7 | 19.3 | 19.9 |  |
|  | Relative error | 0.4 | 0.4 | 0.4 | 1.1 | 1.4 | 1.7 | 0.9 |  | 0.0 | 0.6 | 1.1 | 1.6 | 1.6 | 2.1 | 1.2 |
| Morocco | Measurement | 22.6 | 23.4 | 24.3 | 25.1 | 26.0 | 26.9 |  |  | 23.9 | 24.5 | 25.2 | 25.8 | 26.4 | 27.1 |  |
|  | Projection | 22.7 | 23.5 | 24.4 | 25.3 | 26.2 | 27.1 |  |  | 23.9 | 24.5 | 25.1 | 25.7 | 26.3 | 27.0 |  |
|  | Relative error | 0.4 | 0.4 | 0.4 | 0.8 | 0.8 | 0.7 | 0.6 |  | 0.0 | 0.0 | 0.4 | 0.4 | 0.4 | 0.4 | 0.3 |
| Mozambique | Measurement | 6.3 | 6.6 | 6.9 | 7.3 | 7.6 | 8.0 |  |  | 14.7 | 15.2 | 15.7 | 16.2 | 16.7 | 17.2 |  |
|  | Projection | 6.5 | 7.1 | 7.8 | 8.4 | 9.2 | 10.0 |  |  | 14.7 | 15.3 | 15.8 | 16.4 | 16.9 | 17.5 |  |
|  | Relative error | 3.2 | 7.6 | 13.0 | 15.1 | 21.1 | 25.0 | 14.2 |  | 0.0 | 0.7 | 0.6 | 1.2 | 1.2 | 1.7 | 0.9 |
| Myanmar | Measurement | 9.4 | 10.1 | 10.8 | 11.6 | 12.4 | 13.3 |  |  | 7.5 | 8.0 | 8.4 | 8.9 | 9.3 | 9.9 |  |
|  | Projection | 9.4 | 10.1 | 10.9 | 11.8 | 12.7 | 13.7 |  |  | 7.6 | 8.1 | 8.7 | 9.2 | 9.9 | 10.4 |  |
|  | Relative error | 0.0 | 0.0 | 0.9 | 1.7 | 2.4 | 3.0 | 1.3 |  | 1.3 | 1.3 | 3.6 | 3.4 | 6.5 | 5.1 | 3.5 |
| Namibia | Measurement | 6.4 | 7.0 | 7.7 | 8.3 | 9.1 | 9.8 |  |  | 15.7 | 16.7 | 17.7 | 18.7 | 19.8 | 20.8 |  |
|  | Projection | 6.4 | 6.8 | 7.4 | 7.9 | 8.6 | 9.2 |  |  | 15.7 | 16.7 | 17.9 | 19.1 | 20.4 | 21.8 |  |
|  | Relative error | 0.0 | 2.9 | 3.9 | 4.8 | 5.5 | 6.1 | 3.9 |  | 0.0 | 0.0 | 1.1 | 2.1 | 3.0 | 4.8 | 1.8 |
| Nauru | Measurement | 59.0 | 59.5 | 60.1 | 60.7 | 61.3 | 61.8 |  |  | 66.1 | 66.6 | 67.0 | 67.4 | 67.8 | 68.2 |  |
|  | Projection | 58.9 | 59.6 | 60.2 | 60.8 | 61.5 | 62.1 |  |  | 66.1 | 66.6 | 67.0 | 67.5 | 68.0 | 68.4 |  |
|  | Relative error | 0.2 | 0.2 | 0.2 | 0.2 | 0.3 | 0.5 | 0.3 |  | 0.0 | 0.0 | 0.0 | 0.1 | 0.3 | 0.3 | 0.1 |
| Nepal | Measurement | 5.2 | 5.6 | 5.9 | 6.3 | 6.8 | 7.2 |  |  | 5.5 | 5.9 | 6.3 | 6.8 | 7.2 | 7.7 |  |
|  | Projection | 5.2 | 5.5 | 5.8 | 6.2 | 6.5 | 6.9 |  |  | 5.6 | 6.0 | 6.6 | 7.1 | 7.8 | 8.4 |  |
|  | Relative error | 0.0 | 1.8 | 1.7 | 1.6 | 4.4 | 4.2 | 2.3 |  | 1.8 | 1.7 | 4.8 | 4.4 | 8.3 | 9.1 | 5.0 |
| Netherlands | Measurement | 24.3 | 24.6 | 24.8 | 25.1 | 25.3 | 25.4 |  |  | 23.1 | 23.2 | 23.4 | 23.6 | 23.7 | 23.9 |  |
|  | Projection | 24.3 | 24.6 | 25.0 | 25.4 | 25.8 | 26.2 |  |  | 23.0 | 23.3 | 23.6 | 23.9 | 24.2 | 24.4 |  |
|  | Relative error | 0.0 | 0.0 | 0.8 | 1.2 | 2.0 | 3.1 | 1.2 |  | 0.4 | 0.4 | 0.9 | 1.3 | 2.1 | 2.1 | 1.2 |
| New Zealand | Measurement | 37.8 | 38.2 | 38.6 | 39.1 | 39.5 | 40.0 |  |  | 36.9 | 37.3 | 37.7 | 38.1 | 38.5 | 38.9 |  |
|  | Projection | 38.0 | 38.5 | 39.0 | 39.5 | 40.0 | 40.6 |  |  | 37.0 | 37.5 | 37.9 | 38.3 | 38.7 | 39.2 |  |
|  | Relative error | 0.5 | 0.8 | 1.0 | 1.0 | 1.3 | 1.5 | 1.0 |  | 0.3 | 0.5 | 0.5 | 0.5 | 0.5 | 0.8 | 0.5 |
| Nicaragua | Measurement | 23.1 | 23.7 | 24.5 | 25.2 | 25.9 | 26.7 |  |  | 28.7 | 29.3 | 30.0 | 30.6 | 31.3 | 32.0 |  |
|  | Projection | 23.1 | 23.8 | 24.5 | 25.2 | 25.9 | 26.7 |  |  | 28.8 | 29.4 | 30.1 | 30.8 | 31.5 | 32.1 |  |
|  | Relative error | 0.0 | 0.4 | 0.0 | 0.0 | 0.0 | 0.0 | 0.1 |  | 0.3 | 0.3 | 0.3 | 0.7 | 0.6 | 0.3 | 0.4 |
| Niger | Measurement | 3.5 | 3.8 | 4.1 | 4.4 | 4.7 | 5.1 |  |  | 8.9 | 9.4 | 9.9 | 10.3 | 10.9 | 11.4 |  |
|  | Projection | 3.6 | 4.0 | 4.2 | 4.6 | 5.1 | 5.4 |  |  | 9.0 | 9.6 | 10.2 | 10.9 | 11.5 | 12.3 |  |
|  | Relative error | 2.9 | 5.3 | 2.4 | 4.5 | 8.5 | 5.9 | 4.9 |  | 1.1 | 2.1 | 3.0 | 5.8 | 5.5 | 7.9 | 4.2 |
| Nigeria | Measurement | 4.7 | 5.1 | 5.4 | 5.8 | 6.2 | 6.7 |  |  | 8.5 | 8.8 | 9.2 | 9.6 | 9.9 | 10.3 |  |
|  | Projection | 4.8 | 5.2 | 5.6 | 6.1 | 6.6 | 7.1 |  |  | 8.6 | 8.9 | 9.3 | 9.7 | 10.1 | 10.6 |  |
|  | Relative error | 2.1 | 2.0 | 3.7 | 5.2 | 6.5 | 6.0 | 4.3 |  | 1.2 | 1.1 | 1.1 | 1.0 | 2.0 | 2.9 | 1.6 |
| Niue | Measurement | 48.5 | 50.1 | 51.6 | 53.2 | 54.6 | 56.1 |  |  | 56.0 | 57.2 | 58.4 | 59.6 | 60.7 | 61.8 |  |
|  | Projection | 48.2 | 50.0 | 51.7 | 53.4 | 55.2 | 57.1 |  |  | 55.4 | 56.6 | 57.9 | 59.1 | 60.3 | 61.5 |  |
|  | Relative error | 0.6 | 0.2 | 0.2 | 0.4 | 1.1 | 1.8 | 0.7 |  | 1.0 | 1.0 | 0.9 | 0.9 | 0.7 | 0.5 | 0.8 |
| Norway | Measurement | 27.8 | 28.0 | 28.2 | 28.4 | 28.6 | 28.8 |  |  | 25.1 | 25.3 | 25.4 | 25.6 | 25.8 | 25.9 |  |
|  | Projection | 28.0 | 28.2 | 28.5 | 28.7 | 28.9 | 29.0 |  |  | 25.2 | 25.3 | 25.5 | 25.6 | 25.8 | 25.9 |  |
|  | Relative error | 0.7 | 0.7 | 1.1 | 1.1 | 1.0 | 0.7 | 0.9 |  | 0.4 | 0.0 | 0.4 | 0.0 | 0.0 | 0.0 | 0.1 |
| Oman | Measurement | 29.5 | 30.3 | 31.1 | 32.0 | 32.9 | 33.9 |  |  | 27.6 | 28.1 | 28.7 | 29.3 | 29.8 | 30.4 |  |
|  | Projection | 30.0 | 30.8 | 31.6 | 32.4 | 33.2 | 34.0 |  |  | 28.0 | 28.4 | 28.8 | 29.2 | 29.6 | 30.0 |  |
|  | Relative error | 1.7 | 1.7 | 1.6 | 1.3 | 0.9 | 0.3 | 1.3 |  | 1.4 | 1.1 | 0.3 | 0.3 | 0.7 | 1.3 | 0.9 |
| Pakistan | Measurement | 7.5 | 8.0 | 8.5 | 9.0 | 9.6 | 10.1 |  |  | 6.8 | 7.2 | 7.6 | 8.1 | 8.6 | 9.1 |  |
|  | Projection | 7.7 | 8.2 | 8.9 | 9.5 | 10.3 | 10.9 |  |  | 6.8 | 7.2 | 7.5 | 7.9 | 8.3 | 8.7 |  |
|  | Relative error | 2.7 | 2.5 | 4.7 | 5.6 | 7.3 | 7.9 | 5.1 |  | 0.0 | 0.0 | 1.3 | 2.5 | 3.5 | 4.4 | 2.0 |
| Palau | Measurement | 55.8 | 56.8 | 57.8 | 58.7 | 59.6 | 60.5 |  |  | 63.1 | 63.9 | 64.6 | 65.3 | 66.0 | 66.6 |  |
|  | Projection | 55.3 | 56.5 | 57.7 | 58.9 | 60.0 | 61.2 |  |  | 62.8 | 63.7 | 64.6 | 65.4 | 66.3 | 67.1 |  |
|  | Relative error | 0.9 | 0.5 | 0.2 | 0.3 | 0.7 | 1.2 | 0.6 |  | 0.5 | 0.3 | 0.0 | 0.2 | 0.5 | 0.8 | 0.4 |
| Panama | Measurement | 23.9 | 24.5 | 25.1 | 25.7 | 26.3 | 26.9 |  |  | 29.0 | 29.6 | 30.2 | 30.8 | 31.4 | 32.0 |  |
|  | Projection | 24.0 | 24.7 | 25.4 | 26.1 | 26.9 | 27.6 |  |  | 29.1 | 29.8 | 30.5 | 31.2 | 32.0 | 32.7 |  |
|  | Relative error | 0.4 | 0.8 | 1.2 | 1.6 | 2.3 | 2.6 | 1.5 |  | 0.3 | 0.7 | 1.0 | 1.3 | 1.9 | 2.2 | 1.2 |
| Papua New Guinea | Measurement | 21.2 | 22.1 | 23.0 | 24.0 | 25.0 | 26.0 |  |  | 33.3 | 34.2 | 35.1 | 35.9 | 36.8 | 37.8 |  |
|  | Projection | 21.3 | 22.2 | 23.1 | 24.0 | 25.0 | 25.9 |  |  | 33.4 | 34.3 | 35.2 | 36.1 | 37.0 | 37.9 |  |
|  | Relative error | 0.5 | 0.5 | 0.4 | 0.0 | 0.0 | 0.4 | 0.3 |  | 0.3 | 0.3 | 0.3 | 0.6 | 0.5 | 0.3 | 0.4 |
| Paraguay | Measurement | 24.5 | 25.3 | 26.2 | 27.1 | 28.0 | 28.9 |  |  | 23.8 | 24.4 | 25.0 | 25.7 | 26.3 | 26.9 |  |
|  | Projection | 24.5 | 25.4 | 26.3 | 27.3 | 28.2 | 29.2 |  |  | 23.8 | 24.5 | 25.1 | 25.8 | 26.4 | 27.1 |  |
|  | Relative error | 0.0 | 0.4 | 0.4 | 0.7 | 0.7 | 1.0 | 0.5 |  | 0.0 | 0.4 | 0.4 | 0.4 | 0.4 | 0.7 | 0.4 |
| Peru | Measurement | 23.5 | 24.0 | 24.5 | 25.1 | 25.6 | 26.2 |  |  | 25.8 | 26.2 | 26.7 | 27.1 | 27.5 | 27.9 |  |
|  | Projection | 23.5 | 24.0 | 24.5 | 25.0 | 25.6 | 26.1 |  |  | 25.8 | 26.2 | 26.6 | 27.1 | 27.5 | 27.9 |  |
|  | Relative error | 0.0 | 0.0 | 0.0 | 0.4 | 0.0 | 0.4 | 0.1 |  | 0.0 | 0.0 | 0.4 | 0.0 | 0.0 | 0.0 | 0.1 |
| Philippines | Measurement | 11.6 | 12.1 | 12.7 | 13.3 | 14.0 | 14.7 |  |  | 9.0 | 9.3 | 9.7 | 10.1 | 10.5 | 10.9 |  |
|  | Projection | 11.7 | 12.2 | 12.8 | 13.3 | 13.9 | 14.4 |  |  | 9.0 | 9.4 | 9.7 | 10.0 | 10.4 | 10.7 |  |
|  | Relative error | 0.9 | 0.8 | 0.8 | 0.0 | 0.7 | 2.0 | 0.9 |  | 0.0 | 1.1 | 0.0 | 1.0 | 1.0 | 1.8 | 0.8 |
| Poland | Measurement | 25.8 | 26.8 | 27.8 | 28.9 | 29.9 | 31.0 |  |  | 16.9 | 17.4 | 18.1 | 18.7 | 19.4 | 20.0 |  |
|  | Projection | 26.0 | 26.8 | 27.6 | 28.4 | 29.2 | 30.1 |  |  | 16.9 | 17.4 | 17.9 | 18.4 | 18.9 | 19.4 |  |
|  | Relative error | 0.8 | 0.0 | 0.7 | 1.7 | 2.3 | 2.9 | 1.4 |  | 0.0 | 0.0 | 1.1 | 1.6 | 2.6 | 3.0 | 1.4 |
| Portugal | Measurement | 31.9 | 32.2 | 32.4 | 32.7 | 32.9 | 33.1 |  |  | 30.1 | 30.4 | 30.8 | 31.1 | 31.4 | 31.7 |  |
|  | Projection | 31.7 | 32.3 | 32.9 | 33.4 | 34.0 | 34.5 |  |  | 29.9 | 30.5 | 31.1 | 31.7 | 32.2 | 32.8 |  |
|  | Relative error | 0.6 | 0.3 | 1.5 | 2.1 | 3.3 | 4.2 | 2.0 |  | 0.7 | 0.3 | 1.0 | 1.9 | 2.5 | 3.5 | 1.7 |
| Qatar | Measurement | 38.7 | 39.2 | 39.7 | 40.2 | 40.7 | 41.3 |  |  | 34.8 | 34.9 | 35.0 | 35.2 | 35.3 | 35.4 |  |
|  | Projection | 38.8 | 39.4 | 40.0 | 40.6 | 41.2 | 41.8 |  |  | 34.7 | 35.0 | 35.3 | 35.6 | 35.8 | 36.1 |  |
|  | Relative error | 0.3 | 0.5 | 0.8 | 1.0 | 1.2 | 1.2 | 0.8 |  | 0.3 | 0.3 | 0.9 | 1.1 | 1.4 | 2.0 | 1.0 |
| Republic of Korea | Measurement | 29.8 | 30.3 | 30.8 | 31.5 | 32.1 | 32.8 |  |  | 18.7 | 19.0 | 19.3 | 19.7 | 20.1 | 20.5 |  |
|  | Projection | 30.1 | 30.6 | 31.2 | 31.8 | 32.4 | 32.9 |  |  | 19.0 | 19.2 | 19.5 | 19.8 | 20.0 | 20.3 |  |
|  | Relative error | 1.0 | 1.0 | 1.3 | 1.0 | 0.9 | 0.3 | 0.9 |  | 1.6 | 1.1 | 1.0 | 0.5 | 0.5 | 1.0 | 1.0 |
| Republic of Moldova | Measurement | 15.5 | 16.2 | 17.0 | 17.8 | 18.6 | 19.4 |  |  | 12.8 | 13.3 | 13.8 | 14.3 | 14.9 | 15.4 |  |
|  | Projection | 15.8 | 16.3 | 16.8 | 17.3 | 17.9 | 18.4 |  |  | 12.8 | 13.1 | 13.5 | 13.9 | 14.3 | 14.6 |  |
|  | Relative error | 1.9 | 0.6 | 1.2 | 2.8 | 3.8 | 5.2 | 2.6 |  | 0.0 | 1.5 | 2.2 | 2.8 | 4.0 | 5.2 | 2.6 |
| Republic of North Macedonia | Measurement | 25.7 | 26.6 | 27.6 | 28.6 | 29.6 | 30.6 |  |  | 17.5 | 18.1 | 18.8 | 19.4 | 20.1 | 20.8 |  |
|  | Projection | 25.9 | 26.7 | 27.5 | 28.3 | 29.1 | 29.9 |  |  | 17.6 | 18.1 | 18.6 | 19.2 | 19.7 | 20.2 |  |
|  | Relative error | 0.8 | 0.4 | 0.4 | 1.0 | 1.7 | 2.3 | 1.1 |  | 0.6 | 0.0 | 1.1 | 1.0 | 2.0 | 2.9 | 1.3 |
| Romania | Measurement | 23.7 | 24.7 | 25.8 | 26.9 | 28.0 | 29.1 |  |  | 16.5 | 17.2 | 17.8 | 18.4 | 19.0 | 19.7 |  |
|  | Projection | 23.7 | 24.5 | 25.4 | 26.3 | 27.3 | 28.2 |  |  | 16.5 | 17.0 | 17.5 | 18.1 | 18.6 | 19.2 |  |
|  | Relative error | 0.0 | 0.8 | 1.6 | 2.2 | 2.5 | 3.1 | 1.7 |  | 0.0 | 1.2 | 1.7 | 1.6 | 2.1 | 2.5 | 1.5 |
| Russian Federation | Measurement | 20.4 | 21.2 | 22.0 | 22.8 | 23.6 | 24.3 |  |  | 15.7 | 16.2 | 16.7 | 17.2 | 17.6 | 18.1 |  |
|  | Projection | 22.1 | 23.0 | 23.9 | 24.8 | 25.9 | 27.0 |  |  | 15.7 | 16.0 | 16.2 | 16.5 | 16.8 | 17.1 |  |
|  | Relative error | 8.3 | 8.5 | 8.6 | 8.8 | 9.7 | 11.1 | 9.2 |  | 0.0 | 1.2 | 3.0 | 4.1 | 4.5 | 5.5 | 3.1 |
| Rwanda | Measurement | 4.0 | 4.2 | 4.4 | 4.7 | 4.9 | 5.2 |  |  | 13.7 | 14.3 | 14.9 | 15.6 | 16.2 | 16.9 |  |
|  | Projection | 4.0 | 4.2 | 4.5 | 4.7 | 5.0 | 5.3 |  |  | 13.6 | 14.3 | 15.0 | 15.7 | 16.5 | 17.2 |  |
|  | Relative error | 0.0 | 0.0 | 2.3 | 0.0 | 2.0 | 1.9 | 1.0 |  | 0.7 | 0.0 | 0.7 | 0.6 | 1.9 | 1.8 | 1.0 |
| Saint Kitts and Nevis | Measurement | 22.3 | 23.4 | 24.4 | 25.5 | 26.6 | 27.7 |  |  | 23.7 | 24.5 | 25.4 | 26.3 | 27.1 | 28.1 |  |
|  | Projection | 22.4 | 23.5 | 24.7 | 25.9 | 27.2 | 28.5 |  |  | 23.7 | 24.6 | 25.5 | 26.4 | 27.4 | 28.5 |  |
|  | Relative error | 0.4 | 0.4 | 1.2 | 1.6 | 2.3 | 2.9 | 1.5 |  | 0.0 | 0.4 | 0.4 | 0.4 | 1.1 | 1.4 | 0.6 |
| Saint Lucia | Measurement | 17.6 | 18.4 | 19.3 | 20.3 | 21.3 | 22.4 |  |  | 20.3 | 21.0 | 21.8 | 22.7 | 23.6 | 24.5 |  |
|  | Projection | 18.0 | 18.7 | 19.4 | 20.2 | 21.0 | 21.8 |  |  | 20.5 | 21.2 | 21.9 | 22.6 | 23.3 | 24.0 |  |
|  | Relative error | 2.3 | 1.6 | 0.5 | 0.5 | 1.4 | 2.7 | 1.5 |  | 1.0 | 1.0 | 0.5 | 0.4 | 1.3 | 2.0 | 1.0 |
| Saint Vincent and the Grenadines | Measurement | 23.8 | 24.8 | 25.7 | 26.7 | 27.7 | 28.8 |  |  | 25.3 | 26.0 | 26.8 | 27.5 | 28.3 | 29.1 |  |
|  | Projection | 23.9 | 24.9 | 25.8 | 26.8 | 27.9 | 28.9 |  |  | 25.3 | 26.1 | 26.9 | 27.7 | 28.5 | 29.3 |  |
|  | Relative error | 0.4 | 0.4 | 0.4 | 0.4 | 0.7 | 0.3 | 0.4 |  | 0.0 | 0.4 | 0.4 | 0.7 | 0.7 | 0.7 | 0.5 |
| Samoa | Measurement | 40.5 | 41.9 | 43.4 | 44.8 | 46.3 | 47.7 |  |  | 51.6 | 52.9 | 54.1 | 55.4 | 56.6 | 57.8 |  |
|  | Projection | 40.4 | 41.9 | 43.5 | 45.1 | 46.8 | 48.4 |  |  | 51.6 | 53.0 | 54.5 | 56.0 | 57.5 | 59.0 |  |
|  | Relative error | 0.2 | 0.0 | 0.2 | 0.7 | 1.1 | 1.5 | 0.6 |  | 0.0 | 0.2 | 0.7 | 1.1 | 1.6 | 2.1 | 1.0 |
| Sao Tome and Principe | Measurement | 7.6 | 8.0 | 8.4 | 8.8 | 9.3 | 9.7 |  |  | 14.7 | 15.1 | 15.5 | 15.9 | 16.3 | 16.8 |  |
|  | Projection | 7.9 | 8.3 | 9.0 | 9.5 | 10.4 | 11.0 |  |  | 14.6 | 15.1 | 15.6 | 16.1 | 16.6 | 17.2 |  |
|  | Relative error | 3.9 | 3.8 | 7.1 | 8.0 | 11.8 | 13.4 | 8.0 |  | 0.7 | 0.0 | 0.6 | 1.3 | 1.8 | 2.4 | 1.1 |
| Saudi Arabia | Measurement | 34.1 | 35.0 | 35.8 | 36.7 | 37.5 | 38.3 |  |  | 30.1 | 30.6 | 31.0 | 31.4 | 31.9 | 32.3 |  |
|  | Projection | 34.1 | 34.9 | 35.7 | 36.5 | 37.3 | 38.1 |  |  | 30.2 | 30.6 | 31.0 | 31.3 | 31.6 | 32.0 |  |
|  | Relative error | 0.0 | 0.3 | 0.3 | 0.5 | 0.5 | 0.5 | 0.4 |  | 0.3 | 0.0 | 0.0 | 0.3 | 0.9 | 0.9 | 0.4 |
| Senegal | Measurement | 4.4 | 4.7 | 4.9 | 5.2 | 5.5 | 5.8 |  |  | 11.4 | 11.8 | 12.2 | 12.6 | 13.0 | 13.5 |  |
|  | Projection | 4.5 | 4.8 | 5.1 | 5.4 | 5.7 | 6.1 |  |  | 11.4 | 11.8 | 12.1 | 12.5 | 12.9 | 13.4 |  |
|  | Relative error | 2.3 | 2.1 | 4.1 | 3.8 | 3.6 | 5.2 | 3.5 |  | 0.0 | 0.0 | 0.8 | 0.8 | 0.8 | 0.7 | 0.5 |
| Serbia | Measurement | 27.5 | 28.5 | 29.5 | 30.5 | 31.5 | 32.5 |  |  | 18.4 | 19.1 | 19.8 | 20.5 | 21.2 | 21.9 |  |
|  | Projection | 27.5 | 28.4 | 29.4 | 30.4 | 31.5 | 32.5 |  |  | 18.4 | 19.1 | 19.7 | 20.4 | 21.1 | 21.8 |  |
|  | Relative error | 0.0 | 0.4 | 0.3 | 0.3 | 0.0 | 0.0 | 0.2 |  | 0.0 | 0.0 | 0.5 | 0.5 | 0.5 | 0.5 | 0.3 |
| Seychelles | Measurement | 17.4 | 18.1 | 18.8 | 19.5 | 20.3 | 21.1 |  |  | 21.2 | 21.8 | 22.4 | 23.0 | 23.7 | 24.3 |  |
|  | Projection | 17.4 | 18.1 | 18.8 | 19.5 | 20.3 | 21.1 |  |  | 21.2 | 21.8 | 22.4 | 23.0 | 23.7 | 24.3 |  |
|  | Relative error | 0.0 | 0.0 | 0.0 | 0.0 | 0.0 | 0.0 | 0.0 |  | 0.0 | 0.0 | 0.0 | 0.0 | 0.0 | 0.0 | 0.0 |
| Sierra Leone | Measurement | 4.8 | 5.1 | 5.4 | 5.7 | 6.0 | 6.4 |  |  | 12.8 | 13.2 | 13.7 | 14.2 | 14.7 | 15.2 |  |
|  | Projection | 4.9 | 5.2 | 5.6 | 5.9 | 6.4 | 6.8 |  |  | 12.7 | 13.2 | 13.7 | 14.2 | 14.7 | 15.2 |  |
|  | Relative error | 2.1 | 2.0 | 3.7 | 3.5 | 6.7 | 6.2 | 4.0 |  | 0.8 | 0.0 | 0.0 | 0.0 | 0.0 | 0.0 | 0.1 |
| Singapore | Measurement | 26.3 | 26.3 | 26.4 | 26.5 | 26.6 | 26.7 |  |  | 17.7 | 17.7 | 17.7 | 17.7 | 17.8 | 17.9 |  |
|  | Projection | 26.4 | 26.4 | 26.5 | 26.6 | 26.6 | 26.7 |  |  | 17.6 | 17.6 | 17.6 | 17.6 | 17.6 | 17.6 |  |
|  | Relative error | 0.4 | 0.4 | 0.4 | 0.4 | 0.0 | 0.0 | 0.3 |  | 0.6 | 0.6 | 0.6 | 0.6 | 1.1 | 1.7 | 0.9 |
| Slovakia | Measurement | 22.5 | 23.5 | 24.5 | 25.6 | 26.7 | 27.8 |  |  | 15.3 | 15.9 | 16.6 | 17.2 | 17.9 | 18.6 |  |
|  | Projection | 22.7 | 23.5 | 24.3 | 25.1 | 25.9 | 26.7 |  |  | 15.4 | 15.9 | 16.4 | 16.9 | 17.4 | 17.9 |  |
|  | Relative error | 0.9 | 0.0 | 0.8 | 2.0 | 3.0 | 4.0 | 1.8 |  | 0.7 | 0.0 | 1.2 | 1.7 | 2.8 | 3.8 | 1.7 |
| Slovenia | Measurement | 25.6 | 26.6 | 27.7 | 28.8 | 29.9 | 31.0 |  |  | 19.3 | 20.0 | 20.8 | 21.6 | 22.4 | 23.3 |  |
|  | Projection | 25.6 | 26.5 | 27.4 | 28.4 | 29.4 | 30.4 |  |  | 19.3 | 19.9 | 20.6 | 21.3 | 22.0 | 22.7 |  |
|  | Relative error | 0.0 | 0.4 | 1.1 | 1.4 | 1.7 | 1.9 | 1.1 |  | 0.0 | 0.5 | 1.0 | 1.4 | 1.8 | 2.6 | 1.2 |
| Solomon Islands | Measurement | 13.4 | 14.0 | 14.7 | 15.3 | 16.0 | 16.8 |  |  | 26.4 | 27.0 | 27.7 | 28.5 | 29.2 | 29.9 |  |
|  | Projection | 13.6 | 14.1 | 14.7 | 15.3 | 15.9 | 16.6 |  |  | 26.5 | 27.2 | 27.9 | 28.6 | 29.3 | 30.1 |  |
|  | Relative error | 1.5 | 0.7 | 0.0 | 0.0 | 0.6 | 1.2 | 0.7 |  | 0.4 | 0.7 | 0.7 | 0.4 | 0.3 | 0.7 | 0.5 |
| Somalia | Measurement | 6.6 | 6.9 | 7.3 | 7.7 | 8.1 | 8.6 |  |  | 14.5 | 15.0 | 15.6 | 16.1 | 16.7 | 17.3 |  |
|  | Projection | 6.7 | 7.1 | 7.7 | 8.2 | 8.9 | 9.5 |  |  | 14.4 | 15.0 | 15.5 | 16.1 | 16.7 | 17.3 |  |
|  | Relative error | 1.5 | 2.9 | 5.5 | 6.5 | 9.9 | 10.5 | 6.1 |  | 0.7 | 0.0 | 0.6 | 0.0 | 0.0 | 0.0 | 0.2 |
| South Africa | Measurement | 12.5 | 13.9 | 15.4 | 17.0 | 18.6 | 20.3 |  |  | 21.4 | 22.9 | 24.4 | 26.0 | 27.6 | 29.2 |  |
|  | Projection | 14.5 | 16.1 | 17.7 | 19.5 | 21.4 | 23.4 |  |  | 21.2 | 22.6 | 23.9 | 25.4 | 26.9 | 28.4 |  |
|  | Relative error | 16.0 | 15.8 | 14.9 | 14.7 | 15.1 | 15.3 | 15.3 |  | 0.9 | 1.3 | 2.0 | 2.3 | 2.5 | 2.7 | 2.0 |
| Spain | Measurement | 35.6 | 36.0 | 36.3 | 36.5 | 36.8 | 37.0 |  |  | 29.1 | 29.5 | 29.9 | 30.3 | 30.6 | 31.0 |  |
|  | Projection | 35.7 | 36.0 | 36.3 | 36.6 | 36.9 | 37.1 |  |  | 29.0 | 29.3 | 29.7 | 30.1 | 30.4 | 30.8 |  |
|  | Relative error | 0.3 | 0.0 | 0.0 | 0.3 | 0.3 | 0.3 | 0.2 |  | 0.3 | 0.7 | 0.7 | 0.7 | 0.7 | 0.6 | 0.6 |
| Sri Lanka | Measurement | 9.6 | 10.4 | 11.2 | 12.1 | 13.0 | 14.0 |  |  | 8.5 | 9.1 | 9.7 | 10.3 | 11.0 | 11.8 |  |
|  | Projection | 9.6 | 10.4 | 11.2 | 12.2 | 13.1 | 14.2 |  |  | 8.5 | 9.1 | 9.7 | 10.3 | 11.1 | 11.8 |  |
|  | Relative error | 0.0 | 0.0 | 0.0 | 0.8 | 0.8 | 1.4 | 0.5 |  | 0.0 | 0.0 | 0.0 | 0.0 | 0.9 | 0.0 | 0.2 |
| Sudan (former) | Measurement | 6.2 | 6.6 | 6.9 | 7.3 | 7.7 | 8.1 |  |  | 14.0 | 14.5 | 15.1 | 15.6 | 16.2 | 16.8 |  |
|  | Projection | 6.4 | 6.7 | 7.0 | 7.4 | 7.7 | 8.1 |  |  | 14.1 | 14.6 | 15.2 | 15.7 | 16.3 | 16.9 |  |
|  | Relative error | 3.2 | 1.5 | 1.4 | 1.4 | 0.0 | 0.0 | 1.3 |  | 0.7 | 0.7 | 0.7 | 0.6 | 0.6 | 0.6 | 0.7 |
| Suriname | Measurement | 28.7 | 29.4 | 30.1 | 30.7 | 31.4 | 32.1 |  |  | 27.8 | 28.3 | 28.7 | 29.1 | 29.6 | 30.0 |  |
|  | Projection | 28.5 | 29.2 | 29.8 | 30.5 | 31.2 | 31.8 |  |  | 27.8 | 28.2 | 28.5 | 28.9 | 29.3 | 29.7 |  |
|  | Relative error | 0.7 | 0.7 | 1.0 | 0.7 | 0.6 | 0.9 | 0.8 |  | 0.0 | 0.4 | 0.7 | 0.7 | 1.0 | 1.0 | 0.6 |
| Sweden | Measurement | 24.0 | 24.2 | 24.5 | 24.8 | 25.0 | 25.2 |  |  | 21.2 | 21.4 | 21.6 | 21.8 | 22.0 | 22.1 |  |
|  | Projection | 24.4 | 24.5 | 24.6 | 24.7 | 24.8 | 24.9 |  |  | 21.5 | 21.6 | 21.6 | 21.7 | 21.8 | 21.8 |  |
|  | Relative error | 1.7 | 1.2 | 0.4 | 0.4 | 0.8 | 1.2 | 1.0 |  | 1.4 | 0.9 | 0.0 | 0.5 | 0.9 | 1.4 | 0.9 |
| Switzerland | Measurement | 22.2 | 22.3 | 22.4 | 22.5 | 22.6 | 22.8 |  |  | 20.0 | 20.1 | 20.2 | 20.4 | 20.5 | 20.7 |  |
|  | Projection | 22.6 | 22.7 | 22.7 | 22.7 | 22.8 | 22.7 |  |  | 20.2 | 20.3 | 20.3 | 20.3 | 20.3 | 20.3 |  |
|  | Relative error | 1.8 | 1.8 | 1.3 | 0.9 | 0.9 | 0.4 | 1.2 |  | 1.0 | 1.0 | 0.5 | 0.5 | 1.0 | 1.9 | 1.0 |
| Syrian Arab Republic | Measurement | 24.4 | 25.3 | 26.2 | 27.1 | 28.0 | 28.9 |  |  | 24.7 | 25.3 | 25.9 | 26.5 | 27.2 | 27.8 |  |
|  | Projection | 24.3 | 25.2 | 26.2 | 27.2 | 28.2 | 29.3 |  |  | 24.7 | 25.4 | 26.1 | 26.8 | 27.5 | 28.2 |  |
|  | Relative error | 0.4 | 0.4 | 0.0 | 0.4 | 0.7 | 1.4 | 0.6 |  | 0.0 | 0.4 | 0.8 | 1.1 | 1.1 | 1.4 | 0.8 |
| Tajikistan | Measurement | 11.1 | 11.6 | 12.2 | 12.8 | 13.4 | 14.2 |  |  | 13.0 | 13.4 | 13.9 | 14.3 | 14.8 | 15.4 |  |
|  | Projection | 10.9 | 11.3 | 11.8 | 12.3 | 12.7 | 13.3 |  |  | 12.9 | 13.3 | 13.7 | 14.1 | 14.5 | 14.9 |  |
|  | Relative error | 1.8 | 2.6 | 3.3 | 3.9 | 5.2 | 6.3 | 3.9 |  | 0.8 | 0.7 | 1.4 | 1.4 | 2.0 | 3.2 | 1.6 |
| Thailand | Measurement | 18.9 | 20.1 | 21.3 | 22.5 | 23.7 | 24.9 |  |  | 15.1 | 15.9 | 16.8 | 17.6 | 18.4 | 19.2 |  |
|  | Projection | 19.1 | 20.4 | 21.9 | 23.5 | 25.2 | 27.2 |  |  | 15.4 | 16.5 | 17.7 | 19.0 | 20.3 | 21.7 |  |
|  | Relative error | 1.1 | 1.5 | 2.8 | 4.4 | 6.3 | 9.2 | 4.2 |  | 2.0 | 3.8 | 5.4 | 8.0 | 10.3 | 13.0 | 7.1 |
| Timor-Leste | Measurement | 10.0 | 10.7 | 11.5 | 12.3 | 13.2 | 14.1 |  |  | 8.0 | 8.5 | 9.0 | 9.6 | 10.1 | 10.8 |  |
|  | Projection | 10.4 | 11.2 | 12.4 | 13.5 | 14.8 | 16.1 |  |  | 8.2 | 8.8 | 9.4 | 10.0 | 10.7 | 11.5 |  |
|  | Relative error | 4.0 | 4.7 | 7.8 | 9.8 | 12.1 | 14.2 | 8.8 |  | 2.5 | 3.5 | 4.4 | 4.2 | 5.9 | 6.5 | 4.5 |
| Togo | Measurement | 4.8 | 5.1 | 5.5 | 5.8 | 6.2 | 6.6 |  |  | 11.7 | 12.1 | 12.6 | 13.1 | 13.6 | 14.1 |  |
|  | Projection | 4.9 | 5.2 | 5.6 | 6.0 | 6.5 | 6.9 |  |  | 11.8 | 12.5 | 13.2 | 13.9 | 14.7 | 15.5 |  |
|  | Relative error | 2.1 | 2.0 | 1.8 | 3.4 | 4.8 | 4.5 | 3.1 |  | 0.9 | 3.3 | 4.8 | 6.1 | 8.1 | 9.9 | 5.5 |
| Tonga | Measurement | 46.2 | 47.6 | 49.1 | 50.5 | 51.9 | 53.2 |  |  | 57.7 | 58.9 | 60.0 | 61.1 | 62.2 | 63.2 |  |
|  | Projection | 46.0 | 47.6 | 49.3 | 51.0 | 52.8 | 54.5 |  |  | 57.6 | 59.1 | 60.5 | 61.9 | 63.3 | 64.8 |  |
|  | Relative error | 0.4 | 0.0 | 0.4 | 1.0 | 1.7 | 2.4 | 1.0 |  | 0.2 | 0.3 | 0.8 | 1.3 | 1.8 | 2.5 | 1.2 |
| Trinidad and Tobago | Measurement | 18.4 | 19.5 | 20.7 | 21.9 | 23.2 | 24.4 |  |  | 20.1 | 21.1 | 22.1 | 23.1 | 24.2 | 25.3 |  |
|  | Projection | 18.4 | 19.6 | 20.9 | 22.2 | 23.7 | 25.2 |  |  | 20.1 | 21.1 | 22.1 | 23.3 | 24.4 | 25.7 |  |
|  | Relative error | 0.0 | 0.5 | 1.0 | 1.4 | 2.2 | 3.3 | 1.4 |  | 0.0 | 0.0 | 0.0 | 0.9 | 0.8 | 1.6 | 0.6 |
| Tunisia | Measurement | 19.9 | 20.7 | 21.5 | 22.4 | 23.2 | 24.1 |  |  | 23.2 | 23.7 | 24.2 | 24.8 | 25.3 | 25.9 |  |
|  | Projection | 20.1 | 20.8 | 21.6 | 22.4 | 23.2 | 24.1 |  |  | 23.3 | 23.9 | 24.4 | 25.0 | 25.5 | 26.1 |  |
|  | Relative error | 1.0 | 0.5 | 0.5 | 0.0 | 0.0 | 0.0 | 0.3 |  | 0.4 | 0.8 | 0.8 | 0.8 | 0.8 | 0.8 | 0.7 |
| Turkey | Measurement | 26.2 | 27.1 | 27.9 | 28.7 | 29.5 | 30.3 |  |  | 26.1 | 26.6 | 27.1 | 27.6 | 28.1 | 28.6 |  |
|  | Projection | 26.1 | 27.0 | 28.0 | 28.9 | 29.9 | 30.8 |  |  | 26.1 | 26.7 | 27.3 | 27.8 | 28.4 | 29.0 |  |
|  | Relative error | 0.4 | 0.4 | 0.4 | 0.7 | 1.4 | 1.7 | 0.8 |  | 0.0 | 0.4 | 0.7 | 0.7 | 1.1 | 1.4 | 0.7 |
| Turkmenistan | Measurement | 14.6 | 15.3 | 15.9 | 16.6 | 17.3 | 18.0 |  |  | 15.9 | 16.3 | 16.8 | 17.2 | 17.6 | 18.0 |  |
|  | Projection | 14.8 | 15.4 | 15.9 | 16.5 | 17.1 | 17.7 |  |  | 16.0 | 16.4 | 16.8 | 17.2 | 17.6 | 18.0 |  |
|  | Relative error | 1.4 | 0.7 | 0.0 | 0.6 | 1.2 | 1.7 | 0.9 |  | 0.6 | 0.6 | 0.0 | 0.0 | 0.0 | 0.0 | 0.2 |
| Tuvalu | Measurement | 47.2 | 48.6 | 50.0 | 51.3 | 52.7 | 53.9 |  |  | 57.9 | 59.0 | 60.1 | 61.1 | 62.1 | 63.0 |  |
|  | Projection | 47.2 | 48.7 | 50.3 | 51.9 | 53.4 | 55.0 |  |  | 58.0 | 59.3 | 60.5 | 61.8 | 63.1 | 64.4 |  |
|  | Relative error | 0.0 | 0.2 | 0.6 | 1.2 | 1.3 | 2.0 | 0.9 |  | 0.2 | 0.5 | 0.7 | 1.1 | 1.6 | 2.2 | 1.1 |
| Uganda | Measurement | 3.4 | 3.6 | 3.8 | 4.1 | 4.4 | 4.7 |  |  | 12.8 | 13.4 | 14.0 | 14.7 | 15.4 | 16.1 |  |
|  | Projection | 3.5 | 3.8 | 4.1 | 4.4 | 4.8 | 5.1 |  |  | 12.8 | 13.4 | 14.0 | 14.6 | 15.2 | 15.9 |  |
|  | Relative error | 2.9 | 5.6 | 7.9 | 7.3 | 9.1 | 8.5 | 6.9 |  | 0.0 | 0.0 | 0.0 | 0.7 | 1.3 | 1.2 | 0.5 |
| Ukraine | Measurement | 20.6 | 21.3 | 22.0 | 22.8 | 23.6 | 24.4 |  |  | 16.0 | 16.4 | 16.8 | 17.3 | 17.8 | 18.2 |  |
|  | Projection | 20.8 | 21.3 | 21.8 | 22.3 | 22.8 | 23.4 |  |  | 15.9 | 16.3 | 16.6 | 16.9 | 17.3 | 17.6 |  |
|  | Relative error | 1.0 | 0.0 | 0.9 | 2.2 | 3.4 | 4.1 | 1.9 |  | 0.6 | 0.6 | 1.2 | 2.3 | 2.8 | 3.3 | 1.8 |
| United Arab Emirates | Measurement | 31.5 | 32.7 | 33.9 | 35.3 | 36.6 | 37.7 |  |  | 30.9 | 31.5 | 32.1 | 32.7 | 33.2 | 33.7 |  |
|  | Projection | 32.6 | 33.3 | 34.0 | 34.8 | 35.5 | 36.3 |  |  | 31.1 | 31.6 | 32.0 | 32.5 | 33.0 | 33.5 |  |
|  | Relative error | 3.5 | 1.8 | 0.3 | 1.4 | 3.0 | 3.7 | 2.3 |  | 0.6 | 0.3 | 0.3 | 0.6 | 0.6 | 0.6 | 0.5 |
| United Kingdom of Great Britain and Northern Ireland | Measurement | 30.2 | 30.3 | 30.4 | 30.6 | 30.7 | 30.8 |  |  | 31.3 | 31.3 | 31.4 | 31.4 | 31.4 | 31.4 |  |
|  | Projection | 30.2 | 30.5 | 30.8 | 31.1 | 31.3 | 31.6 |  |  | 31.4 | 31.6 | 31.7 | 31.8 | 31.9 | 32.0 |  |
|  | Relative error | 0.0 | 0.7 | 1.3 | 1.6 | 2.0 | 2.6 | 1.4 |  | 0.3 | 1.0 | 1.0 | 1.3 | 1.6 | 1.9 | 1.2 |
| United Republic of Tanzania | Measurement | 5.6 | 6.0 | 6.4 | 6.8 | 7.2 | 7.7 |  |  | 13.1 | 13.7 | 14.3 | 15.0 | 15.6 | 16.3 |  |
|  | Projection | 5.8 | 6.1 | 6.6 | 7.1 | 7.7 | 8.3 |  |  | 13.2 | 13.8 | 14.4 | 15.0 | 15.6 | 16.3 |  |
|  | Relative error | 3.6 | 1.7 | 3.1 | 4.4 | 6.9 | 7.8 | 4.6 |  | 0.8 | 0.7 | 0.7 | 0.0 | 0.0 | 0.0 | 0.4 |
| United States of America | Measurement | 42.3 | 42.7 | 43.1 | 43.4 | 43.8 | 44.1 |  |  | 38.4 | 38.6 | 38.8 | 39.0 | 39.2 | 39.4 |  |
|  | Projection | 42.5 | 43.0 | 43.6 | 44.2 | 44.7 | 45.2 |  |  | 38.6 | 39.0 | 39.3 | 39.6 | 39.9 | 40.2 |  |
|  | Relative error | 0.5 | 0.7 | 1.2 | 1.8 | 2.1 | 2.5 | 1.5 |  | 0.5 | 1.0 | 1.3 | 1.5 | 1.8 | 2.0 | 1.4 |
| Uruguay | Measurement | 32.8 | 33.4 | 34.0 | 34.5 | 35.1 | 35.7 |  |  | 29.5 | 29.8 | 30.1 | 30.4 | 30.7 | 31.0 |  |
|  | Projection | 32.8 | 33.3 | 33.9 | 34.4 | 35.0 | 35.6 |  |  | 29.4 | 29.7 | 30.0 | 30.2 | 30.5 | 30.7 |  |
|  | Relative error | 0.0 | 0.3 | 0.3 | 0.3 | 0.3 | 0.3 | 0.3 |  | 0.3 | 0.3 | 0.3 | 0.7 | 0.7 | 1.0 | 0.6 |
| Uzbekistan | Measurement | 13.0 | 13.6 | 14.2 | 14.9 | 15.6 | 16.3 |  |  | 14.5 | 15.0 | 15.4 | 15.9 | 16.4 | 16.9 |  |
|  | Projection | 12.9 | 13.5 | 14.1 | 14.7 | 15.3 | 15.9 |  |  | 14.7 | 15.1 | 15.4 | 15.8 | 16.1 | 16.5 |  |
|  | Relative error | 0.8 | 0.7 | 0.7 | 1.3 | 1.9 | 2.5 | 1.3 |  | 1.4 | 0.7 | 0.0 | 0.6 | 1.8 | 2.4 | 1.2 |
| Vanuatu | Measurement | 20.4 | 21.2 | 22.0 | 22.8 | 23.7 | 24.6 |  |  | 33.2 | 34.0 | 34.8 | 35.6 | 36.4 | 37.2 |  |
|  | Projection | 20.3 | 21.2 | 22.0 | 22.9 | 23.8 | 24.7 |  |  | 33.3 | 34.2 | 35.0 | 35.8 | 36.7 | 37.6 |  |
|  | Relative error | 0.5 | 0.0 | 0.0 | 0.4 | 0.4 | 0.4 | 0.3 |  | 0.3 | 0.6 | 0.6 | 0.6 | 0.8 | 1.1 | 0.7 |
| Venezuela (Bolivarian Republic of) | Measurement | 31.1 | 31.5 | 31.9 | 32.3 | 32.7 | 33.1 |  |  | 33.3 | 33.7 | 34.0 | 34.4 | 34.7 | 35.1 |  |
|  | Projection | 31.2 | 31.6 | 32.0 | 32.4 | 32.7 | 33.1 |  |  | 33.4 | 33.7 | 34.1 | 34.4 | 34.6 | 34.9 |  |
|  | Relative error | 0.3 | 0.3 | 0.3 | 0.3 | 0.0 | 0.0 | 0.2 |  | 0.3 | 0.0 | 0.3 | 0.0 | 0.3 | 0.6 | 0.3 |
| Viet Nam | Measurement | 6.8 | 7.6 | 8.5 | 9.5 | 10.6 | 11.7 |  |  | 5.2 | 5.6 | 6.1 | 6.6 | 7.1 | 7.6 |  |
|  | Projection | 6.7 | 7.5 | 8.3 | 9.1 | 10.1 | 11.2 |  |  | 5.1 | 5.4 | 5.8 | 6.2 | 6.6 | 7.0 |  |
|  | Relative error | 1.5 | 1.3 | 2.4 | 4.2 | 4.7 | 4.3 | 3.1 |  | 1.9 | 3.6 | 4.9 | 6.1 | 7.0 | 7.9 | 5.2 |
| Yemen | Measurement | 14.9 | 15.8 | 16.6 | 17.5 | 18.4 | 19.3 |  |  | 17.1 | 17.8 | 18.5 | 19.2 | 20.0 | 20.7 |  |
|  | Projection | 15.1 | 16.3 | 17.6 | 18.9 | 20.4 | 22.0 |  |  | 17.2 | 17.9 | 18.6 | 19.4 | 20.1 | 20.9 |  |
|  | Relative error | 1.3 | 3.2 | 6.0 | 8.0 | 10.9 | 14.0 | 7.2 |  | 0.6 | 0.6 | 0.5 | 1.0 | 0.5 | 1.0 | 0.7 |
| Zambia | Measurement | 6.6 | 7.0 | 7.3 | 7.7 | 8.1 | 8.6 |  |  | 14.1 | 14.6 | 15.2 | 15.7 | 16.3 | 16.8 |  |
|  | Projection | 6.8 | 7.2 | 7.6 | 8.1 | 8.5 | 9.0 |  |  | 14.1 | 14.6 | 15.0 | 15.5 | 16.0 | 16.5 |  |
|  | Relative error | 3.0 | 2.9 | 4.1 | 5.2 | 4.9 | 4.7 | 4.1 |  | 0.0 | 0.0 | 1.3 | 1.3 | 1.8 | 1.8 | 1.0 |
| Zimbabwe | Measurement | 4.7 | 5.0 | 5.4 | 5.7 | 6.2 | 6.6 |  |  | 17.7 | 18.6 | 19.5 | 20.4 | 21.4 | 22.4 |  |
|  | Projection | 5.0 | 5.2 | 5.6 | 6.4 | 6.7 | 7.2 |  |  | 17.7 | 18.7 | 19.7 | 20.7 | 21.8 | 23.0 |  |
|  | Relative error | 6.4 | 4.0 | 3.7 | 12.3 | 8.1 | 9.1 | 7.3 |  | 0.0 | 0.5 | 1.0 | 1.5 | 1.9 | 2.7 | 1.3 |

Relative error=100%*(|Measurement-Projection|/Projection)

**Table S6. Observed and projected prevalence (95% CI) of overweight and obesity among children by regions and countries’ income level from 1975 to 2030**

|  | Year | Region ^a^ | | | | | | |  | Countries’ income level ^b^ | | | |
| --- | --- | --- | --- | --- | --- | --- | --- | --- | --- | --- | --- | --- | --- |
|  |  | Global | Africa | America | Southeast Asia | Europe | Mediterranean | Western Pacific |  | Low-income | Lower-middle-income | Upper-middle-income | High-income |
| Boys | 1975 | 4.1 | 0.6 | 11.7 | 0.4 | 10.1 | 3.0 | 2.0 |  | 0.7 | 0.9 | 3.2 | 14.4 |
|  | 1976 | 4.2 | 0.6 | 12.1 | 0.5 | 10.5 | 3.2 | 2.1 |  | 0.7 | 0.9 | 3.3 | 14.8 |
|  | 1977 | 4.3 | 0.6 | 12.4 | 0.5 | 10.8 | 3.4 | 2.2 |  | 0.7 | 1.0 | 3.5 | 15.3 |
|  | 1978 | 4.4 | 0.7 | 12.7 | 0.5 | 11.2 | 3.6 | 2.3 |  | 0.8 | 1.1 | 3.6 | 15.7 |
|  | 1979 | 4.6 | 0.7 | 13.1 | 0.6 | 11.6 | 3.8 | 2.5 |  | 0.8 | 1.1 | 3.8 | 16.1 |
|  | 1980 | 4.7 | 0.7 | 13.5 | 0.6 | 12.0 | 4.0 | 2.6 |  | 0.9 | 1.2 | 4.0 | 16.6 |
|  | 1981 | 4.9 | 0.8 | 13.9 | 0.7 | 12.3 | 4.3 | 2.8 |  | 0.9 | 1.3 | 4.3 | 17.1 |
|  | 1982 | 5.0 | 0.8 | 14.3 | 0.7 | 12.7 | 4.6 | 2.9 |  | 1.0 | 1.3 | 4.6 | 17.5 |
|  | 1983 | 5.2 | 0.9 | 14.8 | 0.8 | 13.1 | 4.9 | 3.1 |  | 1.0 | 1.4 | 4.9 | 18.0 |
|  | 1984 | 5.4 | 1.0 | 15.3 | 0.8 | 13.5 | 5.2 | 3.3 |  | 1.1 | 1.5 | 5.2 | 18.5 |
|  | 1985 | 5.6 | 1.1 | 15.8 | 0.9 | 13.8 | 5.5 | 3.6 |  | 1.1 | 1.6 | 5.6 | 19.0 |
|  | 1986 | 5.8 | 1.1 | 16.3 | 1.0 | 14.2 | 5.9 | 3.8 |  | 1.2 | 1.7 | 6.0 | 19.6 |
|  | 1987 | 6.1 | 1.2 | 16.9 | 1.0 | 14.6 | 6.3 | 4.1 |  | 1.2 | 1.9 | 6.4 | 20.1 |
|  | 1988 | 6.3 | 1.3 | 17.5 | 1.1 | 14.9 | 6.6 | 4.4 |  | 1.3 | 2.0 | 6.8 | 20.7 |
|  | 1989 | 6.6 | 1.4 | 18.0 | 1.2 | 15.3 | 7.0 | 4.7 |  | 1.4 | 2.1 | 7.3 | 21.2 |
|  | 1990 | 6.9 | 1.5 | 18.6 | 1.3 | 15.7 | 7.4 | 5.1 |  | 1.5 | 2.3 | 7.8 | 21.8 |
|  | 1991 | 7.2 | 1.7 | 19.3 | 1.4 | 16.1 | 7.8 | 5.5 |  | 1.6 | 2.4 | 8.3 | 22.4 |
|  | 1992 | 7.5 | 1.8 | 19.9 | 1.5 | 16.5 | 8.2 | 5.9 |  | 1.7 | 2.6 | 8.8 | 23.1 |
|  | 1993 | 7.8 | 1.9 | 20.5 | 1.7 | 16.9 | 8.6 | 6.4 |  | 1.8 | 2.8 | 9.3 | 23.7 |
|  | 1994 | 8.1 | 2.0 | 21.2 | 1.8 | 17.3 | 8.9 | 6.9 |  | 1.9 | 3.0 | 9.9 | 24.3 |
|  | 1995 | 8.5 | 2.2 | 21.9 | 2.0 | 17.7 | 9.3 | 7.5 |  | 2.1 | 3.1 | 10.5 | 25.0 |
|  | 1996 | 8.8 | 2.3 | 22.5 | 2.1 | 18.0 | 9.7 | 8.0 |  | 2.2 | 3.3 | 11.1 | 25.6 |
|  | 1997 | 9.2 | 2.5 | 23.2 | 2.3 | 18.4 | 10.2 | 8.6 |  | 2.4 | 3.6 | 11.7 | 26.2 |
|  | 1998 | 9.5 | 2.7 | 23.9 | 2.5 | 18.8 | 10.6 | 9.2 |  | 2.5 | 3.8 | 12.3 | 26.8 |
|  | 1999 | 9.9 | 2.8 | 24.6 | 2.7 | 19.2 | 11.0 | 9.9 |  | 2.7 | 4.0 | 13.0 | 27.4 |
|  | 2000 | 10.3 | 3.0 | 25.2 | 2.9 | 19.6 | 11.4 | 10.6 |  | 2.9 | 4.3 | 13.7 | 28.0 |
|  | 2001 | 10.7 | 3.2 | 25.9 | 3.2 | 20.0 | 11.9 | 11.3 |  | 3.1 | 4.5 | 14.4 | 28.6 |
|  | 2002 | 11.2 | 3.4 | 26.5 | 3.4 | 20.5 | 12.4 | 12.1 |  | 3.2 | 4.8 | 15.2 | 29.1 |
|  | 2003 | 11.6 | 3.6 | 27.2 | 3.7 | 20.9 | 12.8 | 12.9 |  | 3.4 | 5.1 | 16.0 | 29.7 |
|  | 2004 | 12.1 | 3.8 | 27.8 | 4.0 | 21.4 | 13.3 | 13.8 |  | 3.7 | 5.4 | 16.9 | 30.2 |
|  | 2005 | 12.5 | 4.0 | 28.4 | 4.4 | 21.9 | 13.8 | 14.7 |  | 3.9 | 5.8 | 17.8 | 30.7 |
|  | 2006 | 13.0 | 4.3 | 29.0 | 4.7 | 22.4 | 14.3 | 15.8 |  | 4.1 | 6.1 | 18.9 | 31.1 |
|  | 2007 | 13.6 | 4.5 | 29.5 | 5.1 | 22.9 | 14.8 | 16.9 |  | 4.4 | 6.5 | 20.0 | 31.6 |
|  | 2008 | 14.1 | 4.8 | 30.1 | 5.5 | 23.5 | 15.3 | 18.2 |  | 4.6 | 6.9 | 21.2 | 32.0 |
|  | 2009 | 14.7 | 5.1 | 30.7 | 5.9 | 24.1 | 15.8 | 19.5 |  | 4.9 | 7.3 | 22.4 | 32.5 |
|  | 2010 | 15.3 | 5.4 | 31.3 | 6.3 | 24.7 | 16.4 | 20.9 |  | 5.2 | 7.8 | 23.7 | 32.9 |
|  | 2011 | 15.9 | 5.7 | 31.8 | 6.8 | 25.3 | 17.0 | 22.4 |  | 5.5 | 8.3 | 25.1 | 33.2 |
|  | 2012 | 16.5 | 6.1 | 32.4 | 7.3 | 25.8 | 17.6 | 23.9 |  | 5.8 | 8.8 | 26.4 | 33.6 |
|  | 2013 | 17.2 | 6.5 | 32.9 | 7.9 | 26.4 | 18.2 | 25.5 |  | 6.1 | 9.3 | 27.8 | 34.0 |
|  | 2014 | 17.8 | 6.9 | 33.5 | 8.4 | 27.0 | 18.8 | 27.1 |  | 6.4 | 9.9 | 29.2 | 34.4 |
|  | 2015 | 18.5 | 7.3 | 34.0 | 9.0 | 27.6 | 19.5 | 28.7 |  | 6.8 | 10.5 | 30.5 | 34.8 |
|  | 2016 | 19.3 | 7.7 | 34.6 | 9.6 | 28.1 | 20.2 | 30.4 |  | 7.2 | 11.1 | 31.9 | 35.1 |
|  | 2017 ^c^ | 20.3 | 8.4 | 35.9 | 11.0 | 28.6 | 20.6 | 30.9 |  | 7.8 | 12.1 | 32.8 | 36.3 |
|  | 2018 ^c^ | 21.1 | 9.0 | 36.6 | 11.7 | 29.1 | 21.2 | 32.5 |  | 8.2 | 12.8 | 34.2 | 36.8 |
|  | 2019 ^c^ | 22.0 | 9.6 | 37.3 | 12.9 | 29.7 | 21.9 | 34.0 |  | 8.8 | 13.7 | 35.6 | 37.3 |
|  | 2020 ^c^ | 22.9 | 10.2 | 38.0 | 13.6 | 30.3 | 22.5 | 35.7 |  | 9.3 | 14.6 | 37.1 | 37.8 |
|  | 2021 ^c^ | 23.8 | 10.9 | 38.7 | 15.0 | 30.9 | 23.2 | 37.3 |  | 9.9 | 15.5 | 38.6 | 38.2 |
|  | 2022 ^c^ | 24.8 | 11.6 | 39.4 | 15.9 | 31.5 | 23.8 | 39.0 |  | 10.4 | 16.5 | 40.1 | 38.7 |
|  | 2023 ^c^ | 25.8 | 12.4 | 40.1 | 17.5 | 32.1 | 24.5 | 40.8 |  | 11.1 | 17.6 | 41.7 | 39.2 |
|  | 2024 ^c^ | 26.8 | 13.2 | 40.8 | 18.5 | 32.7 | 25.2 | 42.6 |  | 11.7 | 18.7 | 43.3 | 39.7 |
|  | 2025 ^c^ | 27.9 | 14.0 | 41.6 | 20.4 | 33.3 | 25.9 | 44.4 |  | 12.4 | 19.9 | 44.9 | 40.1 |
|  | 2026 ^c^ | 29.1 | 15.0 | 42.3 | 21.6 | 34.0 | 26.6 | 46.3 |  | 13.1 | 21.2 | 46.6 | 40.6 |
|  | 2027 ^c^ | 30.3 | 15.9 | 43.0 | 23.8 | 34.6 | 27.3 | 48.2 |  | 14.0 | 22.5 | 48.3 | 41.1 |
|  | 2028 ^c^ | 31.5 | 17.0 | 43.7 | 25.2 | 35.2 | 28.0 | 50.2 |  | 14.7 | 24.1 | 50.0 | 41.5 |
|  | 2029 ^c^ | 32.8 | 18.1 | 44.5 | 27.8 | 35.9 | 28.7 | 52.2 |  | 15.7 | 25.6 | 51.8 | 42.0 |
|  | 2030 ^c^ | 34.2 | 19.3 | 45.2 | 29.4 | 36.5 | 29.4 | 54.2 |  | 16.5 | 27.2 | 53.6 | 42.4 |
|  |  |  |  |  |  |  |  |  |  |  |  |  |  |
| Girls | 1975 | 4.6 | 1.8 | 13.4 | 0.6 | 10.3 | 4.8 | 2.0 |  | 1.5 | 1.4 | 4.0 | 14.3 |
|  | 1976 | 4.8 | 1.9 | 13.7 | 0.6 | 10.6 | 5.0 | 2.1 |  | 1.6 | 1.5 | 4.2 | 14.6 |
|  | 1977 | 4.9 | 2.0 | 14.1 | 0.6 | 10.9 | 5.3 | 2.2 |  | 1.7 | 1.6 | 4.3 | 15.0 |
|  | 1978 | 5.0 | 2.2 | 14.5 | 0.7 | 11.2 | 5.5 | 2.3 |  | 1.8 | 1.7 | 4.5 | 15.3 |
|  | 1979 | 5.1 | 2.3 | 14.9 | 0.7 | 11.5 | 5.8 | 2.4 |  | 1.9 | 1.7 | 4.7 | 15.7 |
|  | 1980 | 5.3 | 2.4 | 15.3 | 0.8 | 11.8 | 6.1 | 2.5 |  | 2.0 | 1.8 | 4.9 | 16.0 |
|  | 1981 | 5.4 | 2.6 | 15.7 | 0.8 | 12.1 | 6.4 | 2.6 |  | 2.2 | 1.9 | 5.1 | 16.4 |
|  | 1982 | 5.6 | 2.7 | 16.2 | 0.9 | 12.4 | 6.8 | 2.7 |  | 2.3 | 2.0 | 5.4 | 16.7 |
|  | 1983 | 5.8 | 2.9 | 16.6 | 1.0 | 12.6 | 7.1 | 2.9 |  | 2.5 | 2.2 | 5.7 | 17.1 |
|  | 1984 | 6.0 | 3.1 | 17.1 | 1.0 | 12.9 | 7.5 | 3.0 |  | 2.6 | 2.3 | 6.0 | 17.4 |
|  | 1985 | 6.2 | 3.3 | 17.6 | 1.1 | 13.2 | 7.8 | 3.2 |  | 2.8 | 2.4 | 6.4 | 17.8 |
|  | 1986 | 6.4 | 3.5 | 18.1 | 1.2 | 13.5 | 8.2 | 3.4 |  | 3.0 | 2.5 | 6.7 | 18.2 |
|  | 1987 | 6.6 | 3.7 | 18.6 | 1.3 | 13.8 | 8.6 | 3.6 |  | 3.2 | 2.7 | 7.1 | 18.6 |
|  | 1988 | 6.9 | 3.9 | 19.1 | 1.4 | 14.0 | 9.0 | 3.8 |  | 3.4 | 2.9 | 7.5 | 19.0 |
|  | 1989 | 7.1 | 4.2 | 19.7 | 1.5 | 14.3 | 9.4 | 4.0 |  | 3.6 | 3.0 | 7.9 | 19.5 |
|  | 1990 | 7.4 | 4.4 | 20.2 | 1.5 | 14.6 | 9.7 | 4.3 |  | 3.8 | 3.2 | 8.3 | 20.0 |
|  | 1991 | 7.6 | 4.7 | 20.7 | 1.7 | 14.9 | 10.1 | 4.5 |  | 4.0 | 3.4 | 8.7 | 20.4 |
|  | 1992 | 7.9 | 5.0 | 21.3 | 1.8 | 15.2 | 10.5 | 4.8 |  | 4.3 | 3.6 | 9.1 | 21.0 |
|  | 1993 | 8.2 | 5.3 | 21.8 | 1.9 | 15.5 | 10.8 | 5.1 |  | 4.5 | 3.8 | 9.5 | 21.5 |
|  | 1994 | 8.5 | 5.6 | 22.4 | 2.0 | 15.8 | 11.2 | 5.4 |  | 4.8 | 4.0 | 9.9 | 22.0 |
|  | 1995 | 8.8 | 5.9 | 23.0 | 2.2 | 16.2 | 11.6 | 5.8 |  | 5.1 | 4.2 | 10.3 | 22.6 |
|  | 1996 | 9.1 | 6.2 | 23.5 | 2.3 | 16.5 | 11.9 | 6.1 |  | 5.4 | 4.4 | 10.8 | 23.1 |
|  | 1997 | 9.4 | 6.5 | 24.1 | 2.5 | 16.8 | 12.3 | 6.5 |  | 5.7 | 4.6 | 11.2 | 23.6 |
|  | 1998 | 9.7 | 6.9 | 24.7 | 2.6 | 17.1 | 12.7 | 6.8 |  | 6.0 | 4.8 | 11.6 | 24.1 |
|  | 1999 | 10.0 | 7.2 | 25.2 | 2.8 | 17.4 | 13.1 | 7.2 |  | 6.3 | 5.1 | 12.0 | 24.6 |
|  | 2000 | 10.3 | 7.6 | 25.7 | 3.0 | 17.7 | 13.5 | 7.6 |  | 6.7 | 5.3 | 12.5 | 25.1 |
|  | 2001 | 10.7 | 8.0 | 26.2 | 3.2 | 18.1 | 13.8 | 8.0 |  | 7.0 | 5.6 | 13.0 | 25.6 |
|  | 2002 | 11.0 | 8.3 | 26.7 | 3.4 | 18.5 | 14.2 | 8.4 |  | 7.4 | 5.9 | 13.5 | 26.0 |
|  | 2003 | 11.4 | 8.7 | 27.2 | 3.6 | 18.8 | 14.6 | 8.9 |  | 7.8 | 6.1 | 14.0 | 26.4 |
|  | 2004 | 11.7 | 9.2 | 27.6 | 3.9 | 19.2 | 15.0 | 9.4 |  | 8.2 | 6.4 | 14.6 | 26.8 |
|  | 2005 | 12.1 | 9.6 | 28.1 | 4.1 | 19.6 | 15.4 | 9.9 |  | 8.6 | 6.8 | 15.3 | 27.2 |
|  | 2006 | 12.5 | 10.0 | 28.5 | 4.4 | 20.0 | 15.8 | 10.5 |  | 9.0 | 7.1 | 16.0 | 27.6 |
|  | 2007 | 13.0 | 10.5 | 28.9 | 4.7 | 20.5 | 16.2 | 11.2 |  | 9.5 | 7.4 | 16.7 | 27.9 |
|  | 2008 | 13.4 | 10.9 | 29.3 | 5.0 | 20.9 | 16.6 | 11.9 |  | 9.9 | 7.8 | 17.5 | 28.2 |
|  | 2009 | 13.9 | 11.4 | 29.7 | 5.3 | 21.3 | 17.1 | 12.6 |  | 10.4 | 8.1 | 18.4 | 28.5 |
|  | 2010 | 14.3 | 11.9 | 30.1 | 5.7 | 21.8 | 17.5 | 13.4 |  | 10.9 | 8.5 | 19.2 | 28.8 |
|  | 2011 | 14.8 | 12.4 | 30.5 | 6.0 | 22.2 | 18.0 | 14.2 |  | 11.4 | 8.9 | 20.1 | 29.1 |
|  | 2012 | 15.3 | 12.9 | 30.9 | 6.4 | 22.6 | 18.5 | 15.1 |  | 11.9 | 9.4 | 21.0 | 29.4 |
|  | 2013 | 15.8 | 13.4 | 31.3 | 6.8 | 23.0 | 19.0 | 16.0 |  | 12.4 | 9.8 | 21.8 | 29.7 |
|  | 2014 | 16.4 | 13.9 | 31.7 | 7.2 | 23.4 | 19.6 | 16.9 |  | 13.0 | 10.3 | 22.7 | 29.9 |
|  | 2015 | 16.9 | 14.5 | 32.1 | 7.7 | 23.8 | 20.1 | 17.9 |  | 13.5 | 10.8 | 23.6 | 30.2 |
|  | 2016 | 17.5 | 15.1 | 32.6 | 8.1 | 24.2 | 20.7 | 18.8 |  | 14.1 | 11.3 | 24.4 | 30.5 |
|  | 2017 ^c^ | 18.0 | 16.1 | 33.3 | 9.1 | 25.3 | 20.4 | 19.8 |  | 14.5 | 12.2 | 24.9 | 31.5 |
|  | 2018 ^c^ | 18.5 | 16.8 | 33.8 | 9.7 | 25.8 | 20.8 | 20.9 |  | 15.1 | 12.8 | 25.8 | 31.9 |
|  | 2019 ^c^ | 19.2 | 17.5 | 34.2 | 10.3 | 26.4 | 21.2 | 22.0 |  | 15.7 | 13.5 | 26.7 | 32.2 |
|  | 2020 ^c^ | 19.8 | 18.3 | 34.7 | 11.1 | 26.9 | 21.7 | 23.3 |  | 16.3 | 14.2 | 27.6 | 32.6 |
|  | 2021 ^c^ | 20.5 | 19.1 | 35.1 | 11.8 | 27.5 | 22.1 | 24.6 |  | 16.9 | 15.0 | 28.5 | 33.0 |
|  | 2022 ^c^ | 21.1 | 20.0 | 35.6 | 12.7 | 28.1 | 22.5 | 26.0 |  | 17.5 | 15.8 | 29.5 | 33.4 |
|  | 2023 ^c^ | 21.8 | 20.8 | 36.0 | 13.5 | 28.7 | 22.9 | 27.4 |  | 18.1 | 16.6 | 30.4 | 33.7 |
|  | 2024 ^c^ | 22.5 | 21.7 | 36.5 | 14.4 | 29.3 | 23.3 | 28.9 |  | 18.8 | 17.5 | 31.4 | 34.1 |
|  | 2025 ^c^ | 23.3 | 22.7 | 36.9 | 15.3 | 30.0 | 23.7 | 30.6 |  | 19.4 | 18.4 | 32.4 | 34.5 |
|  | 2026 ^c^ | 24.0 | 23.7 | 37.3 | 16.4 | 30.6 | 24.2 | 32.3 |  | 20.1 | 19.4 | 33.5 | 34.8 |
|  | 2027 ^c^ | 24.8 | 24.7 | 37.8 | 17.5 | 31.3 | 24.6 | 34.1 |  | 20.8 | 20.4 | 34.5 | 35.2 |
|  | 2028 ^c^ | 25.6 | 25.8 | 38.2 | 18.8 | 31.9 | 25.0 | 36.0 |  | 21.5 | 21.5 | 35.6 | 35.5 |
|  | 2029 ^c^ | 26.5 | 27.0 | 38.6 | 19.9 | 32.6 | 25.4 | 38.0 |  | 22.2 | 22.6 | 36.6 | 35.9 |
|  | 2030 ^c^ | 27.4 | 28.1 | 39.1 | 21.4 | 33.3 | 25.8 | 40.1 |  | 22.9 | 23.8 | 37.7 | 36.3 |

^a^ Region was evaluated according to the standards of the United Nations. ^b^ Countries’ income was evaluated according to the standards of the World Bank. ^c^ Projected data. The confidence intervals for the prevalence, are available from the authors.

**Table S7. Observed and projected prevalence (95% CI) of overweight and obesity among children by countries from 1975 to 2030**

|  | Year | Afghanistan | Albania | Algeria | Andorra | Angola | Antigua and Barbuda | Argentina | Armenia | Australia | Austria | Azerbaijan | Bahamas | Bahrain | Bangladesh | Barbados |
| --- | --- | --- | --- | --- | --- | --- | --- | --- | --- | --- | --- | --- | --- | --- | --- | --- |
| **Boys** | 1975 | 0.3 | 3.5 | 3.5 | 24.8 | 0.2 | 3.8 | 16.5 | 6.4 | 19.6 | 12.0 | 4.8 | 11.4 | 15.1 | 0.3 | 4.3 |
|  | 1980 | 0.5 | 4.5 | 4.6 | 29.5 | 0.3 | 4.7 | 18.9 | 7.7 | 21.1 | 14.0 | 5.6 | 13.6 | 16.9 | 0.4 | 5.4 |
|  | 1985 | 0.8 | 5.9 | 6.6 | 33.2 | 0.5 | 6.2 | 21.6 | 9.2 | 22.9 | 16.3 | 6.8 | 16.6 | 19.3 | 0.7 | 6.6 |
|  | 1990 | 1.2 | 7.7 | 9.4 | 35.3 | 0.7 | 8.2 | 24.3 | 10.7 | 24.9 | 19.2 | 8.2 | 20.0 | 21.6 | 1.2 | 7.8 |
|  | 1995 | 1.9 | 10.2 | 12.8 | 36.3 | 1.2 | 10.7 | 27.0 | 12.0 | 27.6 | 22.1 | 9.7 | 23.5 | 24.1 | 1.9 | 10.0 |
|  | 2000 | 2.9 | 13.5 | 16.7 | 37.4 | 1.9 | 13.4 | 30.1 | 12.9 | 30.2 | 24.2 | 11.1 | 26.5 | 27.0 | 2.9 | 13.1 |
|  | 2005 | 4.2 | 17.6 | 20.9 | 38.1 | 2.9 | 16.5 | 33.3 | 14.0 | 32.4 | 26.1 | 12.7 | 29.3 | 29.6 | 4.3 | 16.8 |
|  | 2010 | 6.1 | 22.6 | 25.5 | 38.4 | 4.5 | 20.3 | 36.7 | 15.7 | 33.9 | 27.5 | 14.9 | 32.2 | 32.9 | 6.2 | 20.9 |
|  | 2015 | 8.5 | 28.0 | 30.6 | 38.7 | 6.6 | 24.9 | 40.2 | 18.1 | 35.3 | 29.1 | 17.9 | 35.5 | 36.0 | 8.7 | 26.0 |
|  | 2020 ^a^ | 14.6 | 38.5 | 36.5 | 37.0 | 8.2 | 29.5 | 43.9 | 19.1 | 37.8 | 30.5 | 20.1 | 38.6 | 39.4 | 14.7 | 31.5 |
|  | 2025 ^a^ | 23.2 | 50.3 | 42.8 | 35.3 | 10.4 | 34.7 | 47.8 | 20.7 | 39.7 | 31.7 | 22.8 | 41.7 | 43.0 | 22.7 | 37.7 |
|  | 2030 ^a^ | 35.2 | 65.6 | 49.6 | 33.1 | 13.0 | 40.4 | 51.8 | 22.4 | 41.6 | 32.6 | 25.8 | 44.8 | 46.7 | 34.9 | 44.6 |
|  |  |  |  |  |  |  |  |  |  |  |  |  |  |  |  |  |
| **Girls** | 1975 | 0.5 | 3.0 | 6.1 | 22.4 | 1.3 | 6.0 | 17.9 | 10.0 | 20.3 | 11.8 | 7.4 | 15.3 | 19.7 | 0.4 | 6.6 |
|  | 1980 | 0.7 | 3.8 | 7.7 | 25.9 | 1.8 | 7.3 | 19.7 | 11.6 | 21.9 | 13.0 | 8.5 | 17.7 | 21.3 | 0.6 | 8.0 |
|  | 1985 | 1.1 | 4.7 | 10.1 | 28.5 | 2.5 | 9.0 | 21.7 | 13.2 | 23.6 | 14.5 | 9.8 | 20.7 | 23.2 | 1.0 | 9.5 |
|  | 1990 | 1.6 | 6.0 | 13.1 | 29.8 | 3.5 | 11.3 | 23.5 | 14.6 | 25.3 | 16.3 | 11.2 | 23.8 | 25.1 | 1.5 | 10.8 |
|  | 1995 | 2.3 | 7.7 | 16.4 | 30.4 | 4.9 | 13.8 | 25.1 | 15.6 | 27.6 | 18.0 | 12.5 | 26.6 | 27.0 | 2.1 | 13.0 |
|  | 2000 | 3.3 | 9.9 | 19.8 | 31.1 | 6.7 | 16.4 | 26.7 | 16.4 | 29.6 | 19.4 | 13.7 | 28.9 | 28.8 | 3.1 | 16.0 |
|  | 2005 | 4.7 | 12.7 | 23.1 | 31.7 | 8.8 | 19.3 | 28.2 | 17.2 | 31.1 | 20.7 | 15.0 | 30.8 | 30.5 | 4.3 | 19.3 |
|  | 2010 | 6.6 | 16.0 | 26.2 | 32.1 | 11.4 | 22.6 | 29.7 | 18.3 | 31.8 | 21.8 | 16.5 | 32.7 | 31.9 | 6.0 | 22.9 |
|  | 2015 | 9.2 | 19.7 | 29.6 | 32.6 | 14.3 | 26.6 | 31.5 | 19.7 | 32.4 | 23.2 | 18.5 | 34.9 | 33.5 | 8.2 | 27.2 |
|  | 2020 ^a^ | 14.3 | 25.9 | 33.8 | 31.5 | 20.5 | 30.3 | 32.6 | 19.9 | 33.9 | 24.3 | 19.1 | 36.2 | 35.1 | 13.3 | 31.8 |
|  | 2025 ^a^ | 20.2 | 32.9 | 37.9 | 30.5 | 27.6 | 34.5 | 33.9 | 20.5 | 34.6 | 25.4 | 20.4 | 37.6 | 36.7 | 19.6 | 36.9 |
|  | 2030 ^a^ | 29.6 | 41.7 | 42.1 | 29.3 | 37.4 | 39.1 | 35.2 | 21.0 | 35.2 | 26.4 | 21.7 | 38.8 | 38.3 | 29.0 | 42.6 |
|  |  |  |  |  |  |  |  |  |  |  |  |  |  |  |  |  |
|  |  | Belarus | Belgium | Belize | Benin | Bhutan | Bolivia (Plurinational State of) | Bosnia and Herzegovina | Botswana | Brazil | Brunei Darussalam | Bulgaria | Burkina Faso | Burundi | Cabo Verde | Cambodia |
| **Boys** | 1975 | 4.7 | 18.3 | 6.6 | 0.3 | 0.2 | 4.2 | 3.1 | 0.2 | 5.9 | 5.2 | 7.2 | 0.1 | 0.2 | 0.3 | 0.4 |
|  | 1980 | 6.5 | 20.1 | 7.5 | 0.5 | 0.4 | 5.4 | 4.3 | 0.3 | 7.7 | 5.9 | 9.6 | 0.2 | 0.3 | 0.5 | 0.5 |
|  | 1985 | 9.0 | 22.1 | 8.9 | 0.8 | 0.7 | 7.5 | 5.9 | 0.5 | 10.0 | 7.2 | 12.2 | 0.3 | 0.5 | 0.8 | 0.8 |
|  | 1990 | 11.7 | 23.9 | 10.8 | 1.3 | 1.1 | 10.2 | 8.0 | 1.1 | 12.7 | 9.5 | 14.7 | 0.5 | 0.8 | 1.5 | 1.5 |
|  | 1995 | 14.2 | 25.2 | 13.3 | 1.9 | 1.9 | 13.3 | 10.3 | 2.1 | 15.5 | 12.6 | 17.4 | 0.8 | 1.3 | 2.5 | 2.5 |
|  | 2000 | 16.3 | 25.7 | 16.1 | 2.7 | 3.1 | 16.3 | 12.8 | 3.4 | 18.5 | 16.4 | 20.4 | 1.3 | 1.9 | 3.6 | 3.8 |
|  | 2005 | 18.6 | 25.4 | 19.5 | 3.7 | 4.8 | 19.2 | 15.9 | 5.0 | 21.8 | 20.6 | 23.6 | 2.0 | 2.8 | 4.9 | 5.8 |
|  | 2010 | 21.8 | 24.5 | 23.2 | 5.0 | 7.2 | 22.3 | 19.9 | 7.1 | 25.5 | 25.5 | 27.9 | 3.2 | 4.1 | 6.4 | 8.5 |
|  | 2015 | 25.7 | 23.8 | 27.5 | 6.7 | 10.1 | 25.6 | 24.3 | 9.8 | 29.0 | 30.7 | 32.7 | 4.8 | 5.9 | 8.1 | 12.7 |
|  | 2020 ^a^ | 28.3 | 21.8 | 32.3 | 9.0 | 20.1 | 29.6 | 28.7 | 19.7 | 33.1 | 37.2 | 36.8 | 8.5 | 10.4 | 11.8 | 20.4 |
|  | 2025 ^a^ | 31.5 | 19.6 | 37.6 | 12.0 | 31.6 | 33.4 | 33.7 | 32.8 | 37.4 | 44.1 | 41.7 | 13.7 | 16.3 | 16.7 | 31.5 |
|  | 2030 ^a^ | 34.9 | 16.8 | 43.3 | 16.1 | 52.8 | 37.5 | 39.2 | 54.6 | 41.9 | 51.7 | 47.0 | 22.1 | 25.4 | 23.5 | 48.7 |
|  |  |  |  |  |  |  |  |  |  |  |  |  |  |  |  |  |
| **Girls** | 1975 | 4.4 | 21.1 | 9.4 | 1.6 | 0.4 | 7.6 | 2.5 | 1.1 | 8.6 | 4.7 | 5.8 | 0.7 | 1.0 | 1.6 | 0.5 |
|  | 1980 | 6.0 | 22.2 | 10.5 | 2.4 | 0.6 | 9.5 | 3.4 | 1.6 | 10.6 | 5.3 | 7.5 | 1.0 | 1.5 | 2.2 | 0.7 |
|  | 1985 | 7.8 | 23.5 | 12.0 | 3.5 | 0.9 | 12.1 | 4.6 | 2.7 | 12.9 | 6.2 | 9.2 | 1.4 | 2.1 | 3.2 | 1.0 |
|  | 1990 | 9.8 | 24.6 | 13.9 | 4.9 | 1.4 | 15.3 | 6.0 | 4.9 | 15.3 | 7.8 | 10.9 | 2.0 | 3.1 | 5.2 | 1.6 |
|  | 1995 | 11.5 | 25.4 | 16.2 | 6.5 | 2.1 | 18.6 | 7.5 | 7.9 | 17.5 | 9.8 | 12.6 | 2.8 | 4.4 | 7.5 | 2.4 |
|  | 2000 | 12.9 | 25.7 | 18.9 | 8.3 | 3.2 | 21.6 | 9.1 | 11.3 | 19.6 | 12.2 | 14.6 | 3.8 | 6.1 | 9.6 | 3.3 |
|  | 2005 | 14.4 | 25.4 | 21.7 | 10.3 | 4.7 | 24.3 | 11.2 | 15.0 | 21.7 | 14.8 | 16.7 | 5.3 | 8.1 | 11.7 | 4.5 |
|  | 2010 | 16.2 | 24.8 | 24.6 | 12.5 | 6.6 | 26.8 | 13.9 | 19.2 | 23.8 | 17.8 | 19.4 | 7.2 | 10.6 | 13.8 | 6.1 |
|  | 2015 | 18.3 | 24.4 | 28.0 | 15.0 | 8.9 | 29.4 | 16.8 | 23.7 | 25.9 | 21.0 | 22.5 | 9.6 | 13.6 | 15.8 | 8.2 |
|  | 2020 ^a^ | 19.7 | 23.2 | 31.9 | 17.7 | 14.8 | 32.3 | 19.6 | 29.5 | 28.1 | 25.3 | 25.1 | 14.0 | 20.9 | 18.7 | 12.5 |
|  | 2025 ^a^ | 21.4 | 21.8 | 35.9 | 20.6 | 22.2 | 35.0 | 22.8 | 35.5 | 30.3 | 30.3 | 28.2 | 19.5 | 29.7 | 21.4 | 18.0 |
|  | 2030 ^a^ | 23.1 | 20.0 | 40.3 | 23.8 | 33.3 | 37.7 | 26.4 | 42.1 | 32.5 | 36.3 | 31.5 | 27.2 | 42.2 | 24.2 | 25.7 |
|  |  |  |  |  |  |  |  |  |  |  |  |  |  |  |  |  |
|  |  | Cameroon | Canada | Central African Republic | Chad | Chile | China | Colombia | Comoros | Congo | Cook Islands | Costa Rica | Cote d'Ivoire | Croatia | Cuba | Cyprus |
| **Boys** | 1975 | 0.4 | 15.2 | 0.4 | 0.2 | 13.9 | 1.2 | 5.9 | 0.5 | 0.6 | 15.6 | 4.4 | 0.6 | 5.1 | 8.6 | 9.4 |
|  | 1980 | 0.7 | 16.9 | 0.6 | 0.4 | 16.1 | 1.6 | 7.4 | 0.7 | 0.8 | 18.7 | 5.6 | 0.9 | 7.2 | 10.5 | 13.9 |
|  | 1985 | 1.1 | 19.5 | 0.8 | 0.6 | 18.4 | 2.5 | 9.4 | 1.0 | 1.1 | 22.5 | 7.4 | 1.3 | 9.7 | 12.9 | 19.7 |
|  | 1990 | 1.6 | 22.4 | 1.2 | 0.8 | 20.8 | 4.0 | 11.6 | 1.6 | 1.5 | 27.8 | 10.2 | 1.8 | 12.4 | 15.5 | 24.6 |
|  | 1995 | 2.4 | 25.4 | 1.6 | 1.2 | 23.6 | 6.8 | 13.7 | 2.3 | 2.0 | 33.5 | 13.5 | 2.5 | 15.3 | 18.9 | 28.1 |
|  | 2000 | 3.3 | 28.4 | 2.2 | 1.8 | 26.5 | 10.6 | 15.7 | 3.1 | 2.7 | 40.3 | 17.0 | 3.5 | 18.5 | 21.7 | 30.6 |
|  | 2005 | 4.5 | 30.8 | 3.1 | 2.6 | 29.5 | 15.4 | 17.6 | 4.1 | 3.8 | 47.6 | 21.0 | 4.8 | 22.5 | 24.1 | 32.8 |
|  | 2010 | 5.9 | 32.3 | 4.4 | 3.6 | 32.8 | 23.1 | 19.7 | 5.4 | 5.4 | 53.9 | 25.3 | 6.5 | 26.9 | 27.2 | 34.1 |
|  | 2015 | 7.6 | 33.7 | 6.2 | 5.1 | 36.5 | 33.1 | 22.0 | 7.1 | 7.5 | 59.2 | 29.3 | 8.4 | 31.7 | 30.3 | 35.9 |
|  | 2020 ^a^ | 9.3 | 36.0 | 7.6 | 6.3 | 40.1 | 41.6 | 23.9 | 10.8 | 10.2 | 68.4 | 35.0 | 12.9 | 36.7 | 33.5 | 35.4 |
|  | 2025 ^a^ | 11.3 | 37.6 | 9.5 | 7.9 | 44.0 | 52.6 | 26.1 | 15.3 | 14.0 | 76.6 | 40.5 | 18.2 | 42.2 | 36.7 | 34.7 |
|  | 2030 ^a^ | 13.5 | 39.0 | 11.6 | 9.7 | 48.1 | 65.0 | 28.2 | 21.5 | 19.2 | 85.3 | 46.5 | 25.6 | 48.0 | 40.1 | 33.3 |
|  |  |  |  |  |  |  |  |  |  |  |  |  |  |  |  |  |
| **Girls** | 1975 | 2.2 | 15.8 | 2.0 | 1.2 | 17.6 | 1.3 | 8.5 | 1.9 | 2.4 | 25.6 | 6.9 | 2.5 | 4.2 | 11.6 | 8.8 |
|  | 1980 | 3.3 | 17.4 | 2.8 | 1.8 | 19.6 | 1.7 | 10.5 | 2.7 | 3.2 | 29.2 | 8.6 | 3.5 | 5.6 | 13.8 | 12.3 |
|  | 1985 | 4.7 | 19.5 | 3.6 | 2.5 | 21.6 | 2.3 | 12.6 | 3.7 | 4.3 | 33.4 | 10.8 | 4.7 | 7.3 | 16.2 | 16.5 |
|  | 1990 | 6.5 | 21.9 | 4.7 | 3.4 | 23.3 | 3.4 | 15.0 | 5.2 | 5.4 | 38.7 | 13.9 | 6.1 | 9.0 | 18.6 | 19.9 |
|  | 1995 | 8.5 | 24.2 | 5.9 | 4.5 | 25.2 | 5.2 | 17.2 | 6.9 | 6.6 | 44.4 | 17.5 | 7.7 | 10.8 | 21.3 | 22.4 |
|  | 2000 | 10.7 | 26.4 | 7.4 | 5.8 | 27.0 | 7.2 | 19.3 | 8.7 | 8.0 | 50.3 | 21.3 | 9.6 | 12.9 | 23.3 | 24.4 |
|  | 2005 | 12.9 | 28.0 | 9.2 | 7.4 | 28.9 | 9.8 | 21.3 | 10.9 | 9.6 | 56.3 | 25.1 | 11.8 | 15.5 | 25.0 | 26.3 |
|  | 2010 | 14.9 | 28.9 | 11.4 | 9.3 | 30.8 | 14.0 | 23.4 | 13.4 | 11.4 | 61.3 | 28.9 | 14.1 | 18.4 | 26.8 | 27.7 |
|  | 2015 | 17.0 | 29.8 | 14.0 | 11.4 | 33.0 | 19.7 | 25.7 | 16.4 | 13.3 | 65.2 | 32.3 | 16.4 | 21.7 | 28.6 | 29.4 |
|  | 2020 ^a^ | 19.8 | 32.6 | 17.4 | 14.5 | 34.6 | 24.1 | 27.6 | 19.4 | 15.4 | 72.2 | 37.4 | 19.3 | 24.8 | 30.0 | 29.6 |
|  | 2025 ^a^ | 22.4 | 34.0 | 21.5 | 18.2 | 36.4 | 30.0 | 29.7 | 22.8 | 17.7 | 78.1 | 42.1 | 22.2 | 28.4 | 31.4 | 29.7 |
|  | 2030 ^a^ | 25.1 | 35.2 | 26.5 | 22.7 | 38.3 | 36.6 | 31.7 | 26.5 | 20.1 | 84.0 | 47.1 | 25.4 | 32.2 | 32.6 | 29.4 |
|  |  | Czechia | Democratic People's Republic of Korea | Democratic Republic of the Congo | Denmark | Djibouti | Dominica | Dominican Republic | Ecuador | Egypt | El Salvador | Equatorial Guinea | Eritrea | Estonia | Eswatini | Ethiopia |
| **Boys** | 1975 | 10.1 | 4.7 | 0.3 | 13.8 | 3.2 | 4.3 | 4.5 | 4.5 | 5.9 | 4.5 | 0.4 | 0.3 | 8.3 | 0.2 | 0.2 |
|  | 1980 | 13.0 | 6.2 | 0.4 | 16.8 | 4.3 | 5.8 | 6.5 | 6.1 | 7.7 | 5.8 | 0.5 | 0.4 | 9.8 | 0.3 | 0.3 |
|  | 1985 | 15.5 | 7.3 | 0.6 | 19.7 | 5.6 | 8.5 | 9.0 | 8.2 | 10.0 | 7.7 | 0.6 | 0.6 | 11.4 | 0.5 | 0.5 |
|  | 1990 | 17.1 | 9.2 | 0.8 | 22.2 | 6.9 | 12.7 | 11.6 | 10.9 | 13.0 | 10.1 | 1.0 | 1.0 | 13.0 | 0.9 | 0.8 |
|  | 1995 | 18.6 | 12.1 | 1.2 | 24.3 | 8.0 | 16.9 | 14.6 | 13.8 | 16.5 | 13.1 | 1.6 | 1.5 | 14.4 | 1.3 | 1.2 |
|  | 2000 | 21.2 | 14.8 | 1.8 | 26.0 | 9.2 | 20.1 | 18.2 | 16.9 | 20.6 | 16.9 | 2.4 | 2.1 | 15.5 | 2.0 | 1.7 |
|  | 2005 | 23.7 | 18.3 | 2.7 | 26.7 | 10.1 | 23.3 | 22.6 | 20.0 | 25.1 | 20.4 | 3.2 | 3.0 | 16.7 | 3.1 | 2.4 |
|  | 2010 | 26.9 | 22.4 | 4.0 | 26.8 | 11.2 | 27.1 | 27.4 | 23.2 | 30.1 | 23.6 | 4.5 | 4.3 | 19.2 | 4.9 | 3.3 |
|  | 2015 | 31.6 | 27.2 | 5.8 | 26.9 | 12.7 | 31.2 | 32.2 | 26.7 | 35.0 | 26.9 | 6.3 | 6.0 | 22.3 | 7.6 | 4.4 |
|  | 2020 ^a^ | 34.1 | 32.2 | 8.5 | 26.2 | 13.5 | 35.3 | 37.8 | 30.6 | 39.1 | 31.8 | 9.0 | 9.4 | 23.7 | 12.4 | 7.4 |
|  | 2025 ^a^ | 37.9 | 37.8 | 12.5 | 25.1 | 14.6 | 39.5 | 43.8 | 34.6 | 43.8 | 36.4 | 12.7 | 14.0 | 26.1 | 19.5 | 11.2 |
|  | 2030 ^a^ | 42.0 | 44.0 | 18.3 | 23.4 | 15.6 | 43.8 | 50.3 | 38.9 | 48.5 | 41.2 | 17.9 | 20.9 | 28.6 | 31.2 | 16.5 |
|  |  |  |  |  |  |  |  |  |  |  |  |  |  |  |  |  |
| **Girls** | 1975 | 8.1 | 3.8 | 1.6 | 15.6 | 7.8 | 7.0 | 7.2 | 7.4 | 9.4 | 7.4 | 1.9 | 1.5 | 8.0 | 1.5 | 1.1 |
|  | 1980 | 10.1 | 4.7 | 2.1 | 17.8 | 9.9 | 9.1 | 9.7 | 9.4 | 11.6 | 9.4 | 2.4 | 2.0 | 9.2 | 2.1 | 1.5 |
|  | 1985 | 11.7 | 5.5 | 2.8 | 19.7 | 12.0 | 12.4 | 12.5 | 12.0 | 14.2 | 11.9 | 3.1 | 2.8 | 10.4 | 3.2 | 2.1 |
|  | 1990 | 12.6 | 6.6 | 3.7 | 21.3 | 13.9 | 17.0 | 15.1 | 14.9 | 17.4 | 14.9 | 4.2 | 3.8 | 11.4 | 4.5 | 3.0 |
|  | 1995 | 13.6 | 8.1 | 5.0 | 22.6 | 15.4 | 21.1 | 18.0 | 17.7 | 20.8 | 18.6 | 5.9 | 5.2 | 12.4 | 6.3 | 4.2 |
|  | 2000 | 15.1 | 9.6 | 6.5 | 23.5 | 17.0 | 24.0 | 21.3 | 20.5 | 24.6 | 22.7 | 7.6 | 7.0 | 13.1 | 8.9 | 5.6 |
|  | 2005 | 16.7 | 11.5 | 8.5 | 23.5 | 18.2 | 26.8 | 24.9 | 23.1 | 28.6 | 26.4 | 9.4 | 9.1 | 14.1 | 12.6 | 7.4 |
|  | 2010 | 18.7 | 13.8 | 10.8 | 23.2 | 19.5 | 29.7 | 28.6 | 25.8 | 32.6 | 29.5 | 11.5 | 11.6 | 15.8 | 17.5 | 9.5 |
|  | 2015 | 21.6 | 16.6 | 13.5 | 23.0 | 21.1 | 32.6 | 32.0 | 28.6 | 36.6 | 32.6 | 14.1 | 14.7 | 17.8 | 23.7 | 11.9 |
|  | 2020 ^a^ | 23.1 | 19.2 | 18.7 | 21.8 | 21.7 | 35.3 | 36.1 | 31.3 | 41.7 | 37.4 | 16.9 | 17.7 | 18.7 | 35.4 | 17.6 |
|  | 2025 ^a^ | 25.5 | 22.4 | 24.5 | 20.4 | 22.5 | 37.8 | 40.2 | 34.0 | 46.6 | 41.6 | 19.9 | 21.2 | 20.3 | 50.3 | 23.9 |
|  | 2030 ^a^ | 27.9 | 25.8 | 32.2 | 18.5 | 23.2 | 40.1 | 44.6 | 36.7 | 51.8 | 45.9 | 23.2 | 25.1 | 22.0 | 71.5 | 32.6 |
|  |  | Fiji | Finland | France | Gabon | Gambia | Georgia | Germany | Ghana | Greece | Grenada | Guatemala | Guinea | Guinea-Bissau | Guyana | Haiti |
| **Boys** | 1975 | 5.7 | 10.3 | 16.7 | 0.6 | 0.4 | 4.7 | 13.4 | 0.5 | 18.1 | 3.3 | 4.1 | 0.3 | 0.3 | 2.9 | 2.1 |
|  | 1980 | 7.1 | 13.4 | 18.6 | 1.2 | 0.6 | 5.8 | 15.2 | 0.6 | 21.3 | 4.1 | 5.2 | 0.4 | 0.4 | 3.7 | 2.7 |
|  | 1985 | 8.9 | 17.0 | 20.4 | 2.0 | 0.9 | 7.3 | 17.1 | 0.9 | 24.4 | 5.4 | 6.7 | 0.6 | 0.6 | 4.8 | 3.9 |
|  | 1990 | 11.3 | 21.0 | 22.3 | 3.1 | 1.4 | 8.9 | 20.0 | 1.3 | 27.5 | 7.2 | 8.9 | 1.0 | 1.0 | 6.3 | 6.0 |
|  | 1995 | 14.4 | 23.8 | 24.8 | 4.4 | 2.2 | 10.5 | 22.5 | 1.9 | 30.1 | 9.7 | 11.6 | 1.4 | 1.6 | 8.4 | 8.9 |
|  | 2000 | 17.8 | 25.9 | 26.5 | 6.0 | 3.2 | 12.1 | 24.1 | 2.7 | 32.8 | 12.6 | 14.6 | 2.0 | 2.5 | 11.1 | 12.4 |
|  | 2005 | 21.3 | 27.3 | 28.1 | 7.6 | 4.5 | 13.7 | 25.3 | 3.6 | 35.8 | 15.9 | 18.1 | 2.8 | 3.6 | 14.5 | 17.2 |
|  | 2010 | 24.7 | 28.1 | 29.6 | 9.3 | 6.1 | 16.2 | 26.6 | 4.8 | 38.5 | 20.0 | 21.7 | 3.9 | 5.0 | 18.5 | 23.2 |
|  | 2015 | 28.6 | 29.2 | 30.9 | 11.3 | 7.9 | 20.1 | 27.8 | 6.1 | 40.8 | 24.8 | 25.7 | 5.3 | 6.8 | 23.1 | 29.3 |
|  | 2020 ^a^ | 33.2 | 29.1 | 32.4 | 13.6 | 12.5 | 22.2 | 28.7 | 9.0 | 43.4 | 32.2 | 30.4 | 8.4 | 8.7 | 31.4 | 36.7 |
|  | 2025 ^a^ | 37.9 | 28.6 | 33.7 | 16.0 | 18.1 | 25.3 | 29.3 | 12.3 | 45.8 | 41.0 | 35.4 | 12.1 | 11.1 | 40.9 | 44.9 |
|  | 2030 ^a^ | 43.0 | 27.5 | 34.8 | 18.5 | 26.4 | 28.7 | 29.7 | 16.8 | 48.1 | 52.3 | 40.8 | 17.6 | 13.5 | 53.3 | 54.0 |
|  |  |  |  |  |  |  |  |  |  |  |  |  |  |  |  |  |
| **Girls** | 1975 | 13.6 | 10.9 | 16.1 | 2.8 | 1.6 | 7.0 | 14.2 | 2.3 | 15.8 | 5.4 | 6.6 | 1.5 | 1.4 | 4.9 | 3.2 |
|  | 1980 | 16.2 | 13.3 | 17.6 | 4.5 | 2.4 | 8.2 | 15.4 | 2.9 | 18.0 | 6.6 | 8.1 | 2.1 | 1.8 | 6.1 | 4.1 |
|  | 1985 | 19.0 | 15.9 | 19.0 | 6.7 | 3.4 | 9.8 | 16.7 | 3.8 | 20.0 | 8.2 | 10.2 | 3.0 | 2.7 | 7.6 | 5.4 |
|  | 1990 | 22.4 | 18.6 | 20.5 | 9.1 | 4.8 | 11.3 | 18.6 | 5.0 | 22.0 | 10.2 | 12.8 | 4.1 | 3.9 | 9.3 | 7.5 |
|  | 1995 | 26.3 | 20.5 | 22.4 | 11.4 | 6.6 | 12.7 | 20.2 | 6.6 | 23.8 | 12.8 | 15.9 | 5.3 | 5.5 | 11.5 | 10.1 |
|  | 2000 | 30.0 | 21.8 | 23.9 | 13.7 | 8.6 | 13.8 | 21.3 | 8.5 | 25.8 | 15.7 | 19.3 | 6.9 | 7.4 | 14.3 | 12.9 |
|  | 2005 | 33.4 | 22.7 | 25.5 | 15.8 | 10.7 | 14.9 | 22.2 | 10.5 | 28.2 | 18.7 | 22.8 | 8.6 | 9.5 | 17.4 | 16.3 |
|  | 2010 | 36.2 | 23.1 | 27.1 | 17.6 | 12.8 | 16.4 | 23.2 | 12.6 | 30.5 | 22.1 | 26.3 | 10.7 | 11.7 | 21.0 | 20.1 |
|  | 2015 | 39.0 | 23.8 | 28.5 | 19.3 | 14.8 | 18.6 | 24.3 | 14.8 | 32.7 | 26.1 | 29.9 | 13.0 | 14.1 | 25.1 | 23.9 |
|  | 2020 ^a^ | 42.8 | 23.3 | 30.3 | 21.4 | 17.7 | 19.5 | 25.1 | 17.6 | 35.1 | 31.4 | 34.5 | 15.4 | 18.4 | 31.8 | 28.7 |
|  | 2025 ^a^ | 46.1 | 22.7 | 31.8 | 23.2 | 20.5 | 20.9 | 25.8 | 20.5 | 37.5 | 37.6 | 39.1 | 18.1 | 23.2 | 39.2 | 33.8 |
|  | 2030 ^a^ | 49.4 | 21.6 | 33.4 | 24.9 | 23.5 | 22.3 | 26.4 | 23.7 | 40.0 | 45.2 | 44.0 | 21.0 | 29.5 | 48.2 | 39.3 |
|  |  | Honduras | Hungary | Iceland | India | Indonesia | Iran (Islamic Republic of) | Iraq | Ireland | Israel | Italy | Jamaica | Japan | Jordan | Kazakhstan | Kenya |
| **Boys** | 1975 | 3.0 | 8.6 | 18.1 | 0.3 | 0.5 | 3.2 | 7.1 | 8.9 | 24.5 | 20.9 | 4.5 | 9.3 | 5.9 | 5.0 | 0.3 |
|  | 1980 | 4.2 | 10.5 | 20.5 | 0.4 | 0.7 | 4.6 | 9.6 | 10.9 | 27.3 | 23.5 | 5.9 | 11.6 | 7.3 | 6.1 | 0.4 |
|  | 1985 | 5.8 | 12.6 | 23.2 | 0.6 | 1.1 | 6.6 | 12.4 | 13.3 | 29.9 | 26.1 | 7.7 | 13.5 | 9.8 | 7.6 | 0.7 |
|  | 1990 | 8.0 | 14.6 | 25.5 | 0.9 | 1.9 | 9.2 | 15.3 | 16.0 | 31.8 | 28.4 | 9.9 | 14.7 | 13.5 | 9.1 | 1.1 |
|  | 1995 | 10.6 | 16.5 | 27.5 | 1.3 | 3.2 | 12.3 | 17.9 | 19.0 | 33.5 | 31.0 | 12.6 | 15.9 | 17.6 | 10.7 | 1.6 |
|  | 2000 | 13.5 | 19.3 | 28.9 | 2.0 | 5.2 | 15.7 | 20.8 | 22.1 | 35.0 | 33.6 | 15.8 | 16.3 | 21.2 | 12.4 | 2.3 |
|  | 2005 | 16.8 | 22.5 | 29.8 | 3.1 | 8.0 | 19.1 | 24.2 | 25.4 | 36.2 | 35.8 | 19.6 | 16.9 | 24.6 | 13.9 | 3.2 |
|  | 2010 | 20.3 | 26.6 | 30.0 | 4.6 | 11.5 | 22.4 | 27.8 | 28.6 | 37.1 | 37.5 | 23.5 | 17.2 | 27.5 | 16.2 | 4.4 |
|  | 2015 | 24.2 | 31.7 | 30.5 | 6.9 | 15.6 | 25.6 | 31.5 | 31.3 | 37.6 | 39.1 | 27.8 | 16.7 | 30.7 | 19.5 | 6.0 |
|  | 2020 ^a^ | 28.7 | 35.8 | 30.1 | 10.2 | 28.6 | 30.1 | 35.1 | 35.2 | 37.9 | 40.9 | 32.8 | 16.1 | 34.9 | 21.5 | 9.8 |
|  | 2025 ^a^ | 33.4 | 41.0 | 29.4 | 15.1 | 44.9 | 34.3 | 39.0 | 38.8 | 37.8 | 42.5 | 38.1 | 15.1 | 38.6 | 24.3 | 14.6 |
|  | 2030 ^a^ | 38.6 | 46.6 | 28.3 | 22.4 | 70.6 | 38.9 | 43.1 | 42.6 | 37.5 | 43.9 | 43.9 | 13.8 | 42.4 | 27.3 | 21.5 |
|  |  |  |  |  |  |  |  |  |  |  |  |  |  |  |  |  |
| **Girls** | 1975 | 5.2 | 7.3 | 18.6 | 0.4 | 0.8 | 5.5 | 10.7 | 10.2 | 22.3 | 18.3 | 7.4 | 7.4 | 9.3 | 7.3 | 1.5 |
|  | 1980 | 6.9 | 8.7 | 20.3 | 0.5 | 1.1 | 7.3 | 13.5 | 12.3 | 24.3 | 20.0 | 9.4 | 8.6 | 11.1 | 8.6 | 2.1 |
|  | 1985 | 9.1 | 10.1 | 22.0 | 0.7 | 1.7 | 9.5 | 16.4 | 14.8 | 25.9 | 21.5 | 11.6 | 9.2 | 14.0 | 10.1 | 3.0 |
|  | 1990 | 11.8 | 11.5 | 23.5 | 1.0 | 2.6 | 12.0 | 19.2 | 17.4 | 27.2 | 23.1 | 14.1 | 9.6 | 17.8 | 11.7 | 4.1 |
|  | 1995 | 14.9 | 12.8 | 24.6 | 1.5 | 4.0 | 14.9 | 21.4 | 20.2 | 28.3 | 25.2 | 17.0 | 10.2 | 21.6 | 13.0 | 5.6 |
|  | 2000 | 18.3 | 14.7 | 25.4 | 2.0 | 5.8 | 17.9 | 23.7 | 23.0 | 29.5 | 27.7 | 20.0 | 10.7 | 24.5 | 14.3 | 7.6 |
|  | 2005 | 21.8 | 17.0 | 25.7 | 2.9 | 8.1 | 20.7 | 26.1 | 25.7 | 30.5 | 30.0 | 23.3 | 11.3 | 26.7 | 15.4 | 9.9 |
|  | 2010 | 25.3 | 19.8 | 25.7 | 4.1 | 10.7 | 22.9 | 28.6 | 28.0 | 31.3 | 31.9 | 26.6 | 11.8 | 28.5 | 16.8 | 12.6 |
|  | 2015 | 28.9 | 23.2 | 25.9 | 5.7 | 13.6 | 24.6 | 31.3 | 29.8 | 32.0 | 33.7 | 30.0 | 11.8 | 30.3 | 18.7 | 15.7 |
|  | 2020 ^a^ | 33.4 | 27.1 | 25.4 | 7.1 | 17.0 | 27.8 | 33.4 | 32.6 | 33.0 | 36.5 | 34.0 | 12.4 | 32.5 | 19.6 | 20.0 |
|  | 2025 ^a^ | 37.8 | 31.7 | 24.6 | 8.8 | 20.8 | 30.4 | 35.6 | 35.0 | 33.9 | 39.0 | 38.0 | 12.7 | 34.0 | 20.9 | 25.3 |
|  | 2030 ^a^ | 42.6 | 37.3 | 23.6 | 10.8 | 25.0 | 32.9 | 37.8 | 37.3 | 34.9 | 41.6 | 42.2 | 13.0 | 35.3 | 22.3 | 31.8 |
|  |  | Kiribati | Kuwait | Kyrgyzstan | Lao People's Democratic Republic | Latvia | Lebanon | Lesotho | Liberia | Libya | Lithuania | Luxembourg | Madagascar | Malawi | Malaysia | Maldives |
| **Boys** | 1975 | 7.2 | 18.7 | 2.9 | 0.4 | 7.7 | 8.2 | 0.2 | 0.4 | 6.2 | 5.4 | 13.2 | 0.4 | 0.3 | 1.7 | 0.5 |
|  | 1980 | 10.3 | 22.7 | 3.5 | 0.6 | 9.4 | 11.7 | 0.2 | 0.6 | 10.1 | 7.3 | 15.8 | 0.6 | 0.4 | 2.5 | 0.9 |
|  | 1985 | 13.9 | 26.9 | 4.4 | 0.9 | 11.3 | 15.7 | 0.3 | 1.1 | 14.1 | 9.6 | 18.4 | 0.9 | 0.6 | 3.8 | 1.5 |
|  | 1990 | 18.3 | 30.0 | 5.5 | 1.4 | 13.0 | 19.7 | 0.5 | 2.0 | 17.3 | 11.9 | 20.8 | 1.4 | 0.9 | 5.9 | 2.4 |
|  | 1995 | 23.5 | 32.8 | 6.7 | 2.4 | 14.8 | 23.2 | 0.8 | 2.6 | 19.9 | 13.9 | 23.2 | 2.1 | 1.4 | 8.9 | 3.6 |
|  | 2000 | 30.1 | 36.0 | 8.2 | 3.9 | 16.2 | 26.5 | 1.4 | 2.9 | 22.6 | 15.5 | 25.0 | 3.0 | 2.2 | 13.1 | 5.3 |
|  | 2005 | 37.1 | 38.9 | 9.9 | 6.4 | 17.7 | 29.9 | 2.3 | 3.6 | 25.9 | 17.0 | 26.4 | 4.1 | 3.1 | 18.2 | 8.1 |
|  | 2010 | 43.4 | 41.7 | 12.2 | 9.8 | 20.2 | 32.6 | 3.6 | 4.6 | 29.4 | 19.2 | 27.2 | 5.6 | 4.3 | 23.4 | 12.4 |
|  | 2015 | 49.5 | 44.2 | 15.4 | 14.5 | 23.8 | 35.3 | 5.7 | 5.9 | 33.1 | 22.3 | 27.8 | 7.5 | 6.0 | 28.9 | 18.1 |
|  | 2020 ^a^ | 58.6 | 48.2 | 18.3 | 24.8 | 25.3 | 37.9 | 8.2 | 8.7 | 35.9 | 23.7 | 28.4 | 12.1 | 9.1 | 36.4 | 22.5 |
|  | 2025 ^a^ | 67.1 | 51.8 | 22.5 | 39.7 | 27.9 | 40.2 | 12.3 | 12.3 | 39.1 | 25.7 | 28.5 | 18.1 | 13.2 | 44.2 | 28.6 |
|  | 2030 ^a^ | 76.3 | 54.4 | 27.6 | 62.7 | 30.6 | 42.3 | 18.4 | 17.7 | 42.3 | 27.7 | 28.2 | 25.9 | 19.4 | 52.8 | 35.4 |
|  |  |  |  |  |  |  |  |  |  |  |  |  |  |  |  |  |
| **Girls** | 1975 | 15.0 | 23.4 | 4.9 | 0.6 | 7.3 | 10.9 | 1.3 | 2.1 | 9.4 | 5.3 | 14.2 | 1.3 | 1.2 | 2.1 | 0.7 |
|  | 1980 | 19.8 | 27.1 | 5.8 | 0.8 | 8.6 | 14.4 | 1.9 | 2.9 | 13.8 | 6.9 | 16.2 | 1.9 | 1.7 | 3.0 | 1.1 |
|  | 1985 | 24.7 | 30.6 | 6.9 | 1.2 | 10.0 | 18.0 | 2.6 | 4.3 | 17.8 | 8.7 | 18.1 | 2.7 | 2.5 | 4.2 | 1.7 |
|  | 1990 | 30.1 | 32.9 | 8.1 | 1.8 | 11.1 | 21.4 | 3.7 | 6.5 | 20.7 | 10.4 | 19.7 | 3.8 | 3.6 | 5.8 | 2.5 |
|  | 1995 | 36.0 | 34.7 | 9.3 | 2.6 | 12.3 | 24.0 | 5.5 | 7.9 | 22.8 | 11.8 | 21.2 | 5.2 | 5.0 | 8.2 | 3.5 |
|  | 2000 | 42.7 | 36.4 | 10.6 | 3.9 | 13.2 | 26.0 | 8.2 | 8.7 | 24.8 | 12.8 | 22.4 | 6.8 | 6.9 | 11.3 | 4.8 |
|  | 2005 | 49.2 | 37.8 | 12.0 | 5.7 | 14.2 | 27.6 | 12.2 | 10.1 | 26.9 | 13.9 | 23.2 | 8.6 | 9.0 | 15.0 | 6.9 |
|  | 2010 | 54.5 | 38.9 | 13.6 | 8.0 | 15.9 | 28.6 | 17.3 | 11.9 | 28.9 | 15.4 | 23.8 | 10.6 | 11.4 | 18.7 | 9.8 |
|  | 2015 | 59.2 | 39.7 | 15.7 | 10.9 | 18.0 | 29.7 | 23.4 | 13.9 | 31.1 | 17.4 | 24.1 | 13.0 | 14.2 | 22.4 | 13.4 |
|  | 2020 ^a^ | 66.5 | 39.5 | 16.8 | 16.8 | 18.5 | 29.7 | 35.6 | 15.2 | 31.6 | 18.5 | 24.3 | 15.6 | 18.3 | 28.1 | 21.0 |
|  | 2025 ^a^ | 72.8 | 39.3 | 18.4 | 24.4 | 19.7 | 29.5 | 51.4 | 16.8 | 32.3 | 20.0 | 24.1 | 18.4 | 23.1 | 34.3 | 31.1 |
|  | 2030 ^a^ | 79.1 | 38.6 | 20.0 | 35.4 | 21.0 | 28.7 | 74.1 | 18.5 | 32.8 | 21.4 | 23.7 | 21.5 | 29.3 | 42.0 | 46.4 |
|  |  | Mali | Malta | Marshall Islands | Mauritania | Mauritius | Mexico | Micronesia (Federated States of) | Mongolia | Montenegro | Morocco | Mozambique | Myanmar | Namibia | Nauru | Nepal |
| **Boys** | 1975 | 0.3 | 27.6 | 15.4 | 0.3 | 1.5 | 10.3 | 6.9 | 3.3 | 3.0 | 2.9 | 0.3 | 0.6 | 0.4 | 36.0 | 0.2 |
|  | 1980 | 0.4 | 29.1 | 19.1 | 0.5 | 1.8 | 13.2 | 9.3 | 4.2 | 4.3 | 4.0 | 0.5 | 0.8 | 0.5 | 38.9 | 0.3 |
|  | 1985 | 0.6 | 31.6 | 22.7 | 1.0 | 2.3 | 16.4 | 12.2 | 5.5 | 6.4 | 5.8 | 0.9 | 1.2 | 0.7 | 41.9 | 0.5 |
|  | 1990 | 0.9 | 33.6 | 27.8 | 1.7 | 3.1 | 19.7 | 16.0 | 6.9 | 9.1 | 8.2 | 1.6 | 1.8 | 0.9 | 45.2 | 0.9 |
|  | 1995 | 1.4 | 35.2 | 33.0 | 2.6 | 4.1 | 23.2 | 20.4 | 8.2 | 12.5 | 11.2 | 2.5 | 2.7 | 1.4 | 48.6 | 1.4 |
|  | 2000 | 2.1 | 36.8 | 38.0 | 3.7 | 5.3 | 26.6 | 25.2 | 9.4 | 16.3 | 14.5 | 3.6 | 4.0 | 2.2 | 51.9 | 2.2 |
|  | 2005 | 3.2 | 38.0 | 43.5 | 5.1 | 6.7 | 29.9 | 31.0 | 10.8 | 20.6 | 18.1 | 4.7 | 5.9 | 3.6 | 55.2 | 3.4 |
|  | 2010 | 4.6 | 38.9 | 49.0 | 6.8 | 8.3 | 32.8 | 37.8 | 13.1 | 24.7 | 21.8 | 6.0 | 8.7 | 5.9 | 58.4 | 4.9 |
|  | 2015 | 6.4 | 39.4 | 54.1 | 8.7 | 10.5 | 35.2 | 45.0 | 16.1 | 28.9 | 26.0 | 7.6 | 12.4 | 9.1 | 61.3 | 6.8 |
|  | 2020 ^a^ | 10.6 | 40.0 | 60.6 | 10.7 | 13.5 | 38.6 | 53.0 | 18.0 | 34.5 | 30.9 | 14.1 | 18.6 | 12.5 | 64.7 | 8.5 |
|  | 2025 ^a^ | 15.5 | 40.1 | 67.0 | 13.1 | 17.3 | 41.5 | 61.7 | 20.6 | 40.2 | 36.0 | 21.7 | 27.1 | 18.4 | 67.9 | 10.7 |
|  | 2030 ^a^ | 23.6 | 40.1 | 73.5 | 15.7 | 22.1 | 44.3 | 71.1 | 23.5 | 46.2 | 41.6 | 33.2 | 39.7 | 26.6 | 71.2 | 13.2 |
|  |  |  |  |  |  |  |  |  |  |  |  |  |  |  |  |  |
| **Girls** | 1975 | 1.4 | 25.2 | 25.7 | 1.5 | 4.0 | 12.4 | 14.2 | 6.3 | 2.4 | 5.3 | 1.4 | 0.8 | 1.5 | 47.6 | 0.3 |
|  | 1980 | 1.9 | 26.0 | 30.3 | 2.4 | 4.9 | 15.0 | 17.9 | 7.8 | 3.4 | 6.9 | 2.1 | 1.1 | 2.0 | 50.0 | 0.5 |
|  | 1985 | 2.7 | 27.4 | 34.5 | 3.8 | 5.9 | 17.9 | 21.9 | 9.4 | 4.9 | 9.1 | 3.3 | 1.5 | 2.7 | 52.5 | 0.7 |
|  | 1990 | 3.8 | 28.6 | 39.9 | 5.6 | 7.4 | 20.8 | 26.7 | 11.1 | 6.7 | 11.7 | 5.2 | 2.1 | 3.7 | 55.3 | 1.1 |
|  | 1995 | 5.2 | 29.7 | 45.2 | 7.7 | 9.1 | 23.9 | 32.0 | 12.5 | 8.9 | 14.7 | 7.4 | 2.9 | 5.1 | 58.1 | 1.6 |
|  | 2000 | 7.0 | 30.9 | 49.9 | 10.0 | 11.0 | 27.0 | 37.2 | 13.7 | 11.4 | 17.6 | 9.7 | 4.0 | 7.3 | 60.7 | 2.4 |
|  | 2005 | 9.2 | 32.0 | 54.6 | 12.4 | 13.0 | 29.7 | 43.2 | 15.1 | 14.0 | 20.5 | 12.0 | 5.4 | 10.5 | 63.3 | 3.6 |
|  | 2010 | 11.6 | 32.8 | 58.9 | 14.7 | 15.2 | 32.2 | 49.3 | 16.8 | 16.5 | 23.3 | 14.3 | 7.1 | 14.7 | 65.7 | 5.1 |
|  | 2015 | 14.2 | 33.5 | 62.8 | 17.0 | 17.7 | 34.7 | 55.2 | 18.5 | 19.0 | 26.4 | 16.7 | 9.3 | 19.8 | 67.8 | 7.2 |
|  | 2020 ^a^ | 18.7 | 34.5 | 68.4 | 20.2 | 22.3 | 37.5 | 62.4 | 19.9 | 22.4 | 29.7 | 19.8 | 13.4 | 28.3 | 70.2 | 11.6 |
|  | 2025 ^a^ | 23.7 | 35.3 | 73.2 | 23.3 | 27.1 | 40.0 | 69.6 | 21.3 | 25.8 | 33.5 | 22.9 | 18.5 | 39.2 | 72.5 | 17.6 |
|  | 2030 ^a^ | 30.0 | 35.9 | 77.9 | 26.5 | 33.0 | 42.5 | 77.1 | 22.8 | 29.3 | 37.8 | 26.2 | 25.1 | 54.3 | 74.8 | 26.2 |
|  |  | Netherlands | New Zealand | Nicaragua | Niger | Nigeria | Niue | Norway | Oman | Pakistan | Palau | Panama | Papua New Guinea | Paraguay | Peru | Philippines |
| **Boys** | 1975 | 8.3 | 19.9 | 5.8 | 0.2 | 0.3 | 6.8 | 10.5 | 2.8 | 0.6 | 18.3 | 5.7 | 3.0 | 3.7 | 7.5 | 1.0 |
|  | 1980 | 10.1 | 21.9 | 7.2 | 0.3 | 0.4 | 9.6 | 13.7 | 4.8 | 0.8 | 21.9 | 7.0 | 4.1 | 4.9 | 9.4 | 1.4 |
|  | 1985 | 12.2 | 24.0 | 9.0 | 0.4 | 0.6 | 13.9 | 16.6 | 8.2 | 1.3 | 26.8 | 8.8 | 5.6 | 6.6 | 11.4 | 2.2 |
|  | 1990 | 14.6 | 26.5 | 11.2 | 0.7 | 0.9 | 19.1 | 19.3 | 13.0 | 1.9 | 32.5 | 11.0 | 7.5 | 9.2 | 13.5 | 3.6 |
|  | 1995 | 17.5 | 29.7 | 13.7 | 1.0 | 1.4 | 24.7 | 22.2 | 18.0 | 2.7 | 38.0 | 13.8 | 9.9 | 12.3 | 15.7 | 5.2 |
|  | 2000 | 20.1 | 32.8 | 16.4 | 1.5 | 2.1 | 31.4 | 24.8 | 21.9 | 3.8 | 42.6 | 17.2 | 12.7 | 15.7 | 18.1 | 6.8 |
|  | 2005 | 22.3 | 35.4 | 19.4 | 2.3 | 3.0 | 38.9 | 26.6 | 25.4 | 5.3 | 48.4 | 20.4 | 16.3 | 19.5 | 20.5 | 8.7 |
|  | 2010 | 24.0 | 37.4 | 22.4 | 3.3 | 4.4 | 46.9 | 27.6 | 28.8 | 7.1 | 54.7 | 23.3 | 20.3 | 23.6 | 23.0 | 11.1 |
|  | 2015 | 25.3 | 39.5 | 25.9 | 4.7 | 6.2 | 54.6 | 28.6 | 32.9 | 9.6 | 59.6 | 26.3 | 25.0 | 28.0 | 25.6 | 14.0 |
|  | 2020 ^a^ | 27.6 | 42.6 | 29.7 | 7.5 | 9.8 | 64.6 | 29.6 | 37.2 | 14.6 | 66.0 | 30.7 | 30.0 | 33.4 | 28.3 | 16.8 |
|  | 2025 ^a^ | 29.3 | 45.2 | 33.7 | 11.4 | 14.4 | 74.7 | 30.0 | 41.1 | 21.1 | 72.2 | 34.8 | 35.6 | 38.9 | 31.1 | 20.1 |
|  | 2030 ^a^ | 30.9 | 47.8 | 37.9 | 17.6 | 21.6 | 85.6 | 30.1 | 45.1 | 29.9 | 78.5 | 39.2 | 41.7 | 44.9 | 34.1 | 23.6 |
|  |  |  |  |  |  |  |  |  |  |  |  |  |  |  |  |  |
| **Girls** | 1975 | 10.1 | 22.2 | 8.9 | 1.0 | 1.0 | 13.6 | 12.1 | 4.7 | 0.7 | 28.6 | 8.7 | 8.8 | 6.0 | 10.9 | 1.2 |
|  | 1980 | 11.7 | 23.9 | 10.9 | 1.4 | 1.4 | 17.8 | 14.7 | 7.3 | 1.0 | 32.7 | 10.5 | 11.1 | 7.4 | 13.1 | 1.7 |
|  | 1985 | 13.6 | 25.6 | 13.2 | 2.0 | 2.0 | 23.3 | 16.9 | 11.0 | 1.4 | 37.8 | 12.7 | 14.0 | 9.3 | 15.3 | 2.4 |
|  | 1990 | 15.6 | 27.6 | 15.9 | 2.8 | 2.8 | 29.1 | 18.9 | 15.6 | 1.9 | 43.4 | 15.2 | 17.2 | 11.8 | 17.4 | 3.6 |
|  | 1995 | 17.8 | 30.2 | 18.9 | 3.8 | 3.9 | 35.2 | 21.0 | 19.8 | 2.6 | 48.5 | 18.5 | 20.7 | 14.6 | 19.5 | 4.8 |
|  | 2000 | 19.8 | 32.7 | 22.0 | 5.0 | 5.1 | 41.6 | 22.9 | 22.8 | 3.5 | 52.6 | 22.1 | 24.3 | 17.4 | 21.6 | 5.9 |
|  | 2005 | 21.6 | 34.8 | 25.0 | 6.6 | 6.5 | 48.2 | 24.1 | 25.0 | 4.7 | 57.4 | 25.4 | 28.3 | 20.3 | 23.5 | 7.2 |
|  | 2010 | 22.9 | 36.6 | 28.0 | 8.5 | 8.1 | 54.7 | 25.0 | 27.1 | 6.4 | 62.3 | 28.4 | 32.5 | 23.2 | 25.4 | 8.6 |
|  | 2015 | 23.7 | 38.5 | 31.3 | 10.9 | 9.9 | 60.7 | 25.8 | 29.8 | 8.6 | 66.0 | 31.4 | 36.8 | 26.3 | 27.5 | 10.5 |
|  | 2020 ^a^ | 25.4 | 40.9 | 35.0 | 15.7 | 12.5 | 66.4 | 26.3 | 31.4 | 10.4 | 70.5 | 35.7 | 41.8 | 29.9 | 29.5 | 12.1 |
|  | 2025 ^a^ | 26.6 | 43.0 | 38.6 | 21.3 | 15.4 | 72.5 | 26.5 | 32.8 | 12.8 | 74.6 | 39.7 | 46.8 | 33.5 | 31.6 | 14.0 |
|  | 2030 ^a^ | 27.7 | 45.1 | 42.4 | 28.9 | 18.9 | 78.6 | 26.5 | 33.9 | 15.4 | 78.5 | 43.8 | 52.0 | 37.4 | 33.7 | 16.1 |
|  |  | Poland | Portugal | Qatar | Republic of Korea | Republic of Moldova | Republic of North Macedonia | Romania | Russian Federation | Rwanda | Saint Kitts and Nevis | Saint Lucia | Saint Vincent and the Grenadines | Samoa | Sao Tome and Principe | Saudi Arabia |
| **Boys** | 1975 | 6.7 | 8.2 | 18.4 | 5.9 | 2.8 | 6.0 | 4.4 | 7.6 | 0.2 | 3.9 | 2.4 | 3.2 | 6.4 | 0.6 | 5.8 |
|  | 1980 | 8.5 | 10.6 | 20.2 | 8.6 | 3.9 | 7.8 | 6.1 | 9.2 | 0.3 | 4.9 | 3.3 | 4.4 | 8.5 | 0.8 | 9.1 |
|  | 1985 | 10.9 | 13.8 | 22.9 | 12.1 | 5.5 | 10.0 | 7.6 | 11.3 | 0.4 | 6.3 | 4.5 | 6.1 | 11.3 | 1.1 | 13.1 |
|  | 1990 | 13.2 | 17.5 | 25.6 | 16.2 | 7.3 | 12.5 | 10.1 | 13.0 | 0.7 | 8.1 | 6.3 | 8.5 | 15.0 | 1.8 | 17.4 |
|  | 1995 | 15.5 | 21.5 | 29.6 | 20.2 | 9.1 | 15.4 | 12.6 | 14.5 | 1.1 | 10.5 | 8.6 | 11.3 | 19.8 | 2.7 | 21.3 |
|  | 2000 | 17.9 | 25.6 | 32.1 | 23.4 | 10.7 | 18.3 | 15.3 | 15.3 | 1.7 | 13.3 | 11.1 | 14.7 | 25.7 | 3.9 | 25.0 |
|  | 2005 | 21.0 | 29.2 | 36.1 | 26.9 | 12.5 | 21.2 | 18.0 | 16.6 | 2.6 | 16.9 | 13.8 | 18.6 | 32.4 | 5.4 | 29.1 |
|  | 2010 | 24.9 | 31.5 | 38.3 | 29.3 | 14.9 | 24.9 | 22.6 | 19.6 | 3.8 | 21.3 | 16.8 | 22.9 | 39.1 | 7.2 | 33.3 |
|  | 2015 | 29.9 | 32.9 | 40.7 | 32.1 | 18.6 | 29.6 | 28.0 | 23.6 | 4.9 | 26.6 | 21.3 | 27.7 | 46.3 | 9.3 | 37.5 |
|  | 2020 ^a^ | 33.6 | 36.7 | 44.2 | 35.1 | 20.6 | 33.4 | 32.2 | 32.2 | 6.4 | 34.7 | 25.1 | 33.3 | 55.5 | 14.6 | 46.0 |
|  | 2025 ^a^ | 38.3 | 39.2 | 47.2 | 37.6 | 23.6 | 37.9 | 37.6 | 40.7 | 8.0 | 44.2 | 29.7 | 39.2 | 65.0 | 21.0 | 51.0 |
|  | 2030 ^a^ | 43.4 | 41.5 | 50.2 | 40.0 | 26.8 | 42.8 | 43.5 | 51.6 | 9.9 | 56.3 | 34.6 | 45.6 | 75.3 | 29.5 | 56.0 |
|  |  |  |  |  |  |  |  |  |  |  |  |  |  |  |  |  |
| **Girls** | 1975 | 5.3 | 9.1 | 22.1 | 5.1 | 2.9 | 4.7 | 3.7 | 7.1 | 1.2 | 6.0 | 4.3 | 5.4 | 13.1 | 2.4 | 8.5 |
|  | 1980 | 6.5 | 11.2 | 23.4 | 7.0 | 3.9 | 6.0 | 4.9 | 8.5 | 1.7 | 7.4 | 5.5 | 6.9 | 16.4 | 3.1 | 12.1 |
|  | 1985 | 7.9 | 14.0 | 25.0 | 9.2 | 5.2 | 7.4 | 6.2 | 10.0 | 2.4 | 9.0 | 7.1 | 9.0 | 20.4 | 4.2 | 16.0 |
|  | 1990 | 9.2 | 17.0 | 27.4 | 11.6 | 6.6 | 9.0 | 7.9 | 11.2 | 3.5 | 10.9 | 9.2 | 11.7 | 25.1 | 5.8 | 19.7 |
|  | 1995 | 10.6 | 20.3 | 29.6 | 13.8 | 8.0 | 10.7 | 9.7 | 12.1 | 5.0 | 13.3 | 11.6 | 14.6 | 30.9 | 7.8 | 22.7 |
|  | 2000 | 12.0 | 23.9 | 31.4 | 15.6 | 9.2 | 12.6 | 11.4 | 12.5 | 7.3 | 16.0 | 14.2 | 17.7 | 37.4 | 9.8 | 25.3 |
|  | 2005 | 14.0 | 27.2 | 33.3 | 17.3 | 10.6 | 14.5 | 13.2 | 13.4 | 9.9 | 19.2 | 16.7 | 21.1 | 44.0 | 12.1 | 27.6 |
|  | 2010 | 16.3 | 29.7 | 34.6 | 18.5 | 12.4 | 17.0 | 15.9 | 15.3 | 13.1 | 22.9 | 19.6 | 24.5 | 50.4 | 14.3 | 29.7 |
|  | 2015 | 19.4 | 31.4 | 35.3 | 20.1 | 14.9 | 20.1 | 19.0 | 17.6 | 16.2 | 27.1 | 23.6 | 28.3 | 56.6 | 16.3 | 31.9 |
|  | 2020 ^a^ | 21.6 | 35.1 | 37.1 | 21.2 | 16.1 | 22.5 | 21.5 | 18.2 | 20.5 | 33.1 | 26.9 | 32.7 | 65.2 | 19.3 | 33.1 |
|  | 2025 ^a^ | 24.4 | 37.9 | 38.3 | 22.2 | 18.0 | 25.5 | 24.6 | 19.7 | 24.9 | 40.1 | 30.8 | 37.1 | 73.5 | 22.3 | 34.2 |
|  | 2030 ^a^ | 27.6 | 40.6 | 39.4 | 23.1 | 19.9 | 28.8 | 27.9 | 21.3 | 29.8 | 48.7 | 35.1 | 41.8 | 82.2 | 25.4 | 35.0 |
|  |  | Senegal | Serbia | Seychelles | Sierra Leone | Singapore | Slovakia | Slovenia | Solomon Islands | Somalia | South Africa | Spain | Sri Lanka | Sudan (former) | Suriname | Sweden |
| **Boys** | 1975 | 0.5 | 5.0 | 2.4 | 0.4 | 22.4 | 4.6 | 4.7 | 1.9 | 0.5 | 1.4 | 17.3 | 0.6 | 0.4 | 6.1 | 13.2 |
|  | 1980 | 0.7 | 6.8 | 3.5 | 0.6 | 22.6 | 6.3 | 6.7 | 2.4 | 0.8 | 1.3 | 20.7 | 0.8 | 0.6 | 9.3 | 15.3 |
|  | 1985 | 1.0 | 9.0 | 4.8 | 0.9 | 23.4 | 8.4 | 8.9 | 3.4 | 1.1 | 1.3 | 23.9 | 1.1 | 0.9 | 12.8 | 17.2 |
|  | 1990 | 1.4 | 11.6 | 6.2 | 1.3 | 23.9 | 10.5 | 11.2 | 4.7 | 1.7 | 1.5 | 26.7 | 1.7 | 1.5 | 15.4 | 19.1 |
|  | 1995 | 1.9 | 14.5 | 8.1 | 1.8 | 25.0 | 12.6 | 13.5 | 6.3 | 2.4 | 2.1 | 29.1 | 2.6 | 2.4 | 18.3 | 21.1 |
|  | 2000 | 2.5 | 18.0 | 10.6 | 2.5 | 25.7 | 15.0 | 16.5 | 8.1 | 3.4 | 3.3 | 31.5 | 3.9 | 3.4 | 21.0 | 22.7 |
|  | 2005 | 3.2 | 22.0 | 13.5 | 3.4 | 26.0 | 17.9 | 20.0 | 10.3 | 4.7 | 6.1 | 33.7 | 5.9 | 4.6 | 24.3 | 23.4 |
|  | 2010 | 4.2 | 26.5 | 16.7 | 4.6 | 26.2 | 21.6 | 24.6 | 12.8 | 6.2 | 11.1 | 35.3 | 8.9 | 5.9 | 28.1 | 23.8 |
|  | 2015 | 5.5 | 31.5 | 20.3 | 6.0 | 26.6 | 26.7 | 29.9 | 16.0 | 8.1 | 18.6 | 36.8 | 13.0 | 7.7 | 31.4 | 25.0 |
|  | 2020 ^a^ | 7.8 | 36.9 | 24.3 | 8.8 | 26.9 | 30.2 | 34.5 | 19.2 | 12.6 | 32.9 | 38.1 | 19.3 | 9.6 | 34.5 | 25.1 |
|  | 2025 ^a^ | 10.6 | 42.7 | 28.8 | 12.3 | 27.0 | 34.9 | 40.1 | 22.8 | 18.1 | 48.1 | 39.0 | 28.4 | 11.6 | 38.0 | 25.2 |
|  | 2030 ^a^ | 14.4 | 49.1 | 33.6 | 17.1 | 27.1 | 40.0 | 46.2 | 26.7 | 25.6 | 67.6 | 39.7 | 42.0 | 13.9 | 41.5 | 24.9 |
|  |  |  |  |  |  |  |  |  |  |  |  |  |  |  |  |  |
| **Girls** | 1975 | 2.2 | 4.0 | 4.5 | 2.1 | 17.3 | 3.5 | 4.2 | 6.2 | 2.2 | 3.7 | 15.5 | 0.8 | 1.7 | 8.8 | 15.2 |
|  | 1980 | 3.0 | 5.2 | 6.5 | 2.8 | 17.1 | 4.8 | 5.7 | 7.9 | 2.9 | 3.8 | 17.7 | 1.1 | 2.4 | 12.4 | 16.5 |
|  | 1985 | 3.9 | 6.6 | 8.3 | 3.8 | 17.1 | 6.3 | 7.4 | 10.3 | 4.0 | 4.2 | 19.7 | 1.4 | 3.4 | 15.8 | 17.5 |
|  | 1990 | 5.0 | 8.2 | 10.2 | 5.0 | 17.2 | 7.7 | 8.9 | 13.2 | 5.3 | 4.9 | 21.3 | 2.0 | 4.9 | 18.2 | 18.5 |
|  | 1995 | 6.2 | 9.9 | 12.4 | 6.4 | 17.5 | 9.0 | 10.6 | 16.2 | 7.0 | 6.3 | 22.8 | 2.9 | 6.9 | 20.5 | 19.6 |
|  | 2000 | 7.6 | 12.1 | 15.0 | 8.1 | 17.7 | 10.5 | 12.6 | 19.3 | 9.1 | 8.9 | 24.7 | 4.0 | 9.0 | 22.6 | 20.4 |
|  | 2005 | 9.2 | 14.6 | 17.8 | 10.1 | 17.6 | 12.4 | 15.2 | 22.5 | 11.4 | 13.5 | 26.7 | 5.7 | 11.2 | 25.0 | 20.8 |
|  | 2010 | 11.0 | 17.7 | 20.6 | 12.3 | 17.6 | 14.7 | 18.5 | 25.7 | 13.9 | 20.0 | 28.7 | 7.9 | 13.5 | 27.4 | 21.1 |
|  | 2015 | 13.0 | 21.2 | 23.7 | 14.7 | 17.8 | 17.9 | 22.4 | 29.2 | 16.7 | 27.6 | 30.6 | 11.0 | 16.2 | 29.6 | 22.0 |
|  | 2020 ^a^ | 15.1 | 24.7 | 26.9 | 17.4 | 17.5 | 20.0 | 25.7 | 33.0 | 19.9 | 35.1 | 32.3 | 15.4 | 19.3 | 31.1 | 22.1 |
|  | 2025 ^a^ | 17.4 | 28.7 | 30.4 | 20.3 | 17.4 | 22.9 | 29.8 | 36.9 | 23.4 | 44.4 | 34.1 | 21.4 | 22.6 | 32.7 | 22.2 |
|  | 2030 ^a^ | 19.8 | 33.0 | 34.1 | 23.5 | 17.3 | 26.0 | 34.2 | 41.0 | 27.1 | 55.1 | 35.9 | 29.7 | 26.1 | 34.1 | 22.3 |
|  |  | Switzerland | Syrian Arab Republic | Tajikistan | Thailand | Timor-Leste | Togo | Tonga | Trinidad and Tobago | Tunisia | Turkey | Turkmenistan | Tuvalu | Uganda | Ukraine | United Arab Emirates |
| **Boys** | 1975 | 7.3 | 4.2 | 2.7 | 1.0 | 0.4 | 0.4 | 7.3 | 1.6 | 3.2 | 3.3 | 4.3 | 6.3 | 0.2 | 6.3 | 14.2 |
|  | 1980 | 8.7 | 5.4 | 3.1 | 1.6 | 0.6 | 0.5 | 9.7 | 2.1 | 4.3 | 4.5 | 4.8 | 9.2 | 0.3 | 7.9 | 16.2 |
|  | 1985 | 11.5 | 7.1 | 3.7 | 2.5 | 1.0 | 0.7 | 13.2 | 3.0 | 5.6 | 6.7 | 5.6 | 13.2 | 0.5 | 9.9 | 18.6 |
|  | 1990 | 15.8 | 9.2 | 4.6 | 3.8 | 1.6 | 1.1 | 17.9 | 4.3 | 7.6 | 10.1 | 6.7 | 19.0 | 0.7 | 12.0 | 19.9 |
|  | 1995 | 19.3 | 11.9 | 5.6 | 5.7 | 2.7 | 1.6 | 23.6 | 6.2 | 10.0 | 13.6 | 8.0 | 25.7 | 1.1 | 13.9 | 22.6 |
|  | 2000 | 20.8 | 15.2 | 6.8 | 8.4 | 4.2 | 2.3 | 29.8 | 8.9 | 12.7 | 17.2 | 9.7 | 32.1 | 1.7 | 15.5 | 25.8 |
|  | 2005 | 21.7 | 19.2 | 8.3 | 12.4 | 6.4 | 3.3 | 37.4 | 12.4 | 15.8 | 21.2 | 11.7 | 38.8 | 2.3 | 17.3 | 29.1 |
|  | 2010 | 22.1 | 23.5 | 10.5 | 17.7 | 9.4 | 4.5 | 44.7 | 17.2 | 19.1 | 25.4 | 14.1 | 45.8 | 3.2 | 19.9 | 30.7 |
|  | 2015 | 22.6 | 28.0 | 13.4 | 23.7 | 13.2 | 6.2 | 51.9 | 23.2 | 23.2 | 29.5 | 17.3 | 52.7 | 4.4 | 23.6 | 36.6 |
|  | 2020 ^a^ | 22.5 | 33.6 | 15.5 | 36.8 | 22.9 | 9.1 | 61.9 | 32.5 | 27.6 | 34.8 | 20.3 | 61.7 | 7.0 | 25.5 | 39.5 |
|  | 2025 ^a^ | 21.6 | 39.5 | 18.8 | 54.5 | 35.8 | 13.0 | 71.8 | 44.5 | 32.3 | 40.2 | 23.8 | 70.4 | 10.4 | 28.4 | 43.7 |
|  | 2030 ^a^ | 20.3 | 46.0 | 22.8 | 81.1 | 55.5 | 18.2 | 82.5 | 60.8 | 37.5 | 45.8 | 27.6 | 79.6 | 15.6 | 31.4 | 48.2 |
|  |  |  |  |  |  |  |  |  |  |  |  |  |  |  |  |  |
| **Girls** | 1975 | 8.3 | 6.8 | 4.7 | 1.3 | 0.6 | 1.7 | 14.9 | 3.0 | 6.2 | 5.8 | 6.7 | 13.6 | 1.3 | 6.0 | 18.2 |
|  | 1980 | 9.6 | 8.5 | 5.3 | 1.9 | 0.9 | 2.3 | 18.9 | 3.7 | 7.9 | 7.4 | 7.5 | 18.1 | 1.8 | 7.3 | 19.8 |
|  | 1985 | 11.9 | 10.4 | 6.2 | 2.8 | 1.3 | 3.1 | 23.8 | 4.8 | 9.8 | 10.1 | 8.5 | 23.8 | 2.6 | 8.8 | 21.4 |
|  | 1990 | 15.3 | 12.6 | 7.2 | 4.0 | 1.9 | 4.2 | 29.8 | 6.3 | 12.2 | 13.7 | 9.6 | 30.9 | 3.8 | 10.3 | 22.8 |
|  | 1995 | 17.8 | 15.2 | 8.3 | 5.6 | 2.9 | 5.6 | 36.4 | 8.5 | 15.0 | 17.1 | 10.9 | 38.2 | 5.4 | 11.5 | 24.4 |
|  | 2000 | 18.9 | 18.0 | 9.4 | 7.7 | 4.0 | 7.2 | 43.2 | 11.4 | 17.7 | 20.1 | 12.4 | 44.7 | 7.2 | 12.6 | 26.5 |
|  | 2005 | 19.5 | 21.1 | 10.8 | 10.6 | 5.6 | 9.1 | 50.3 | 14.9 | 20.3 | 23.0 | 13.9 | 50.9 | 9.5 | 13.9 | 28.7 |
|  | 2010 | 19.9 | 24.1 | 12.6 | 14.4 | 7.6 | 11.2 | 56.5 | 19.1 | 22.7 | 25.6 | 15.6 | 56.8 | 12.2 | 15.6 | 30.4 |
|  | 2015 | 20.5 | 27.2 | 14.8 | 18.4 | 10.1 | 13.6 | 62.2 | 24.2 | 25.3 | 28.1 | 17.6 | 62.1 | 15.4 | 17.8 | 33.2 |
|  | 2020 ^a^ | 20.1 | 31.1 | 16.6 | 28.6 | 15.2 | 19.2 | 70.6 | 31.2 | 28.4 | 31.2 | 19.9 | 69.5 | 18.6 | 18.6 | 35.4 |
|  | 2025 ^a^ | 19.4 | 34.9 | 19.2 | 40.4 | 21.8 | 25.1 | 78.2 | 39.7 | 31.4 | 34.0 | 22.4 | 75.8 | 22.4 | 19.5 | 38.0 |
|  | 2030 ^a^ | 18.2 | 39.0 | 22.0 | 56.9 | 31.5 | 32.9 | 86.0 | 50.6 | 34.5 | 36.7 | 25.3 | 82.2 | 26.6 | 20.7 | 40.8 |
|  |  | United Kingdom of Great Britain and Northern Ireland | United Republic of Tanzania | United States of America | Uruguay | Uzbekistan | Vanuatu | Venezuela (Bolivarian Republic of) | Viet Nam | Yemen | Zambia | Zimbabwe |  |  |  |  |
| **Boys** | 1975 | 15.4 | 0.3 | 18.8 | 15.5 | 3.0 | 2.8 | 13.2 | 0.4 | 1.0 | 0.8 | 0.3 |  |  |  |  |
|  | 1980 | 17.2 | 0.4 | 21.2 | 17.1 | 3.7 | 3.7 | 15.7 | 0.4 | 1.5 | 1.2 | 0.4 |  |  |  |  |
|  | 1985 | 19.3 | 0.7 | 24.3 | 19.4 | 4.8 | 5.1 | 18.5 | 0.6 | 2.2 | 1.8 | 0.5 |  |  |  |  |
|  | 1990 | 21.7 | 1.2 | 28.1 | 21.6 | 5.9 | 7.1 | 21.5 | 0.8 | 3.5 | 2.4 | 0.9 |  |  |  |  |
|  | 1995 | 24.6 | 1.8 | 32.4 | 24.0 | 7.1 | 9.5 | 24.1 | 1.3 | 5.2 | 3.0 | 1.3 |  |  |  |  |
|  | 2000 | 27.1 | 2.6 | 36.5 | 26.8 | 8.5 | 12.3 | 26.6 | 2.1 | 7.6 | 3.7 | 2.0 |  |  |  |  |
|  | 2005 | 29.0 | 3.7 | 39.7 | 29.5 | 10.3 | 15.7 | 28.7 | 3.6 | 10.6 | 4.8 | 3.0 |  |  |  |  |
|  | 2010 | 30.0 | 5.2 | 41.9 | 32.2 | 12.4 | 19.6 | 30.7 | 6.1 | 14.1 | 6.3 | 4.4 |  |  |  |  |
|  | 2015 | 30.7 | 7.2 | 43.8 | 35.1 | 15.6 | 23.7 | 32.7 | 10.6 | 18.4 | 8.1 | 6.2 |  |  |  |  |
|  | 2020 ^a^ | 32.5 | 11.4 | 47.3 | 38.0 | 18.6 | 28.6 | 34.5 | 16.7 | 29.8 | 11.4 | 10.5 |  |  |  |  |
|  | 2025 ^a^ | 33.4 | 17.1 | 49.8 | 41.1 | 22.7 | 33.8 | 36.2 | 27.6 | 43.6 | 15.2 | 15.0 |  |  |  |  |
|  | 2030 ^a^ | 34.1 | 25.4 | 52.1 | 44.6 | 27.6 | 39.5 | 37.7 | 45.7 | 63.6 | 20.3 | 22.7 |  |  |  |  |
|  |  |  |  |  |  |  |  |  |  |  |  |  |  |  |  |  |
| **Girls** | 1975 | 18.0 | 1.3 | 18.9 | 18.6 | 5.1 | 8.8 | 16.6 | 0.5 | 2.2 | 2.8 | 1.7 |  |  |  |  |
|  | 1980 | 20.1 | 1.9 | 21.4 | 19.9 | 6.2 | 11.0 | 19.3 | 0.6 | 3.0 | 4.0 | 2.3 |  |  |  |  |
|  | 1985 | 22.4 | 2.9 | 24.3 | 21.6 | 7.4 | 13.9 | 22.1 | 0.8 | 4.2 | 5.3 | 3.3 |  |  |  |  |
|  | 1990 | 24.8 | 4.1 | 27.7 | 23.2 | 8.6 | 17.3 | 24.8 | 1.1 | 5.8 | 6.5 | 4.8 |  |  |  |  |
|  | 1995 | 27.4 | 5.7 | 31.3 | 24.8 | 9.8 | 21.0 | 27.3 | 1.5 | 8.0 | 7.8 | 6.8 |  |  |  |  |
|  | 2000 | 29.5 | 7.6 | 34.6 | 26.4 | 11.1 | 24.8 | 29.5 | 2.2 | 10.6 | 9.3 | 9.4 |  |  |  |  |
|  | 2005 | 30.8 | 9.9 | 36.9 | 27.9 | 12.5 | 28.7 | 31.3 | 3.2 | 13.4 | 11.2 | 12.8 |  |  |  |  |
|  | 2010 | 31.3 | 12.6 | 38.2 | 29.2 | 14.2 | 32.5 | 33.0 | 4.8 | 16.5 | 13.6 | 16.8 |  |  |  |  |
|  | 2015 | 31.4 | 15.6 | 39.2 | 30.7 | 16.4 | 36.4 | 34.7 | 7.1 | 20.0 | 16.3 | 21.4 |  |  |  |  |
|  | 2020 ^a^ | 32.2 | 19.0 | 41.3 | 31.7 | 18.0 | 41.1 | 36.0 | 9.1 | 24.1 | 18.6 | 28.5 |  |  |  |  |
|  | 2025 ^a^ | 32.1 | 22.7 | 42.4 | 32.9 | 20.1 | 45.7 | 37.1 | 12.6 | 28.5 | 21.5 | 37.4 |  |  |  |  |
|  | 2030 ^a^ | 31.6 | 26.8 | 43.1 | 34.1 | 22.2 | 50.4 | 38.0 | 17.4 | 33.2 | 24.5 | 49.4 |  |  |  |  |

^a^ Projected data. More detailed information, such as the prevalence in other years in 1975-2030 and the confidence intervals for the prevalence, are available from the authors.

**Table S8. The SDE's coefficients of the weight mean center of global childhood overweight and obesity**

| year | Boys | | | | |  | Girls | | | | |
| --- | --- | --- | --- | --- | --- | --- | --- | --- | --- | --- | --- |
|  | longitude | latitude | X standard deviation | Y standard deviation | Rotation |  | longitude | latitude | X standard deviation | Y standard deviation | Rotation |
| 1975 | 15.6 | 24.6 | 114.1 | 39.2 | 90.8 |  | 14.0 | 20.2 | 122.7 | 38.9 | 89.9 |
| 1980 | 14.6 | 24.6 | 114.6 | 38.6 | 89.7 |  | 12.9 | 20.0 | 122.5 | 38.3 | 89.6 |
| 1985 | 13.4 | 24.5 | 114.1 | 38.1 | 89.4 |  | 11.8 | 19.7 | 122.2 | 37.8 | 89.3 |
| 1990 | 12.4 | 24.0 | 115.1 | 37.7 | 89.0 |  | 11.1 | 19.2 | 122.3 | 37.4 | 89.1 |
| 1995 | 11.7 | 23.3 | 116.3 | 37.3 | 88.8 |  | 10.4 | 18.5 | 122.5 | 37.0 | 89.0 |
| 2000 | 10.9 | 22.5 | 117.4 | 37.0 | 88.5 |  | 9.7 | 17.9 | 122.5 | 36.6 | 88.9 |
| 2005 | 10.6 | 21.7 | 118.3 | 36.6 | 88.3 |  | 9.5 | 17.2 | 122.3 | 36.3 | 88.8 |
| 2010 | 10.8 | 21.0 | 118.6 | 36.3 | 88.1 |  | 9.7 | 16.7 | 121.6 | 36.0 | 88.8 |
| 2015 | 11.4 | 20.4 | 118.2 | 35.9 | 88.0 |  | 10.1 | 16.3 | 120.5 | 35.7 | 88.7 |
| 2020 | 11.9 | 19.3 | 118.3 | 35.7 | 88.0 |  | 12.2 | 15.8 | 117.9 | 35.2 | 89.2 |
| 2025 | 13.1 | 18.4 | 117.5 | 35.3 | 87.9 |  | 13.0 | 15.1 | 116.9 | 34.9 | 89.1 |
| 2030 | 14.8 | 17.4 | 116.4 | 34.7 | 87.9 |  | 14.1 | 14.4 | 115.5 | 34.5 | 89.1 |

SDE, Standard deviational ellipse.

**Table S9. The SDE's coefficients of the weight mean center of GNI per capita and urbanization rate**

| year | Boys | | | | |  | Girls | | | | |
| --- | --- | --- | --- | --- | --- | --- | --- | --- | --- | --- | --- |
|  | longitude | latitude | X standard deviation | Y standard deviation | Rotation |  | longitude | latitude | X standard deviation | Y standard deviation | Rotation |
| 1975 | 9.8 | 33.4 | 93.5 | 38.6 | 100.6 |  | 15.6 | 23.5 | 97.8 | 36.5 | 89.1 |
| 1980 | 10.4 | 35.1 | 89.8 | 38.9 | 99.8 |  | 15.5 | 23.3 | 97.1 | 36.3 | 89.0 |
| 1985 | 12.4 | 36.2 | 89.8 | 37.2 | 100.8 |  | 15.6 | 23.0 | 96.8 | 36.1 | 88.9 |
| 1990 | 15.7 | 35.4 | 92.3 | 36.1 | 99.6 |  | 15.7 | 22.7 | 96.3 | 36.0 | 88.9 |
| 1995 | 15.7 | 35.6 | 90.4 | 36.1 | 99.2 |  | 15.7 | 22.3 | 96.4 | 35.9 | 88.9 |
| 2000 | 16.7 | 36.1 | 87.0 | 34.8 | 99.8 |  | 15.5 | 22.1 | 96.6 | 35.9 | 88.8 |
| 2005 | 20.7 | 35.5 | 85.0 | 33.3 | 100.4 |  | 15.9 | 21.9 | 96.5 | 35.7 | 88.9 |
| 2010 | 21.5 | 34.5 | 84.9 | 34.2 | 99.6 |  | 16.2 | 21.7 | 96.4 | 35.6 | 88.9 |
| 2015 | 22.2 | 34.2 | 85.6 | 34.7 | 99.7 |  | 16.3 | 21.5 | 96.5 | 35.6 | 89.0 |
| 2018 | 21.4 | 32.7 | 84.9 | 42.8 | 98.8 |  | 16.5 | 21.4 | 96.4 | 35.5 | 89.0 |

**SDE, Standard deviational ellipse**

**Table S10. Lag analysis of the associations between GNI per capita and the prevalence of childhood overweight and obesity**

|  |  | Cutoff value 1 | | | | | | |  | Cutoff value 2 | | | | | | |
| --- | --- | --- | --- | --- | --- | --- | --- | --- | --- | --- | --- | --- | --- | --- | --- | --- |
|  |  | < Mean | | |  | >= Mean | | |  | < 48,000 | | |  | >= 48,000 | | |
|  |  | β | Standardized β | P |  | β | Standardized β | P |  | β | Standardized β | P |  | β | Standardized β | P |
| Boys | GNI | 10.612 | 20.508 | <0.001 |  | -0.537 | -1.038 | 0.063 |  | 3.603 | 6.962 | <0.001 |  | -0.135 | -0.260 | 0.451 |
|  | Higher education enrollment | 0.040 | 0.337 | 0.716 |  | -0.014 | -0.117 | 0.850 |  | 0.050 | 0.426 | 0.583 |  | -0.362 | -3.086 | <0.001 |
|  | CO2 Emissions | -0.243 | -17.104 | 0.039 |  | 0.031 | 2.206 | 0.300 |  | -0.017 | -1.200 | 0.813 |  | 0.207 | 14.594 | <0.001 |
|  | Forest Area | 0.002 | 1.240 | 0.028 |  | 0.005 | 2.843 | 0.890 |  | 0.002 | 1.275 | 0.026 |  | -0.024 | -12.366 | 0.177 |
|  | Meat consumption | 13.800 | 6.734 | 0.205 |  | -3.475 | -1.696 | 0.343 |  | -2.542 | -1.241 | 0.757 |  | 9.752 | 4.759 | 0.018 |
|  | Vegetable and fruit consumption | 2.561 | 2.482 | 0.237 |  | 0.274 | 0.265 | 0.881 |  | 1.266 | 1.227 | 0.428 |  | -2.22 | -21.509 | <0.001 |
|  |  |  |  |  |  |  |  |  |  |  |  |  |  |  |  |  |
| Girls | GNI | 6.368 | 12.306 | <0.001 |  | -0.065 | -0.125 | 0.850 |  | 3.188 | 6.160 | <0.001 |  | -0.896 | -1.732 | 0.037 |
|  | Higher education enrollment | -0.041 | -0.347 | 0.525 |  | -0.311 | -2.654 | 0.001 |  | -0.048 | -0.410 | 0.414 |  | 0.193 | 1.644 | 0.29 |
|  | CO2 Emissions | -0.062 | -4.366 | 0.484 |  | 0.088 | 6.197 | 0.024 |  | 0.010 | 0.713 | 0.846 |  | -0.012 | -0.858 | 0.548 |
|  | Forest Area | 0.001 | 0.456 | 0.197 |  | -0.106 | -54.761 | 0.013 |  | 0.001 | 0.270 | 0.478 |  | 0.038 | 19.670 | 0.439 |
|  | Meat consumption | 9.214 | 4.496 | 0.204 |  | -9.830 | -4.797 | 0.036 |  | -2.632 | -1.284 | 0.675 |  | 7.936 | 3.873 | 0.066 |
|  | Vegetable and fruit consumption | 0.517 | 0.501 | 0.708 |  | 0.875 | 0.848 | 0.705 |  | 1.107 | 1.073 | 0.367 |  | -3.249 | -3.148 | 0.459 |

Analysis of the association between GNI per capita and the prevalence of childhood overweight and obesity with a lag of 4 periods. Cutoff values were chosen based on the mean of GNI per capita and the vertices of the inverted U-shaped relationship between GNI per capita and the prevalence of childhood overweight and obesity. Higher education enrollment (%), CO2 emissions (Ten million tons) and forest area (%) data were extracted from the World Bank, and used to evaluate the cultural level and social environment of the corresponding country. Meat consumption (Ten million tons) and vegetable and fruit consumption (Ten million tons) data were extracted from the Food and Agriculture Organization of the United Nations, and used to evaluate food consumption in the corresponding country.

**Table S11. The prevalence of the childhood obesity based on the results of literature search**

| Country | Year | Age | Prevalence of overweight/obesity | Standard of overweight/obesity |
| --- | --- | --- | --- | --- |
| America ^1^ | 2015–2016 | 6-8 | 58.1% | United States CDC 2000 |
|  |  | 9-11 | 60.4% |  |
|  |  | 12-15 | 68.7% |  |
|  |  | 16-19 | 76.0% |  |
| Canada ^2^ | 2012-2013 | 6-11 | 25.8% | WHO 2006 |
|  |  | 12-17 | 36.8% |  |
| Brazil ^3^ | 2006-2007 | 10-19 | 20.5% | WHO 2007 |
| Colombia ^4^ | 2010 | 5-9 | 18.9% | WHO 2007 |
|  |  | 10-17 | 16.7% |  |
| France ^5^ | 2006 | 6-15 | 15.2% | International Obesity Task Force |
| Russia ^6^ | 2002 | 7-13 | 9.7% | International Obesity Task Force |
| Spain ^7^ | 2011 | 2-15 | 23.7% | Overweight: BMI >= 25 & BMI < 30 Obesity: BMI >= 30 |
| Switzerland ^8^ | 2017-2018 | 6-12 | 15.9% | United States CDC 2000 |
| Australia ^9^ | 2014-2015 | 5-17 | 27.6% | International Obesity Task Force |

The prevalence was extracted from the literature.

References

1 Skinner AC, Ravanbakht SN, Skelton JA, Perrin EM, Armstrong SC. Prevalence of Obesity and Severe Obesity in US Children, 1999-2016. Pediatrics. 2018;141(3):e20173459.

2 Rao DP, Kropac E, Do MT, Roberts KC, Jayaraman GC. Childhood overweight and obesity trends in Canada. Health Promot Chronic Dis Prev Can. 2016;36(9): 194-198.

3 Aiello AM, Marques de Mello L, Souza Nunes M, da Silva AS, Nunes A. Prevalence of Obesity in Children and Adolescents in Brazil: A Meta-analysis of Cross-sectional Studies. Curr Pediatr Rev. 2015;11(1):36-42.

4 Rivera JÁ, de Cossío TG, Pedraza LS, Aburto TC, Sánchez TG, Martorell R.. Childhood and adolescent overweight and obesity in Latin America: a systematic review. Lancet Diabetes Endocrinol. 2014;2(4):321-332.

5 Peneau S, Salanave B, Maillard-Teyssier L, Rolland-Cachera MF, Vergnaud AC, Méjean C, et al. Prevalence of overweight in 6- to 15-year-old children in central/western France from 1996 to 2006: trends toward stabilization. Int J Obes (Lond). 2009;33(4):401-7.

6 Jahns L, Adair L, Mroz T, Popkin BM. The declining prevalence of overweight among Russian children: income, diet, and physical activity behavior changes. Econ Hum Biol. 2012;10(2):139-46.

7 Ajejas Bazan MJ, Jimenez-Trujillo MI, Warnberg J, Domínguez Fernández S, López-de-Andrés A, Pérez-Farinós N. Prevalence of childhood overweight/obesity in Spain 1993-2011 and associated risk factors in 2011 Prevalencia de sobrepeso y obesidad infa. Nutr Hosp. 2018;35(1):84-9.

8 Herter-Aeberli I, Osuna E, Sarnovska Z, Zimmermann MB. Significant Decrease in Childhood Obesity and Waist Circumference over 15 Years in Switzerland: A Repeated Cross-Sectional Study. Nutrients. 2019;11(8):1922.

9 Huse O, Hettiarachchi J, Gearon E, Nichols M, Allender S, Peeters A. Obesity in Australia. Obes Res Clin Pract. 2018;12(1):29-39.


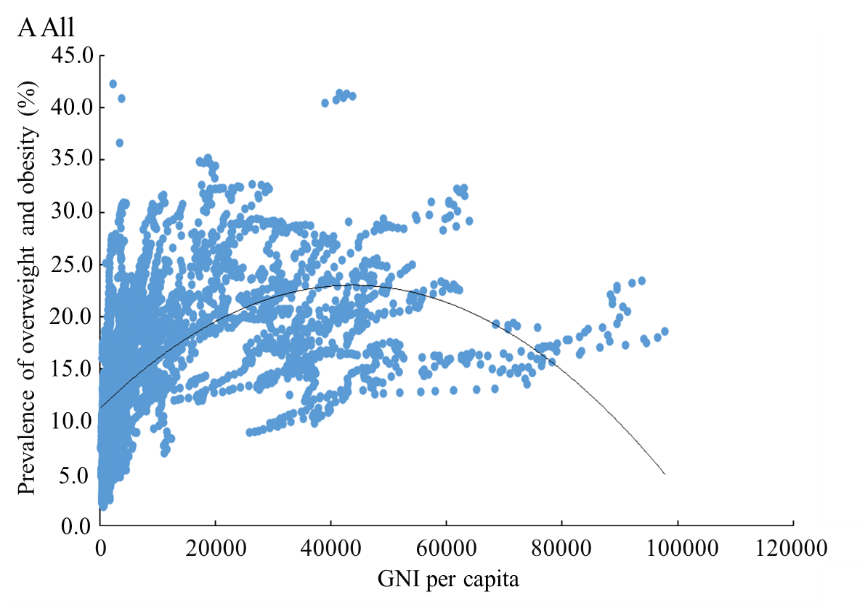


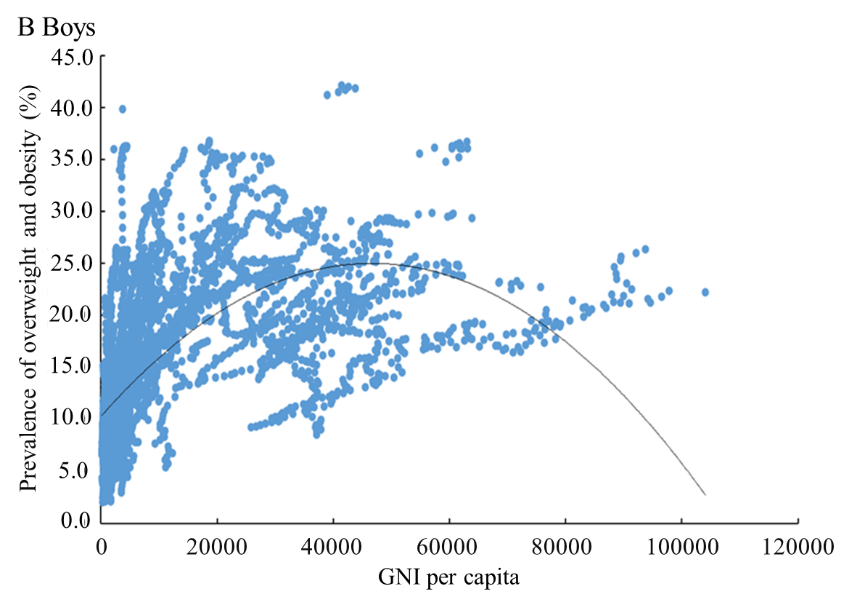


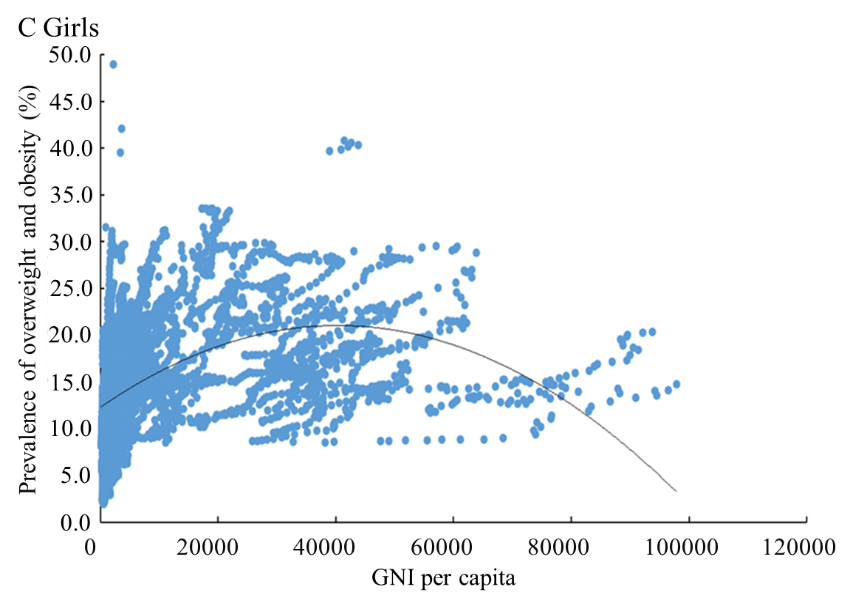


**Figure S1 Scatterplot of GNI per capita and childhood overweight and obesity.** Scatter plot of the relationship between GNI per capita and the prevalence of childhood overweight and obesity in 149 countries from 1975 to 2018. Black line is the scatter fitting curve based on quadratic equation.


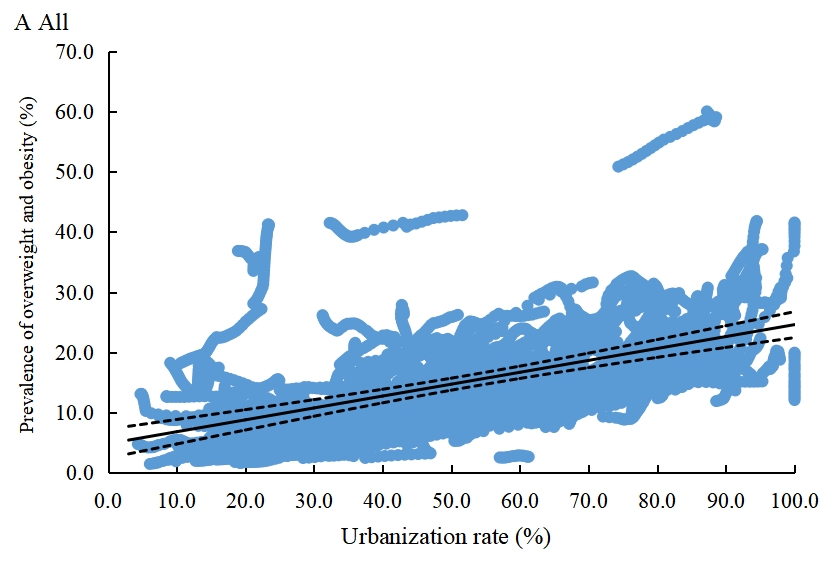


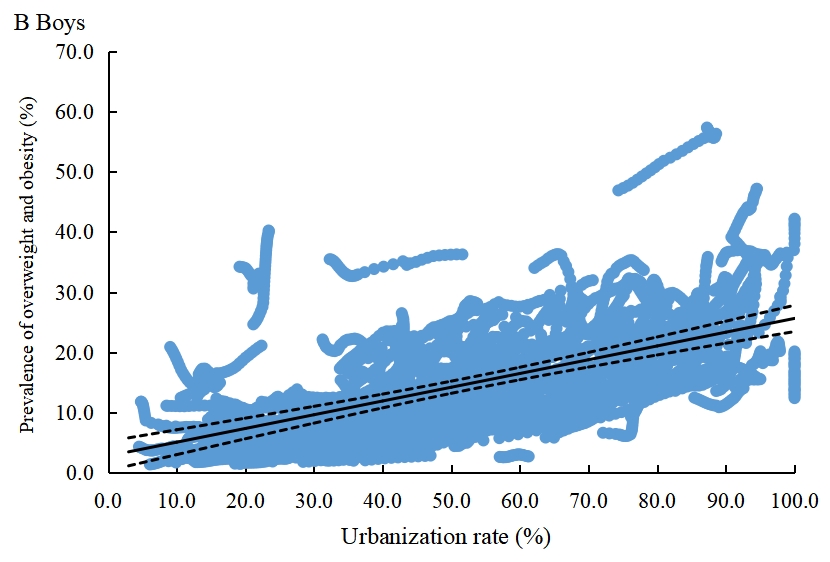


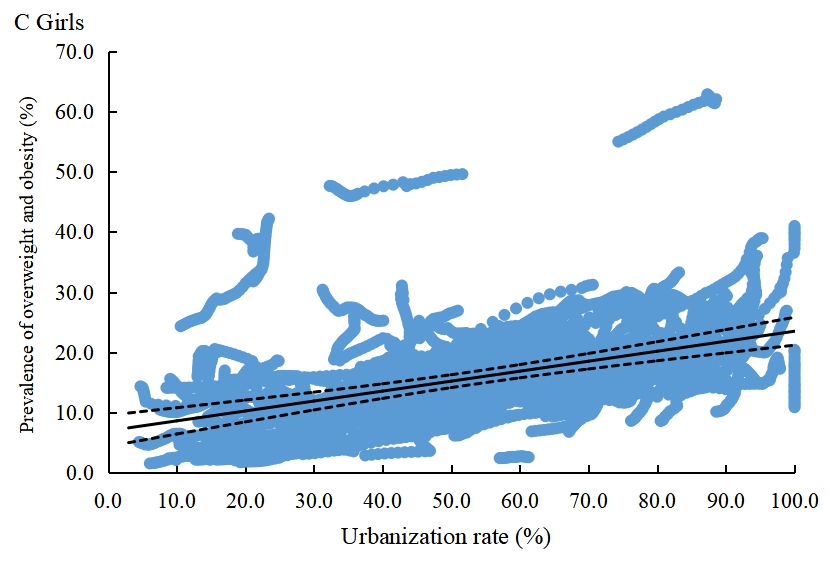


**Figure S2 Scatterplot of urbanization rate and childhood overweight and obesity.** Scatter plot of the relationship between urbanization rate and the prevalence of childhood overweight and obesity in 190 countries from 1975 to 2018. Black line is the scatter fitting curve based on linear equation. Dashed line is the 95% confidence interval.
